# Whole-Genome Pathway Analysis on 132,497 Individuals Identifies Novel Gene-Sets Associated with Body Mass Index

**DOI:** 10.1371/journal.pone.0078546

**Published:** 2014-01-31

**Authors:** Matthew A. Simonson, Matthew B. McQueen, Matthew C. Keller

**Affiliations:** 1 Department of Psychology and Neuroscience, Institute for Behavioral Genetics, University of Colorado, Boulder, Colorado, United States of America; 2 Department of Integrative Physiology, Institute for Behavioral Genetics, University of Colorado, Boulder, Colorado, United States of America; 3 Department of Psychology and Neuroscience, Institute for Behavioral Genetics, University of Colorado, Boulder, Colorado, United States of America; 4 Mayo Clinic, Department of Health Sciences, Division of Biomedical Statistics and Informatics, Rochester, Minnesota, United States of America; Democritus University of Thrace, Greece

## Abstract

Whole genome pathway analysis is a powerful tool for the exploration of the combined effects of gene-sets within biological pathways. This study applied Interval Based Enrichment Analysis (INRICH) to perform whole-genome pathway analysis of body-mass index (BMI). We used a discovery set composed of summary statistics from a meta-analysis of 123,865 subjects performed by the GIANT Consortium, and an independent sample of 8,632 subjects to assess replication of significant pathways. We examined SNPs within nominally significant pathways using linear mixed models to estimate their contribution to overall BMI heritability. Six pathways replicated as having significant enrichment for association after correcting for multiple testing, including the previously unknown relationships between BMI and the Reactome regulation of ornithine decarboxylase pathway, the KEGG lysosome pathway, and the Reactome stabilization of P53 pathway. Two non-overlapping sets of genes emerged from the six significant pathways. The clustering of shared genes based on previously identified protein-protein interactions listed in PubMed and OMIM supported the relatively independent biological effects of these two gene-sets. We estimate that the SNPs located in examined pathways explain ∼20% of the heritability for BMI that is tagged by common SNPs (3.35% of the 16.93% total).

## Introduction

Obesity greatly increases risk for many forms of pathology, including vascular disease, multiple forms of cancer, heart disease, and other serious health problems [1], [2]. A greater understanding of the biology underlying obesity could therefore have widespread effects on public health. This has led to large-scale efforts to understand the genetic architecture of obesity through the application of genome-wide association studies and complementary methods, such as pathway analysis [3]–[7].

In 2010, the GIANT Consortium (Genetic Investigation of ANthropometric Traits) performed the largest GWAS of BMI to date, a two-stage analysis on 249,796 individuals of European ancestry [8]. During the first stage, GIANT conducted a meta-analysis using data from 46 studies including 123,865 subjects and identified 42 independent loci associated with BMI at *P*<5×10^−6^. During stage two, 125,931 subjects from 34 additional studies were used examine the 42 loci with suggestive significance in the first stage. In a joint analysis of the first and second stage 32 SNPs were significantly associated with BMI at *p*<5×10^−8^, increasing the number of loci robustly associated with BMI from 10 to 32 [3]–[8]. The GIANT study examined biological pathways that contain one or more genes located within 300 kb of the 32 confirmed BMI SNPs in an attempt to discover potentially new pathways associated with BMI, and to test whether the 32 confirmed association’s clustered near genes with biological relevance (Table 1) [8].

**Table 1:** Pathways with significant enrichment for associations of the top 25% of SNP associations detected by GIANT Consortium.

Database	Pathway	Nominal GSEAp-value	FDR q-value	Genes within 300 kb fromconfirmed BMI SNPs
Panther	PDGF SIGNALING PATHWAY	0.0008	0.0061	SPDEF
Panther, BP	PROTEIN PHOSPHORYLATION	0.0001	0.0453	DMPK; PRKD1; MAP2K5; COL4A38P; PACSIN1; TNNI3K; STK33; FLJ40125
Panther, MF	HOMEOBOX TRANSCRIPTION FACTOR	0.0001	0.0110	MEIS3; IRX3; SIX5
Panther, MF	TRANSLATION ELONGATION FACTOR	0.0008	0.0066	TUFM
Gene Ontology, BP	NEUROGENESIS	0.0001	0.0214	NRXN3; RACGAP1
Gene Ontology, BP	NEURON DIFFERENTIATION	0.0001	0.0324	NRXN3
Gene Ontology, BP	GENERATION OF NEURONS	0.0002	0.0335	NRXN3; RACGAP1
Gene Ontology, BP	REGULATION OF CULLULARMETABOLIC PROCESS	0.0002	0.0308	ERCC1; FOSB; GRLF1; HMGA1; SMARCD1; MTIF3
Gene Ontology, MF	HORMONE RECEPTOR BINDING	0.0002	0.0082	HMGA1
Gene Ontology, MF	NUCLEAR HORMONE RECEPTOR BINDING	0.0005	0.0085	HMGA1

Pathway databases include Panther, Panther Biological Processes (BP), Panther Molecular Function (MF), Gene Ontology Biological Processes (BP), and Gene Ontology Molecular Function (MF).

Exclusively analyzing pathways that contain significant individual SNP associations in a discovery set is an informed way to reduce the number of pathways being examined and decrease the rate of type II errors from among those pathways implicated by SNPs detected in the discovery set [9]–[12]. However, a major drawback of the candidate pathway approach is that it can result in an overly restricted exploration of the genome and lead to an inflation of type-II errors genome-wide. Significant pathways and their relevant biological functions can remain undetected because pathways with an over-representation of associated SNPs that individually fail to meet stringent genome-wide significance levels are excluded from analysis. For highly polygenic traits like BMI that appear to be influenced by numerous loci in several different regions, this can be especially problematic [13]–[15].

Alternatively, formal whole-genome pathway analysis has a much less restricted scope, examining pathways composed of SNPs within genes from across the entire genome [16]–[18]. The results from studies of traits such as schizophrenia, diabetes, Chrohn’s disease, arthritis, and several others demonstrate that the whole genome approach can detect significantly enriched pathways that do not contain individually significant SNPs [19]–[23].

The current study employed Interval Based Enrichment Analysis (INRICH) to perform whole-genome pathway analysis of body-mass index (BMI) [24]. We decided to apply INRICH, a relatively new method of pathway analysis, because (a) previous research shows reduced Type I and Type II error rates for this algorithm compared to other methods that use the gene-set enrichment approach [9]–[11], [25], [26]; (b) it has previously been used to successfully identify pathways across multiple phenotypes [9]–[11], [25], [26]; and (c) it uses summary data from SNP associations and does not require the original genotype data, which was necessary for conducting the pathway analysis on the GIANT data. We downloaded gene set annotation for 880 canonical pathways from the Molecular Signatures Data-base (MSigDB version 3.7) [27]. Most pathway databases are organized in a hierarchical structure, resulting in a high degree of overlap between gene-sets. The MSigDB database was designed to attenuate the problem of gene overlap between pathways by removing gene-sets that have the same member genes with their parent nodes and sibling nodes, maximizing the independence of gene-sets while still maintaining much of the information about the functional interrelationships between pathways [27], [28]. Our analysis used all publically available summary statistics from the GIANT Consortium’s stage 1 meta-analysis of 123,865 individuals of European Ancestry (EA) as a discovery set to identify nominally significant pathways. We then validated the significance of detected enrichment using three publicly available datasets that contained a total of 8,632 EA subjects.

We also examined gene overlap between significant pathways to gain a better understanding of the biological networks that influence BMI. Genes and their products often act in multiple pathways, meaning some degree of overlap is expected [29]. The INRICH method corrects for potential bias introduced by non-independence between pathways and also prevents a small number of genes from driving pathways to significance [24]. Because of these corrections, our study treated genes shared by multiple significant pathways as potential sources of insight into important biological components relevant to BMI [25], [29]–[31].

Regions of the genome within significant pathways may have a greater than expected influence on the heritability of a trait because pathways contain sets of genes with shared biological functions [33]–[35]. Previous studies demonstrate that ∼16–17% of BMI heritability is explained when all common SNPs from across the genome are examined as a set, and a significantly disproportionate amount of that variation (∼9.9% of the heritability) exists in genic regions [8], [36]. We used linear mixed models to identify sets of genes with excessive influence on the heritability of BMI [13], [36].

## Results

Initially, we performed whole-genome pathway analysis using Interval Based Enrichment Analysis (INRICH) to identify pathways that were significantly enriched for SNP associations in the GIANT discovery set. The first stage in INRICH analysis generates interval data based on patterns of linkage disequilibrium to construct independent regions of association. We used HapMap Phase 2 European-American as a reference panel for patterns of linkage-disequilibrium (LD), the same reference GIANT used to perform imputation on the original data [37]. Next, we used INRICH to identify pathways that contained an excess of associations at four commonly used thresholds for SNP associations: the top 0.5%, 1.0%, 5.0%, and 10.0% of SNP associations [24], [25], [33]. Only SNPs surpassing these thresholds were included in determining whether pathways were enriched with ‘significant’ SNPs. The threshold values selected were purposefully liberal compared to typical genome-wide thresholds, which allowed us to detect the influence of pathways in which several genes show moderate associations, rather than a small number of genes with large effects that are better detected using more stringent thresholds [25], [33], [38].

At SNP α thresholds of 10.0%, 5%, 1%, and 0.5%, we identified 85, 51, 35, and 20 nominally significant pathways respectively (Table S1–S4) in the GIANT discovery set. The nominal p-value returned by INRICH indicates the probability of observing the amount of overlap that exists between BMI associated intervals and a given pathway gene set under the null hypothesis of no true enrichment for associations, not correcting for multiple testing [24]. Based on a type-I error rate of 0.05 and assuming independence between pathways (see below), the expected number of nominally significant pathway associations under the null hypothesis was 27, 27, 23, and 15, of the 535, 533, 465, and 304 pathways examined, demonstrating an excess of pathways with significant enrichment beyond what was expected by chance at more liberal SNP inclusion thresholds (exact binomial test p-values of <2.2e-16, 1.44e-5, .018, and.23 respectively). These exact binomial p-values should be treated with caution because they do not account for the dependencies among pathways, but they are consistent with the idea that (a) BMI is influenced by the cumulative effect of a large number of small-effect SNPs that act within pathways and (b) analyses designed to detect the effects of a more modest number of larger-effect SNPs (e.g., using a.005 SNP α threshold or using only genome-wide significant SNPs) are likely to miss many truly associated pathways.

To winnow down the 191 nominally significant pathways identified in the GIANT discovery set to a smaller number of more robustly associated pathways, we used an independent replication set of 8,632 individuals to validate only those pathways detected as nominally significant in the initial analysis. In total, 47 of these pathways replicated as nominally significant (p<0.05) of the 191 examined: 23 of 85 at the 10% SNP threshold, 16 of 51 at the 5% SNP threshold, 5 of 35 at 1% SNP threshold, and 3 of 20 and 0.5% SNP threshold (Tables S5–S8). The number of nominally significant pathways were significantly higher than expected under the null hypothesis (exact binomial test p-values for the four thresholds were 1.98e-11, 2.03e-9, .029, and .076 respectively). As above, the exact binomial test p-values do not account for dependencies among pathways and so are overly liberal, but they again suggest that SNP effects in biologically relevant pathways are likely to be individually minor and highly distributed.

To determine which of the 47 pathways that replicated (*p*<.05) in the replication sample were significant after accounting for multiple testing, dependencies between pathways, and characteristics (e.g., numbers of genes) of each pathway, we used the permutation approach employed in INRICH. INRICH compares the observed nominal p-values of pathways to a null distribution composed of the minimum nominal p-value observed across the 47 examined pathways from each iteration of a permutation [24]. Of the 47 pathways that were nominally significant in both the replication and discovery sets, six pathways were significantly associated with BMI after correcting for multiple testing and pathway dependencies (see Table 2). Three of the six significant pathways did not contain genes that were investigated during the candidate pathway analysis performed by the GIANT Consortium and are novel pathway associations (Table S9 & Supplementary Table 5 of [39]). Specifically, the Reactome regulation of ornithine decarboxylase pathway (corrected *p* = 0.038), and the Reactome stabilization of P53 pathway (corrected *p* = 0.048), were significantly enriched for associations from the top 5% of SNPs and were not previously associated with BMI. The KEGG Lysosome pathway was enriched for associations from both the top 1% (corrected *p* = 0.016), and the top 0.5% of SNPs (corrected *p* = 0.043), which shows this pathway contained an excess of loci with relatively large effects that were distributed across the top 0.5% and top 1% of SNP associations. Of the pathways that did contain genes examined in previous studies, the KEGG Toll-like receptor-signaling pathway (corrected *p* = 0.049), and the KEGG Fc epsilon RI signaling pathway (corrected *p* = 0.025) were identified as enriched for associations from the top 10% of SNPs, enrichment at this threshold in combination with lack of significant enrichment at more stringent thresholds indicates these pathways contained an excess of loci with relatively small effects. The Signal Transduction KE ERK1/ERK2 MAPK pathway (corrected *p* = 0.041) was enriched for associations from the top 5% of SNPs, demonstrating enrichment for loci with relatively moderate effects compared to the other thresholds examined. Regional association plots for intervals in all significant pathways are presented in Figures S1–S7.

**Table 2:** Pathways with significant enrichment for associations in the replication set after correcting for multiple testing.

Database	Pathway	Min. nominalp-value	Significantlyenrichedthresholds	Correctedp-value	Number ofgenes	Genes within 300 kb fromconfirmed BMI SNPs
KEGG	Fc epsilon RI signalingpathway	0.0005	Top 10%	0.025	79	LAT
KEGG	* Lysosome pathway	0.0051	Top 0.5%, Top 1%	0.0437	121	
KEGG	Toll-like receptor-signaling pathway	0.0024	Top 10%	0.0499	102	NFKB1
Reactome	* Regulation of ornithine decarboxylase pathway	0.0024	Top 5%	0.0383	49	
Reactome	* Stabilization of P53pathway	0.0027	Top 5%	0.0481	45	
SignalTransduction KE	ERK1/ERK2 MAPK	0.0026	Top 5%	0.0413	32	NFKB1

Pathways with novel detected enrichment have a * next to the name and contain no genes listed in the far right column.

We investigated the overlap of genes between these six significantly associated pathways too better understand their inter-relationships. Two non-overlapping groups of pathways (pathways that do not share common genes) emerged. Details on the overlap at the gene level between significant pathways are provided in Table 3. In the first set, the KEGG Fc epsilon RI signaling pathway, the KEGG Toll-like receptor-signaling pathway, and the Signal Transduction KE ERK1/ERK2 MAPK pathways all shared a large number of genes, while the KEGG Lysosome pathway shared a single gene with the KEGG Toll-like receptor-signaling pathway. The second group of pathways included the Reactome stabilization of P53 pathway, and the Reactome regulation of ornithine decarboxylase pathway.

**Table 3:** Genes shared by significant pathways.

Pathway Genes Overlap	1	2	3	4	5	6
**(1)** KEGG Fc epsilon RI signaling pathway	79	0.00%	30.90%	0.00%	0.00%	12.60%
**(2)** KEGG Lysosome pathway	0	121	0.90%	0.00%	0.00%	0.00%
**(3)** KEGG Toll-like receptor-signaling pathway	28	1	102	0.00%	0.00%	10.45%
**(4)** Reactome regulation of ornithine decarboxylase pathway	0	0	0	49	82.90%	0.00%
**(5)** Reactome stabilization of P53 pathway	0	0	0	39	45	0.00%
**(6)** Signal Transduction ERK1/ERK2 MAPK	7	0	7	0	0	32

Total number of genes in each pathway is presented on the diagonal, percentages of overlap are presented in top right half, and number of genes shared is presented in bottom left half.

**Figure 1:**
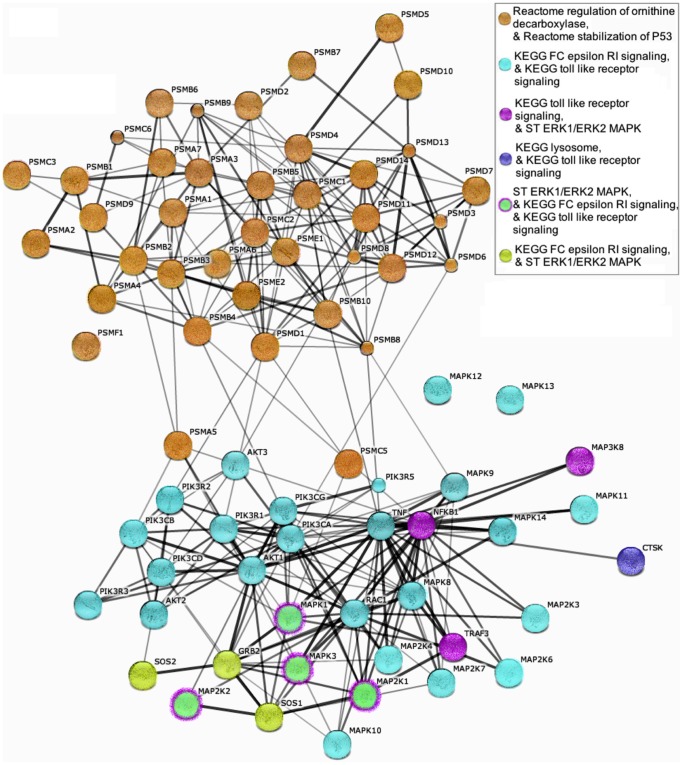
Plot generated using STRING 9.0 (Search Tool for the Retrieval of Interacting Genes). Previously identified protein-protein interactions among genes that are shared across significant pathways. Black edges represent interactions; line thickness is a function of number of previously identified interactions.

After investigating the degree of gene overlap between significant pathways, we used STRING 9.0 (Search Tool for the Retrieval of Interacting Genes) to examine previously identified protein-protein interactions among genes that were shared across significant pathways [32]. We input a list of all genes that were located in more than one of the six pathways into STRING 9.0 then computed clusters based on previously identified protein-protein interactions listed in PubMed and OMIM, and mapped them to each of the listed genes. Two clusters of functionally related genes emerged, demonstrating relatively independent biological effects of the two sets of genes. The clusters were highly concordant with the gene overlap we identified between pathways, as well as the division between novel pathways identified in this analysis and the pathways identified in the candidate pathway analysis performed by the GIANT Consortium (Figure 1).

We used the GCTA software package to estimate SNP based heritability using linear mixed models in order to determine if SNPs within the examined pathways explained a disproportionate amount of the heritability for BMI [40]. We first generated a genetic relationship matrix between all individuals in the sample using the SNPs located within genes from all 535 examined pathways. We then generated a separate genetic relationship matrix using the remaining SNPs in the genome. GCTA partitioned how much variation in BMI was explained by SNPs inside and outside of pathways by examining the relationship between pairwise genetic and phenotypic similarity by fitting both genetic relationship matrices simultaneously using restricted maximum likelihood (REML) estimation maximization algorithm. We found that SNPs within all examined pathways explained 3.35% of BMI heritability (s.e. = 1.68%, p = 0.047), which is equivalent to 19.76% of the total variance explained by common SNPs. This percentage (19.76%) was greater than the proportion of the genome represented in these pathways (13.06%), but this difference was not significant, which is not surprising given the large standard errors of the estimate (19.76% vs. 13.06%, s.e. = 9.13%, p = 0.463) (Table 4).

**Table 4:** Partitioned heritability estimates.

Sample	Variance Explained	Total Variance	Heritability	Heritability s.e.	P-value	Size (BP)	Percent of Genome
All Pathways	0.76	22.80	3.35%	1.69%	0.05	376226374	13.06%
Top 10% pathways	0.22	22.81	0.96%	0.87%	0.27	99482480	3.45%
Top 5% Pathways	0.30	22.81	1.31%	0.85%	0.12	94798221	3.29%
Top 1% Pathways	0.06	22.81	0.28%	0.73%	0.70	82971589	2.88%
Top 0.5% Pathways	<0.01	22.80	<0.01%	0.52%	>0.99	38151603	1.32%
Not in Pathways	3.10	22.81	13.58%	3.46%	0.00	2504806912	86.94%
Total SNPs	3.86	22.81	16.93%	3.61%	0.00	2881033286	100.00%

Heritability estimates and proportion of genome represented in 1.) all pathways, 2.) significant pathways at each enrichment threshold, 3.) outside of pathways, 4.) total SNPs in genome, 5.) genes located within 300 KB of 32 confirmed BMI SNPs.

## Discussion

Six pathways contained significant enrichment for associations with BMI after correcting for multiple testing. Three of these pathways did not contain genes located near previously associated loci. Two non-overlapping gene-sets emerged when we compared which genes were contained within the six significant pathways. Gene clusters based on identified protein-protein interactions listed in PubMed and OMIM suggested that the genes within the significant pathways fit into two sets of relatively independent biological effects. These two sets were highly concordant with the groups of genes shared between the six significant pathways, as well as the division between pathways containing loci that were previously associated with BMI and the novel pathway associations identified in this analysis (Figure 1, Table 2).

We identified novel associations between the Reactome regulation of ornithine decarboxylase pathway, and the Reactome stabilization of P53 pathway with BMI. Recent studies have found a relationship between the P53 tumor suppressor protein and adipogenic differentiation between white and brown fat cells, and has been directly implicated in protection against diet-induced obesity in both mice and humans [41], [42]. White adipose tissue plays a significant role in energy storage and regulation of energy balance, while brown adipose tissue’s principal function is generation of heat by fat burning [43], [44]. Results from several studies indicate that there is an inverse relationship between brown adipose tissue activity and obesity [45]–[47]. Also, the polyamine products of the ornithine decarboxylase pathway are associated with increased cell growth and reduced apoptosis [48].

Our results also provide support for the GIANT consortium‘s finding that pathways containing genes near significant loci are more likely to contain other loci with greater effects than is expected by chance. Pathways that were part the second protein-protein interaction cluster, which contained genes previously associated with BMI, demonstrated the positive relationship between functional clustering, increased enrichment for novel associations, and previously detected significant loci. Specifically, The KEGG Toll-like receptor-signaling pathway and the Signal Transduction KE ERK1/ERK2 MAPK pathway shared seven genes, including the gene NFKB1. SNPs near NFKB1 were previously associated with BMI, and we found it was one of the most highly connected genes in the protein-protein interaction cluster [32]. Genes from the KEGG lysosome pathway shared functional relationships with several of the pathways that contained previously implicated loci, even though the pathway itself did not contain any, and only shared a single gene with the KEGG toll-like receptor-signaling pathway. The KEGG Toll-like receptor-signaling pathway shared the gene CTSK with the KEGG lysosome pathway. While CTSK did not contain previously identified SNPs associated with BMI, studies have demonstrated up regulation of this gene in the white adipose tissue of overweight/obese subjects and have found that up-regulation has a significant positive correlation with BMI [49].

Some limitations should be noted when interpreting our results. First, although our analysis indicates statistically enriched association of SNPs within multiple pathways, and determined that SNPs within the examined pathways explained a significant proportion of the heritability for BMI, we were unable to determine the contribution of individual pathway gene-sets to BMI heritability, or whether specific gene-sets explained a greater than expected proportion of the heritability due to lack of power. Future studies may be able to increase power by applying a method recently developed by Jian Yang et al. that estimates the combined effect of multiple SNPs on the heritability of a trait using only summary statistics [50]. A second limitation of our analysis is due pathway and protein-protein interaction annotation being incomplete. Several thousand genes are not yet included in any pathway annotation databases; this results in all non-annotated genes being automatically excluded from analysis. Our analysis of identified protein-protein interactions was dependent upon interactions listed in PubMed and OMIM. Due to current limits in knowledge of human genes and their regulation the information in any database is far from complete. Additionally, while our results provide compelling evidence for the polygenic structure of the genetic architecture underlying BMI, they do not pinpoint the exact loci where risk variants reside within the genome. The use of pathway analysis, as well as linear mixed models to perform SNP-set based analysis results in not knowing the exact locations of the individual SNPs underlying significant effects [28], [51].

In summary, we examined summary statistics from a meta-analysis of 123,865 subjects performed by the GIANT Consortium, and a sample of 8,632 subjects to assess independent replication of pathways identified as having significant enrichment of association. Six pathways contained significant enrichment for associations with BMI after correcting for multiple testing. The Reactome regulation of ornithine decarboxylase pathway, the KEGG lysosome pathway, and the Reactome stabilization of P53 pathway are novel pathway associations with physiological effects that are relevant to BMI. These results demonstrate that whole-genome pathway analysis can detect significantly enriched pathways that do not contain specific candidate genes or individually significant SNPs. Our results also provide further evidence for the highly polygenic structure of BMI, and identify the relative contribution of SNPs within pathway gene-sets to BMI heritability. We demonstrate how network-based approaches that combine the results of pathway analysis with protein-protein interaction information can be used to gain a better understanding of the biological connections that influence BMI. Intriguingly, we show significant convergence of key genes and biological functions being broadly involved in regulation of growth and metabolism through the application of different methods of genetic analysis. This may be of significant diagnostic and therapeutic importance. More conclusive interpretation of individual loci will require more focused regional analysis, such as sequencing. For further investigation of these pathways in independent datasets, we propose testing a model that includes investigation of the effects of rare-variants and other genetic models (e.g. epistasis, recessive effects). In combination with targeted DNA sequencing studies, this may reveal the impact of discrete molecular pathways on risk for many forms of pathology, including obesity, multiple forms of cancer, cardiovascular disease, and other serious health problems. Further functional work is required in particular to investigate the role of adipogenic differentiation between white and brown fat cells, up regulation of white fat cells, and increased cell growth/decreased apoptosis, given the growing convergence across studies of metabolic regulation on these mechanisms.

## Methods

### Summary Statistics of Meta-analysis Data

The discovery set in our analysis was composed of publicly available summary statistics from a meta-analysis of 46 GWAS of BMI performed by the GIANT Consortium, a total sample of 123,865 individuals of European ancestry (http://www.broadinstitute.org/collaboration/giant/index.php/GIANT_consortium_data_files) [8]. Imputation was originally performed on all included datasets for ∼2.8 million SNPs using HapMap Phase 2 European-American as a reference panel [37]. We removed SNPs with recorded sample sizes >2 s.d. from the mean (number of samples from meta-analysis that were genotyped at a given SNP), and also excluded SNPs with MAF <0.01. We then extracted the 463,139 SNPs that were common to both the discovery set and post QC replication set to minimize any differences between discovery and replication data.

### Replication Subjects and Phenotype Information

The replication set in this study was derived from all publicly available SNP data that measured BMI and that was not included in the discovery sample: the NHLBI Multi-Ethnic Study of Atherosclerosis (MESA) SNP Health Association Resource (SHARe), the GENEVA Genes and Environment Initiatives in Type 2 Diabetes (Nurses’ Health Study/Health Professionals Follow-up Study), and the Coronary Artery Risk Development in Young Adults (CARDIA) Study – Gene Environment Association Studies Initiative (GENEVA), as available through NCBI’s database of Genotypes and Phenotypes (dbGaP). Information on genotypes (Affymetrix 6.0), phenotypes, and environmental variables from 8,632 individuals was used from across all three studies (population trait statistics are available in Table S11). We selected these studies because they all had BMI phenotype information, were also genotyped on the Affymetrix 6.0 platform, and were not included in the previous analysis performed by the GIANT Consortium.

The MESA study is a prospective population-based study of the characteristics of subclinical cardiovascular disease (disease detected non-invasively before it has produced clinical signs and symptoms) and the risk factors that predict progression to overt cardiovascular disease [52]. The sample is composed of 6,814 men and women aged 45–84 who were asymptomatic for cardiovascular disease, drawn from 6 field centers across the United States (Wake Forest University, Columbia University, Johns Hopkins University, University of Minnesota, Northwestern University and University of California - Los Angeles). BMI measurements were recorded, along with other clinically relevant information. Blood for DNA extraction was drawn and participants consented to genetic testing. After taking into account availability of adequate amounts of high quality DNA, appropriate informed consent and genotyping quality control procedures, genotype data was available for 1,991 individuals of European ancestry.

The GENEVA Type 2 Diabetes (NHS and HPFS studies) are prospective cohort studies of type 2 diabetes, body mass index, and several related phenotypes in 121,700 female registered nurses between the ages of 30–55 years at baseline in 1976, and 51,529 male health professionals between the ages of 40–75 years at baseline in 1986 respectively [53], [54]. BMI measurements were recorded, along with other clinically relevant information every two years. Blood for DNA extraction was drawn from 6016 subjects between 1989 and 1995 and participants consented to genetic testing in 2007–2008. After taking into account availability of adequate amounts of high quality DNA, appropriate informed consent and genotyping quality control procedures, genotype data was available for 5,445 individuals of European ancestry.

The CARDIA study is a prospective, multi-center investigation of the natural history and etiology of cardiovascular disease between the ages of 18 to 30 at the time of initial examination [55]. BMI measurements from subjects were recorded, along with other medical variables of interest. The CARDIA sample was drawn from populations in Birmingham AL, Chicago IL, and Minneapolis MN and, in Oakland, CA. The initial examination included 5,115 participants selectively recruited to represent proportionate racial, gender, age, and education groups from each acquisition site. DNA extraction for genetic studies was performed at the year 10 examination using blood drawn at the baseline exam. After taking into account availability of adequate amounts of high quality DNA, appropriate informed consent and genotyping quality control procedures, genotype data was available for 1,196 individuals of European ancestry.

### Data Cleaning and Quality Control

The first stage of data cleaning involved using PLINK, the whole genome association analysis toolset, in combination with R statistical computing software, to perform quality control procedures on all three samples included in the replication set separately [56], [57]. After cleaning was performed within each dataset separately, all replication set data was merged and the same cleaning procedures were performed again on the merged sample to ensure the total sample met stringent quality control standards.

Subjects were excluded if genotyping rates were less than 95%. Individuals were also excluded if the predicted sex based on X-chromosome genotypes did not match the recorded sex. Subjects who were outliers with respect to estimated heterozygosity, those greater than 3 standard deviations from the mean, were excluded. All close relatives of individual subjects, based on mean identity-by-descent (IBD; PIHAT in PLINK) values indicating relatedness of less than 2^nd^ degree relatives, were excluded from the sample. Visual inspection of Multidimensional scaling (MDS) plots was used to remove outliers with respect to ancestry. Markers were excluded if (1) genotyping rates were less than 95%, (2) minor allele frequencies were less than 0.01, and (3) if p-values from the Hardy-Weinberg Equilibrium (HWE) test were less than 1×10^−4^. We also removed individuals who had missing values for any covariates or phenotypic data. This resulted in a total of 8,632 unrelated European ancestry individuals that met all cleaning thresholds across all samples included in the replication set. The physical positions of all SNPs were updated to ensure concordance across datasets and compatibility with pathway annotation using the hg18 assembly of the human genome. We then extracted the 463,139 SNPs that were common to both the discovery set and each of the replication sub-sets to minimize any differences between samples used in the analysis.

A log-transform of BMI was performed to adjust for BMI not being normally distributed, [58], [59]. To control for potential confounds, multiple regression examined the relationship between the log-transformed values of BMI and dataset, age, sex, genotyping batch effects, and the first 10 principal components to control for the effects of population stratification. The residual for each subject was then used as the phenotype for all analyses.

### Replication Set GWAS

A genome-wide association analysis was performed on all SNPs using the residualized BMI phenotype as the target outcome. Using the PLINK software package (v1.07) with the linear models option, a linear regression test was performed on all quality controlled SNP data using 8,632 individuals genotyped at 463,139 loci [56]. An additive mode of inheritance was assumed and empirical p-values were generated for association with the quantitative phenotype at each locus. A Manhattan plot and a Quantile-Quantile (Q-Q) plot were used to visualize association results (Figures S8–S9). Prior to the analysis, we adopted the genome-wide significance threshold of p<5×10^−8^ to account for multiple testing [60].

### Pathway Analysis Methods

We used Interval Based Enrichment Analysis (INRICH) to identify pathways that were significantly enriched for SNP associations at four commonly used cutoffs of the top 0.5%, 1.0%, 5.0%, and 10.0% of SNP associations from the 463,137 SNPs included in our analysis [11], [18], [24], [61]. Because the choice of an enrichment threshold is arbitrary and the optimal cutoff was unknown, we chose a range of cutoffs [25]. The values we selected were not highly stringent, meaning they were more likely to detect the influence of pathways in which several genes show moderate associations, rather than a small number of genes with large effects that are better detected using more stringent thresholds [38]. We focused on detecting pathways with relatively small and more distributed effects because the influence of several associations with large individual effects was already detected by the GIANT Consortium [8].

Gene set annotation was downloaded from the Molecular Signatures Data-base (MSigDB version 3.7), for 880 canonical pathways [27]. Most pathway databases are organized in a hierarchical structure, resulting in a high degree of overlap between gene-sets. The MSigDB database was designed to attenuate the problem of gene overlap between pathways by removing gene-sets that have the same member genes with their parent nodes and sibling nodes, maximizing the independence of gene-sets while still maintaining much of the information about the functional interrelationships between pathways [27], [28]. Canonical pathways are representations of biological processes compiled from multiple databases including KEGG, GO, BioCarta, Signal Transduction Knowledge Environment (KE), and REACTOME [62], [63]. To reduce the multiple testing burden and to avoid testing overly broad or narrow functional categories, we only tested pathways that contain between 20 and 200 representative genes [64].

Analysis using INRICH involves three stages: (1) generate interval data based on patterns of linkage disequilibrium to construct highly independent regions of association; (2) identify nominal enrichment using an interval-based permutation strategy; and (3) perform a second round of permutation to correct for multiple testing at the pathway level [24].

A list of LD-independent associated genomic regions was generated for the replication set using the observed SNP associations and patterns of linkage-disequilibrium present in the data. In the discovery set, LD-independent associated genomic regions were identified using summary-level statistics from the BMI meta-analysis performed by GIANT in combination with a reference panel to estimate patterns of LD. HapMap Phase 2 European-American was used as a reference panel, the same reference used by GIANT to perform imputation on the original data [37]. The PLINK LD clumping option was used to generate lists of highly independent associated genomic regions in the discovery and replication sets at each enrichment threshold (*clump-p1 = threshold; clump-p2 = 1; clump-r2 = 0.2; clump-kb = 250*). The values selected match those from previous studies that identified LD-independent associated regions using PLINK’s LD clumping option when examining the same p-value cutoff thresholds that we used [61], [65]. INRICH calculated empirical enrichment statistics for each pathway by performing 100,000 permutations. The nominal P-value returned by INRICH indicates the probability of observing the amount of overlap that exists between pathway gene sets and LD-independent associated intervals under the null hypothesis of no enrichment for associations at the specified association threshold [24]. Gene regions were defined as 20 kb up/downstream of the RefSeq transcription start/end sites for 17,529 autosomal genes using Human Genome Browser build hg18 [66], [67]. Next, pathway P-values were adjusted for multiple testing using resampling based second-step permutation [24].

### Gene-set Heritability Methods

The GCTA software package was used to generate all SNP based heritability estimates using linear mixed models [40]. This approach employs methods described in greater detail elsewhere [40], [68].
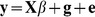
(1)


Where **y** is a vector of phenotype values, **b** is a vector of fixed effects of the overall mean, X is an incidence matrix for the fixed effects that relates these effects to individuals, **g** is a vector of random additive genetic effects based on aggregate SNP information, and **e** is a vector of random error effects. Phenotype variance estimates were estimated by the following formula:
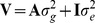
(2)


Additive genetic variance captured by SNPs is *σ* ^2^ *_g_*, and error variance is *σ* ^2^ *_e_*, **A** is the genetic relationship matrix estimated using SNPs, and **I** is an identity matrix. Variances were estimated using GCTA’s restricted maximum likelihood (REML) option, and then converted to heritability estimates [69].

We determine if gene-sets were enriched for their relative contribution to the heritability of BMI by examining if a greater proportion of the heritability was explained than expected based on the proportion of the genome represented [13], [70]. The binomial Z statistic method for comparing two proportions based on normal approximation was used to assess the degree of deviation [71]–[73].

## Supporting Information

Figure S1 **KEGG Fc epsilon RI signaling pathway intervals threshold top 10%.**
(DOC)

Figure S2 **KEGG Lysosome pathway top 0.5% intervals.**
(DOC)

Figure S3 **KEGG Lysosome pathway top 1% intervals.**
(DOC)

Figure S4 **KEGG Toll-like receptor-signaling pathway top 5% intervals.**
(DOC)

Figure S5 **The Reactome regulation of ornithine decarboxylase pathway top 5% intervals.**
(DOC)

Figure S6 **Reactome stabilization of P53 pathway top 5% intervals.**
(DOC)

Figure S7 **Signal Transduction ERK1/ERK2 MAPK top 5% intervals.**
(DOC)

Figure S8 **Replication Set Manhattan Plot.**
(DOC)

Figure S9 **Replication Set Quantile-Quantile Plot.**
(DOC)

Table S1 **INRICH Results for Discovery Set cutoff top 10%.**
(DOC)

Table S2 **INRICH Results for Discovery Set cutoff top 5%.**
(DOC)

Table S3 **INRICH Results for Discovery Set cutoff top 1%.**
(DOC)

Table S4 **INRICH Results for Discovery Set cutoff top 0.5%.**
(DOC)

Table S5 **INRICH Results for Replication Set cutoff top 10%.**
(DOC)

Table S6 **INRICH Results for Replication Set cutoff top 5%.**
(DOC)

Table S7 **INRICH Results for Replication Set cutoff top 1%.**
(DOC)

Table S8 **INRICH Results for Replication Set cutoff top 0.5%.**
(DOC)

Table S9 (XLS)

Table S10 **Excess of heritability beyond what is expected based on the proportion of the genome represented for nominally significant pathways at each threshold of significance examined.**
(DOC)

Table S11 **Replication set population statistics.**
(DOC)
