# Supplementary figures and images for: Whole-Genome Pathway Analysis on 132,497 Individuals Identifies Novel Gene-Sets Associated with Body Mass Index
Source: PLoS One. 2014 Jan 31;9(1):e78546. doi: 10.1371/journal.pone.0078546 (PMC3908858; doi:10.1371/journal.pone.0078546)

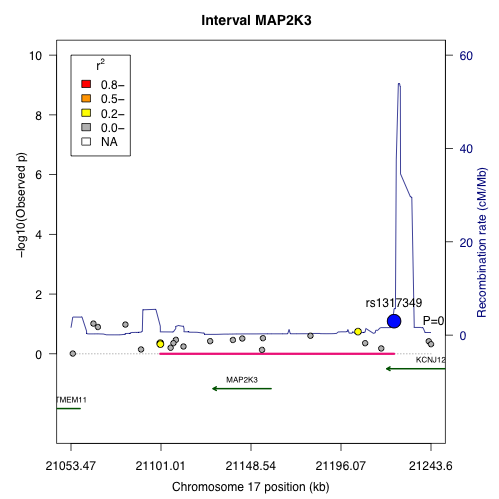


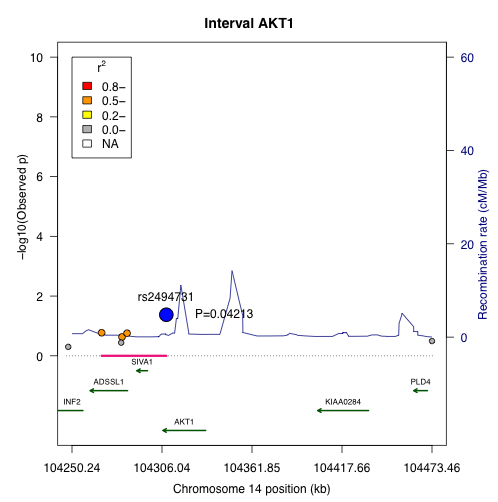

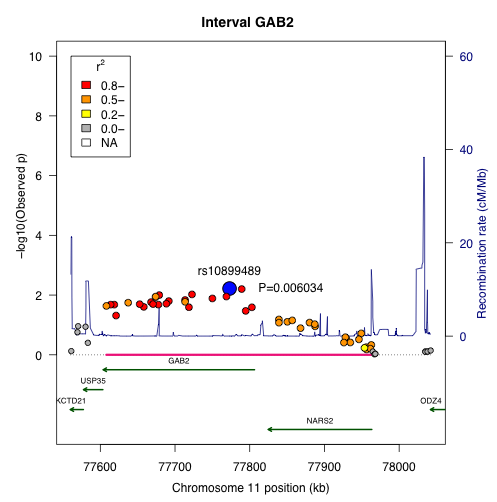

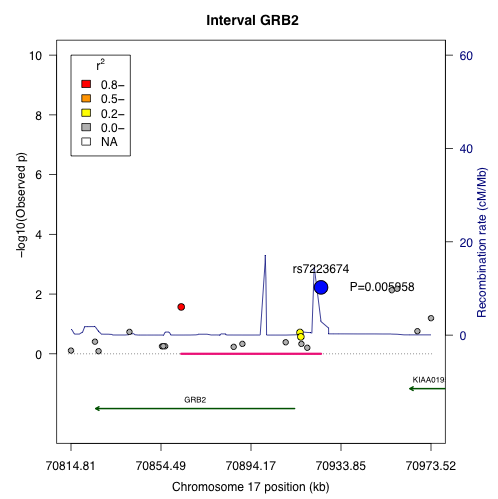

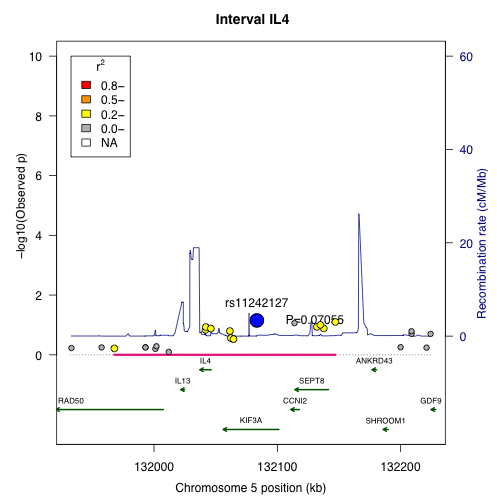

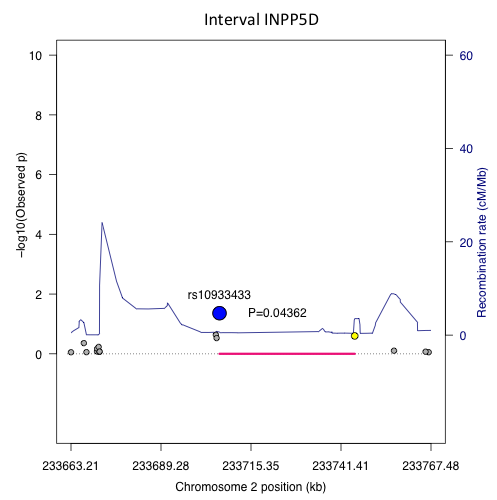

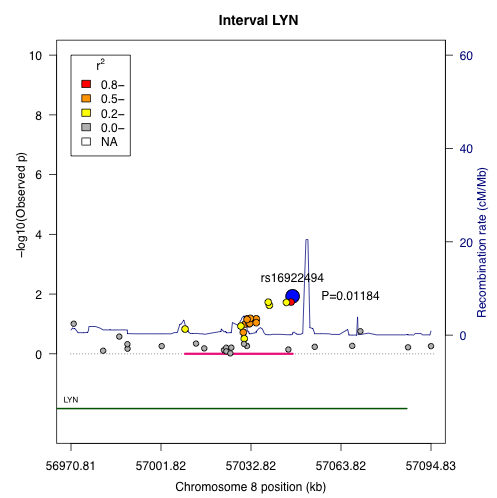

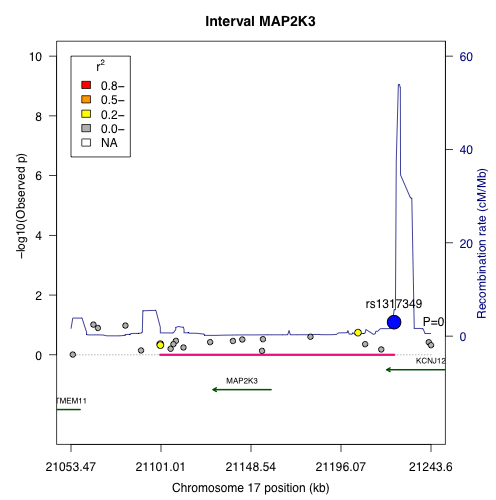

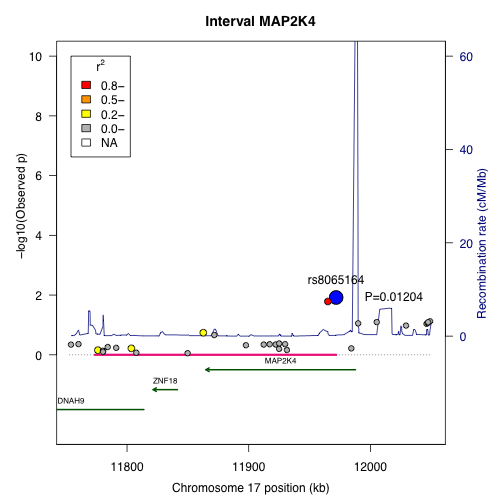

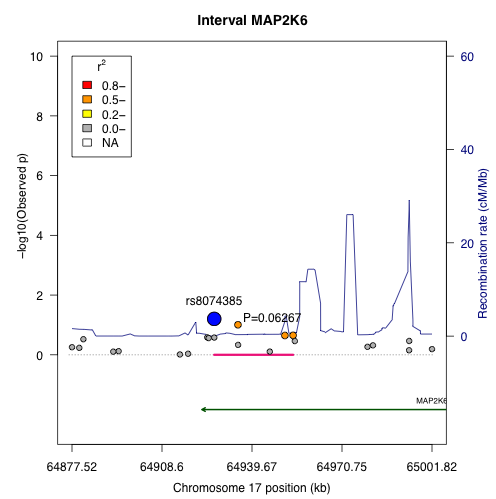

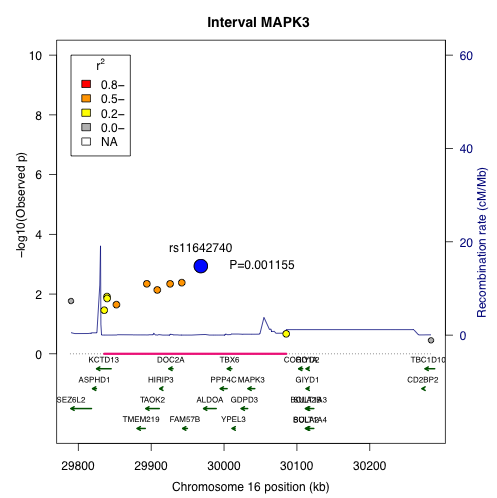

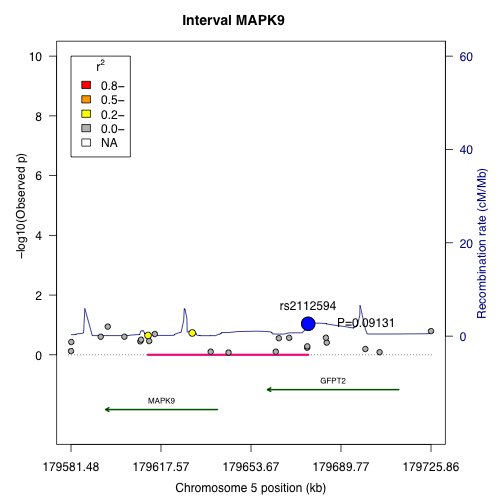

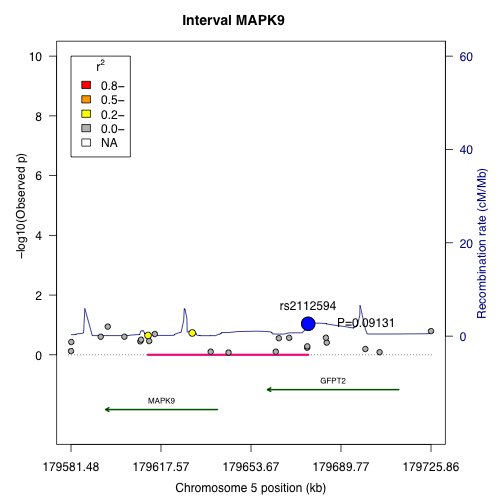

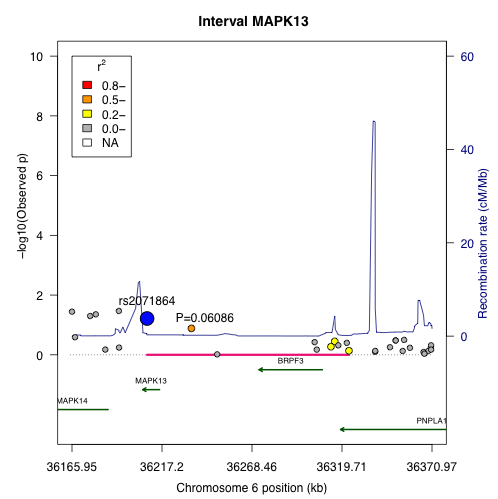

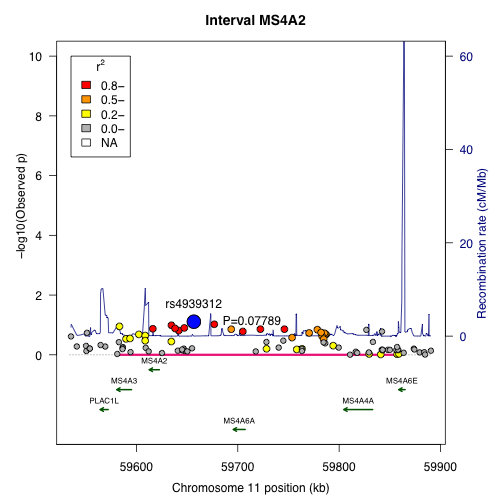

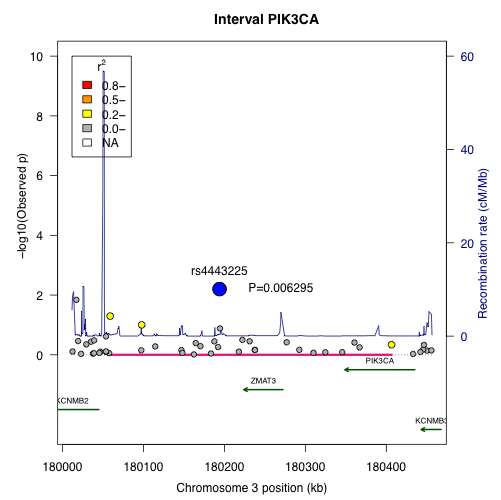

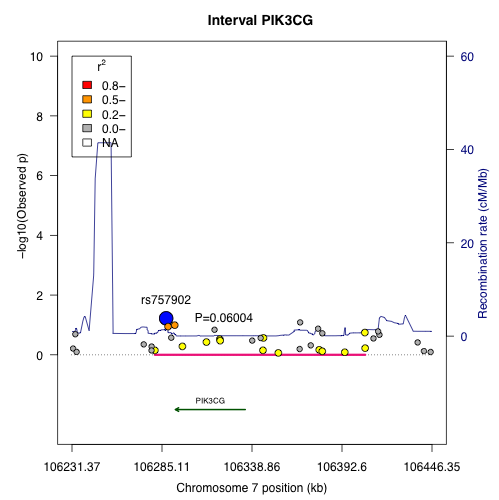

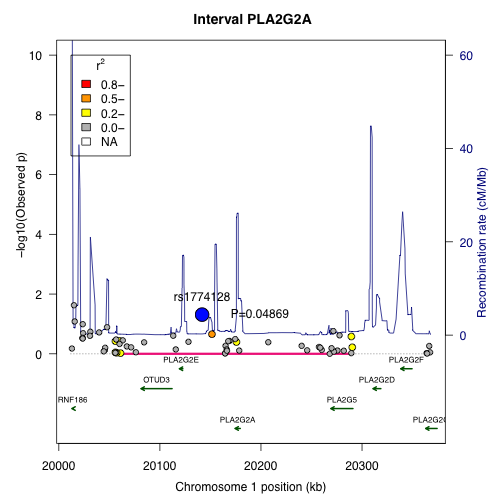

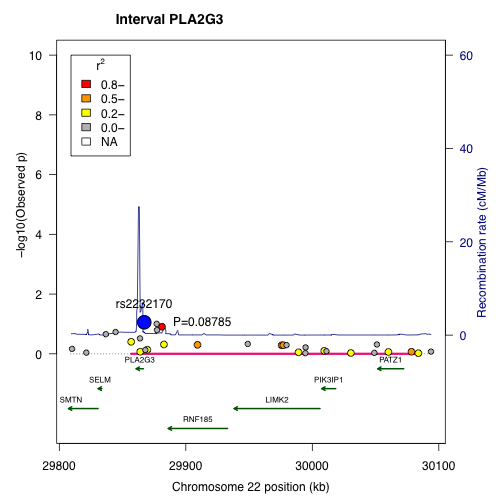

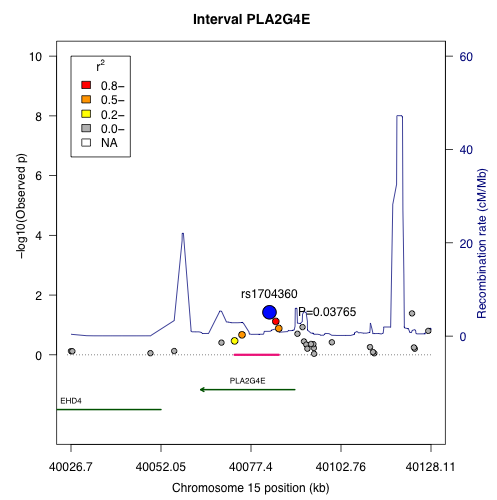

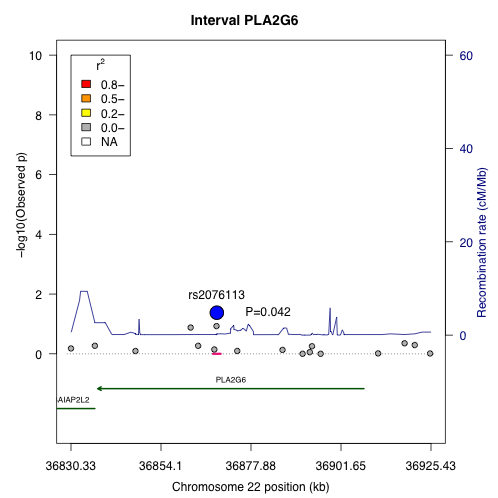

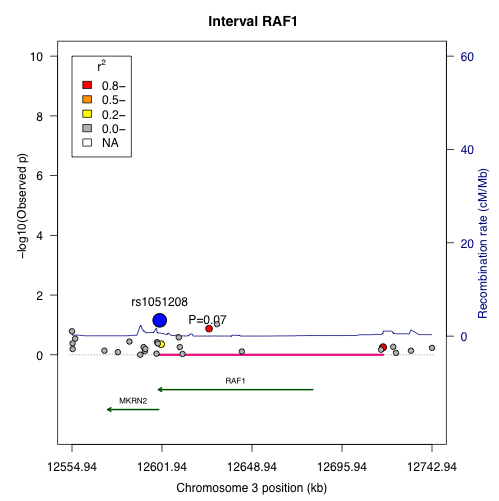

Supplement: Figure S1 — KEGG Fc epsilon RI signaling pathway intervals threshold top 10%. (DOC) [file pone.0078546.s001.doc]

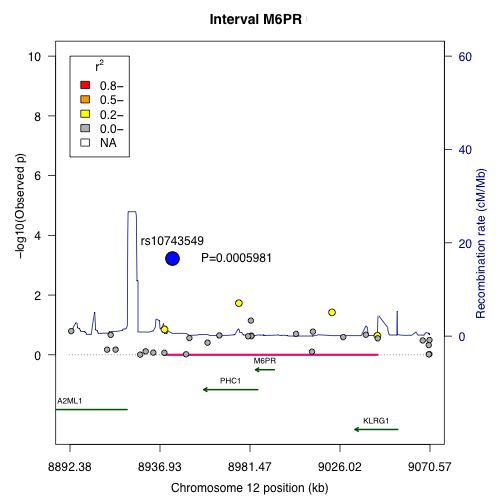


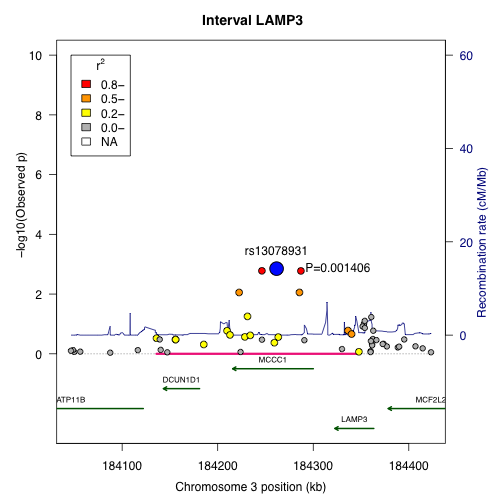

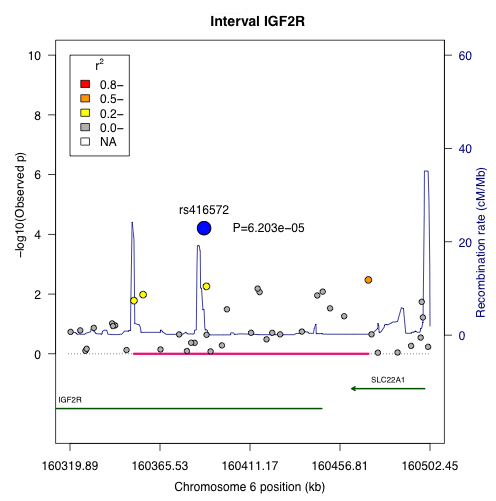

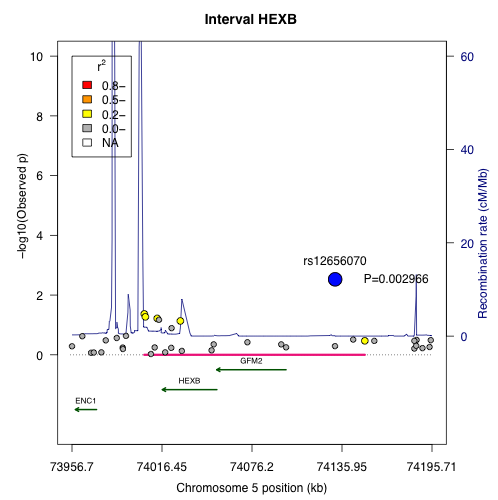

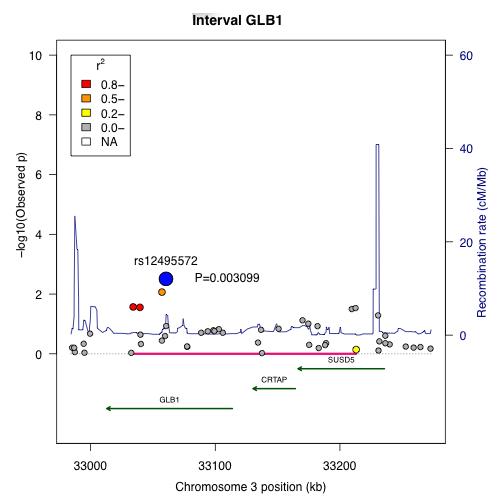

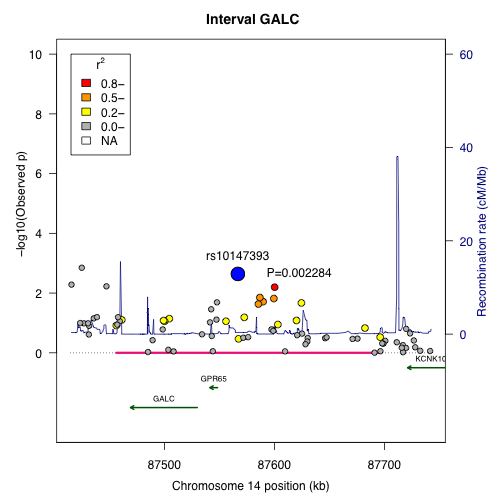

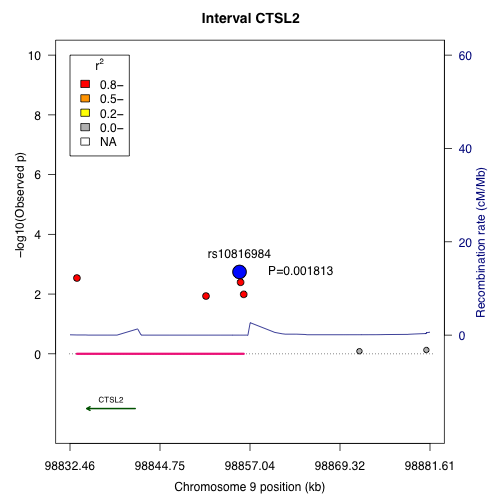

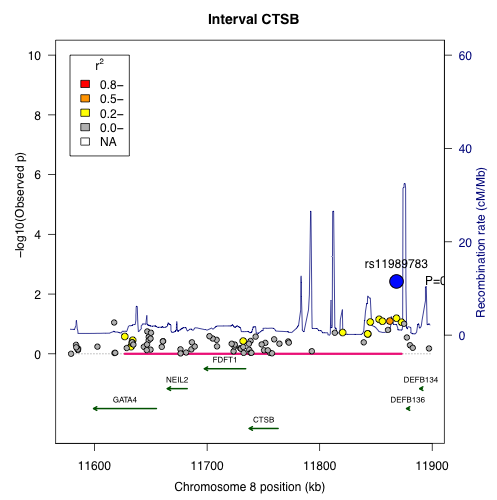

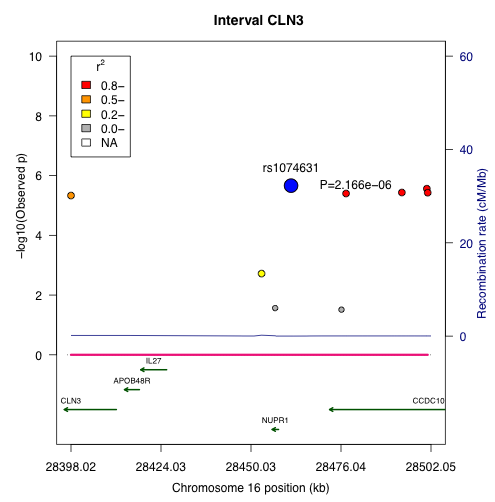

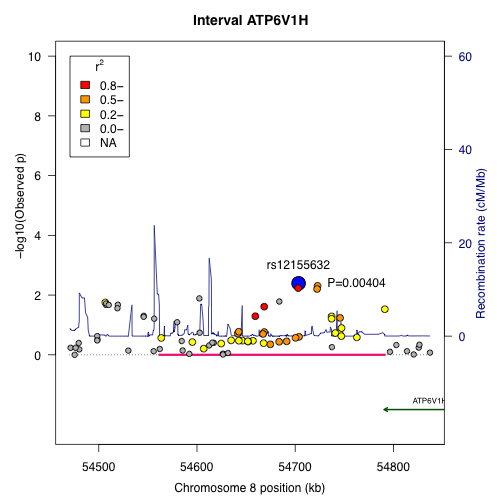

Supplement: Figure S2 — KEGG Lysosome pathway top 0.5% intervals. (DOC) [file pone.0078546.s002.doc]

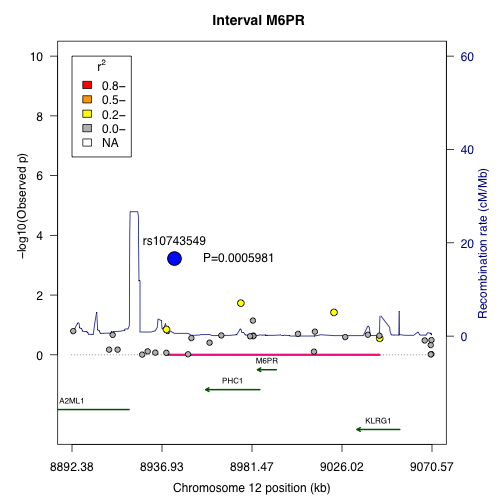


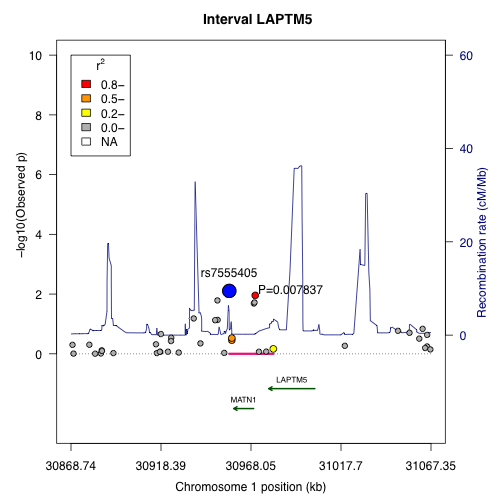

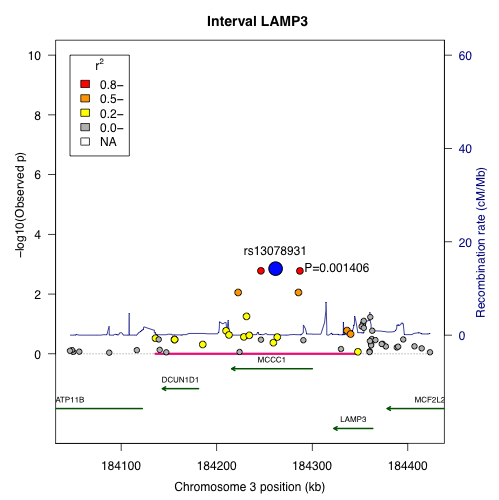

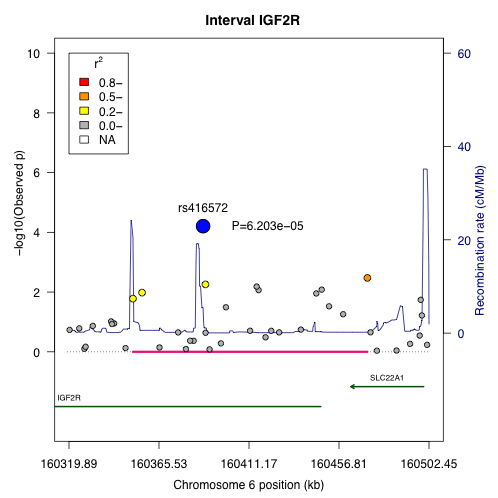

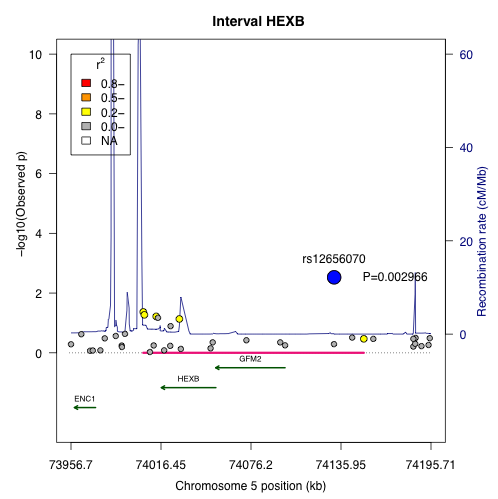

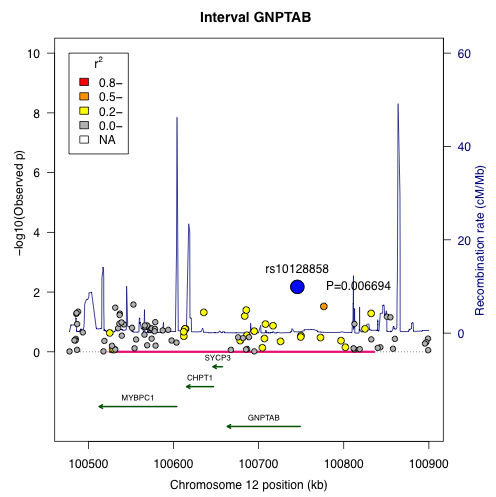

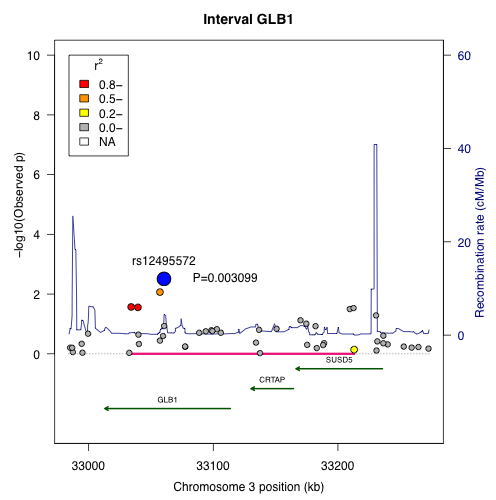

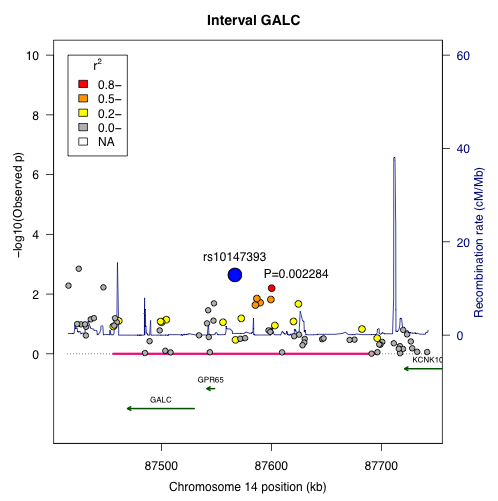

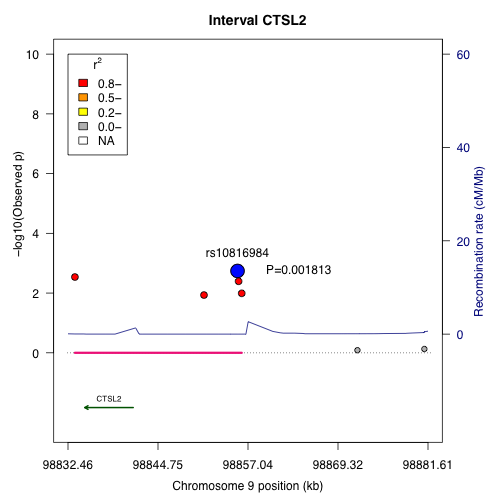

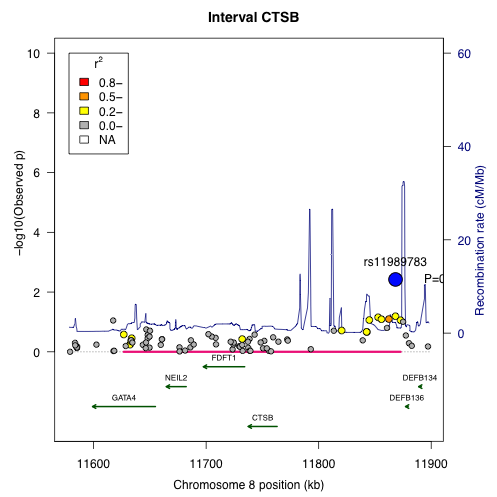

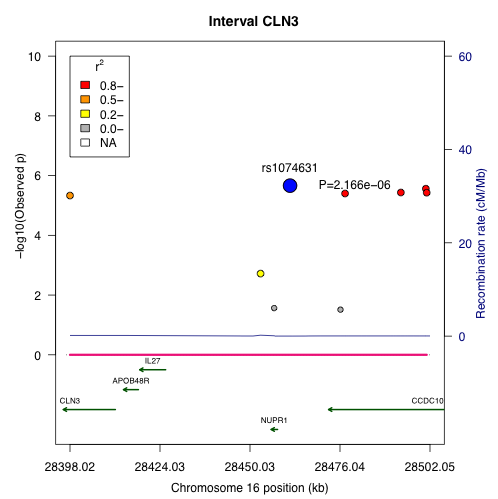

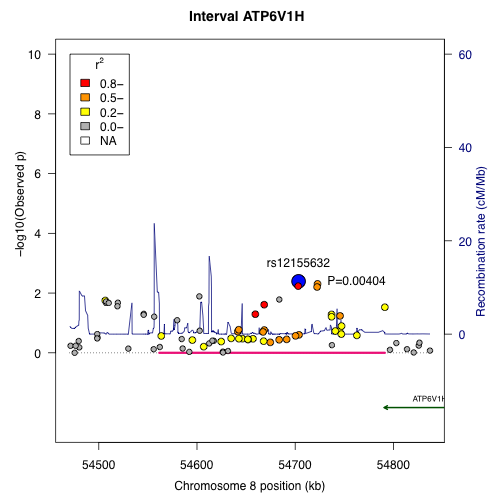

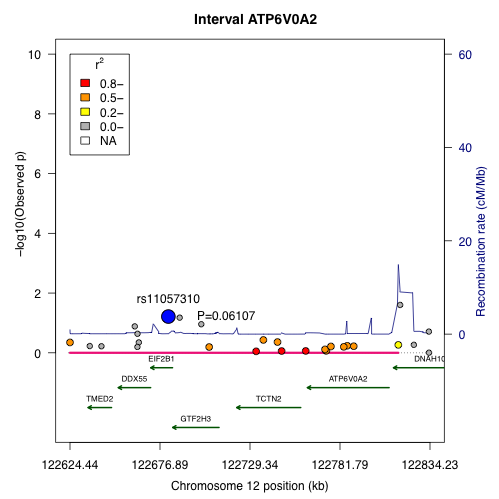

Supplement: Figure S3 — KEGG Lysosome pathway top 1% intervals. (DOC) [file pone.0078546.s003.doc]

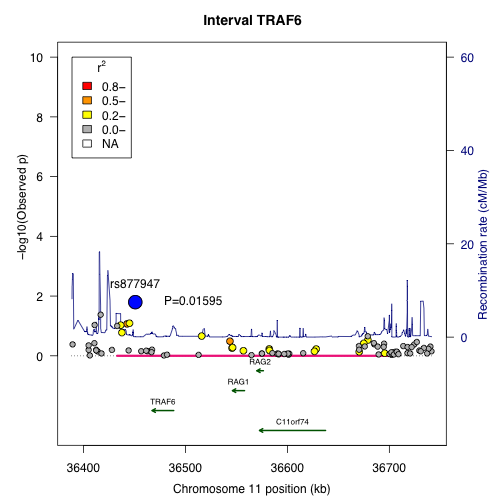


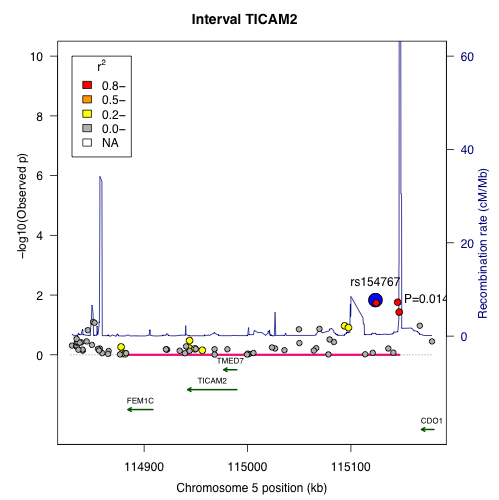

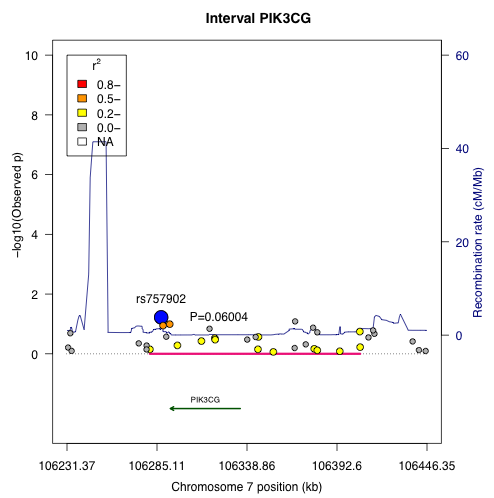

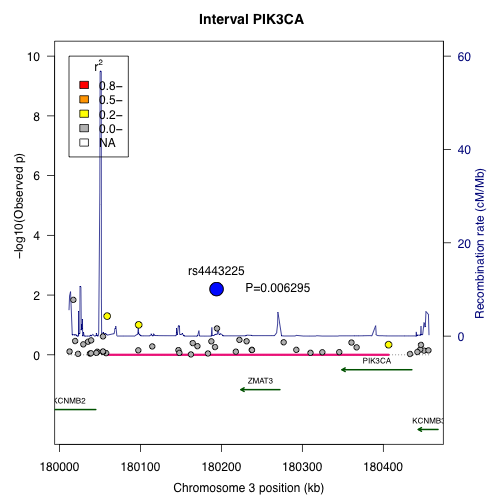

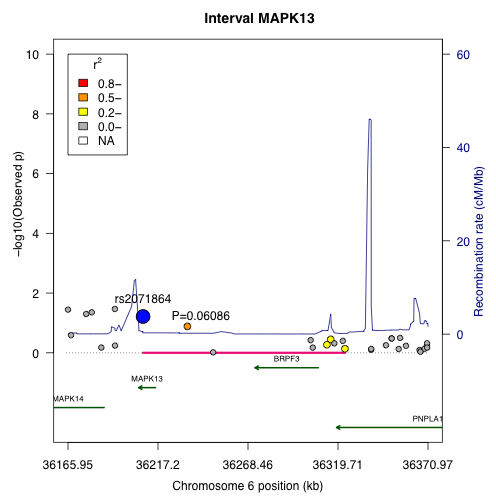

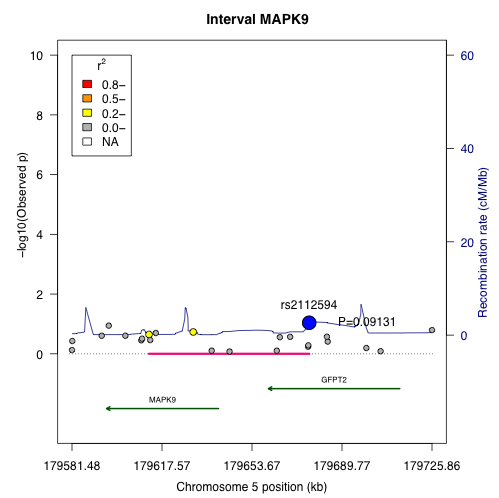

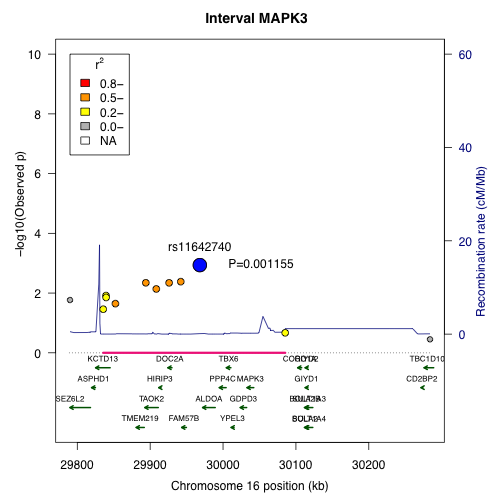

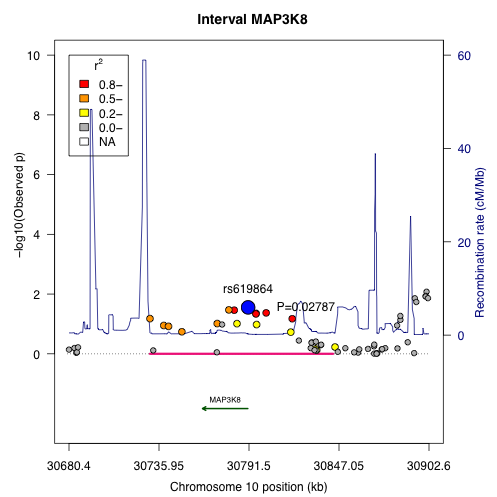

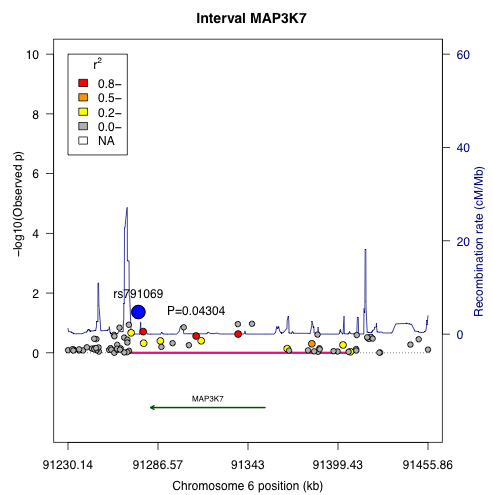

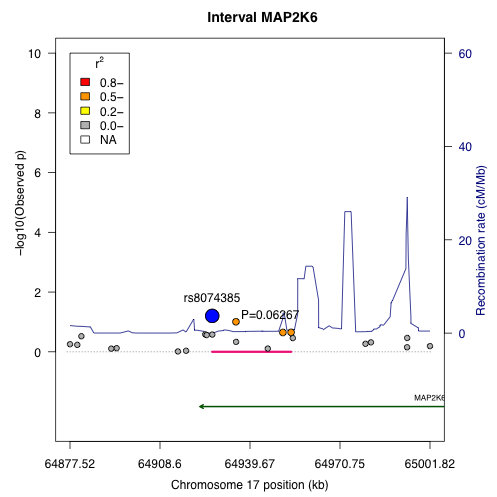

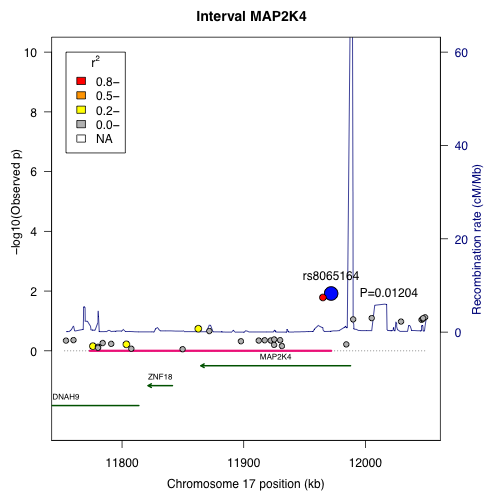

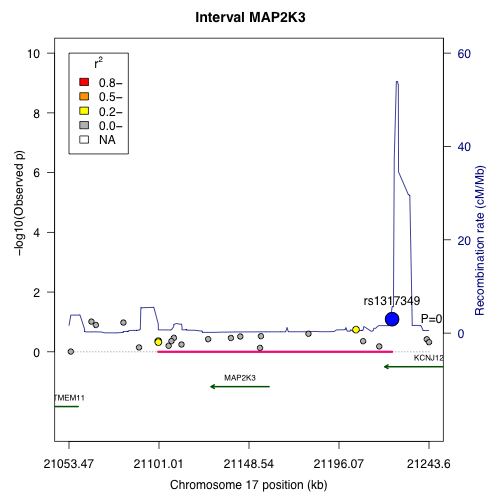

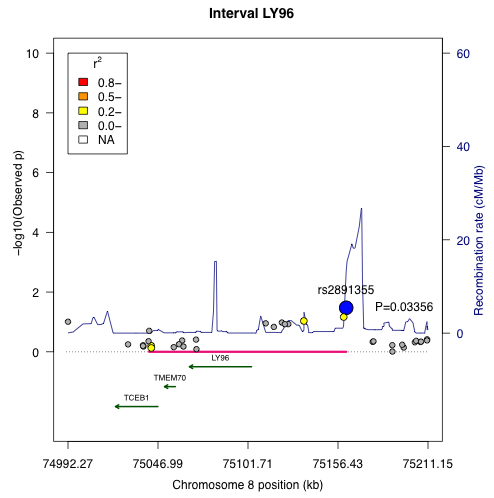

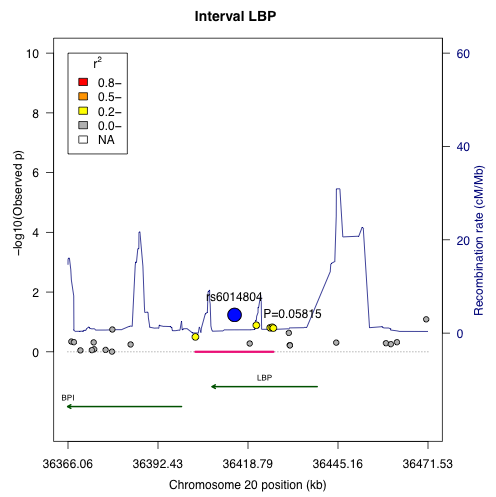

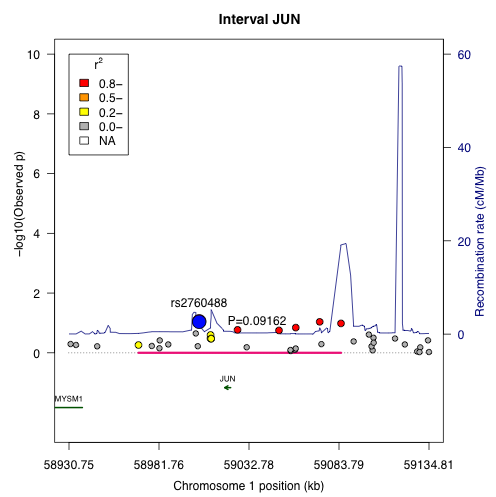

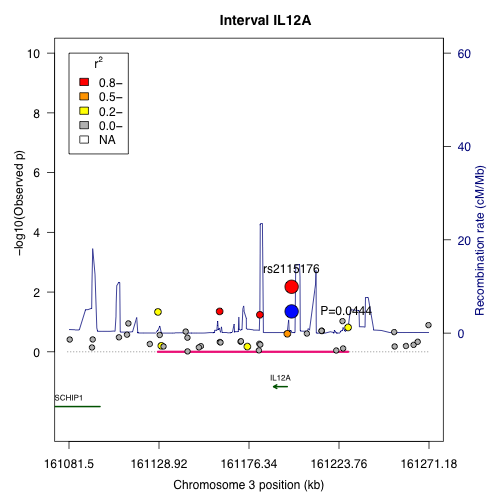

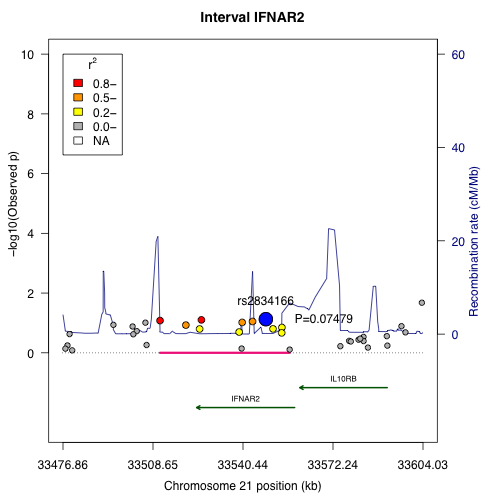

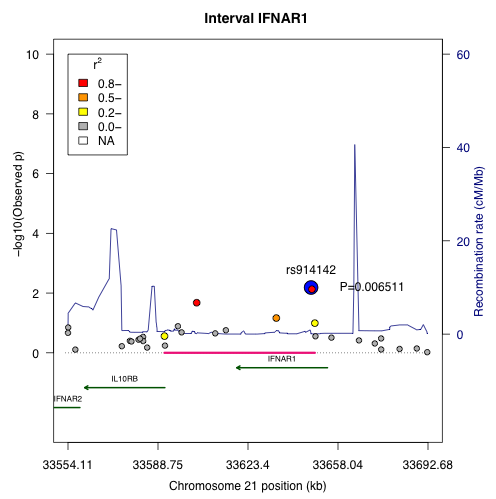

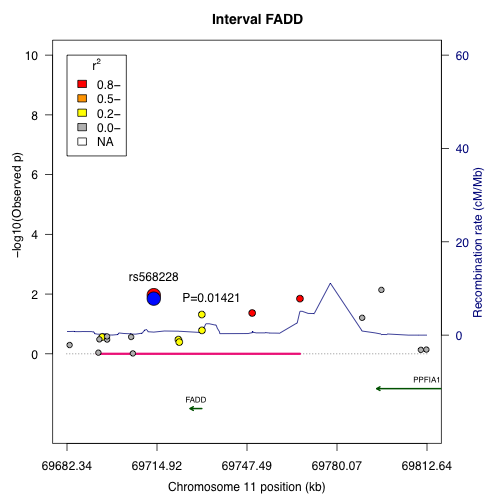

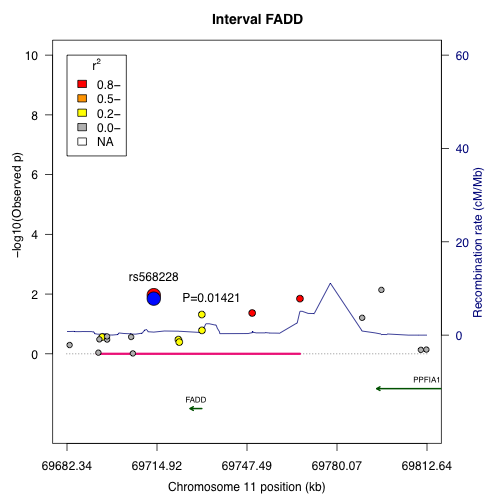

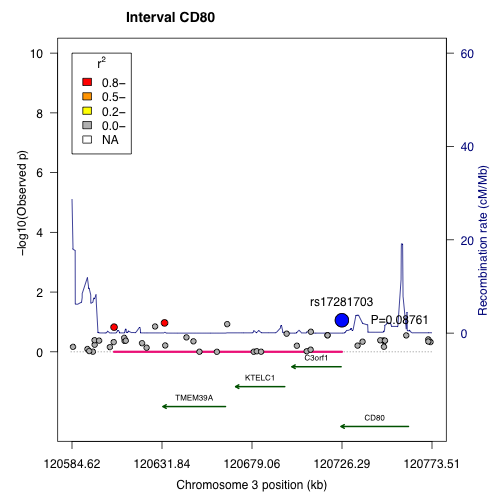

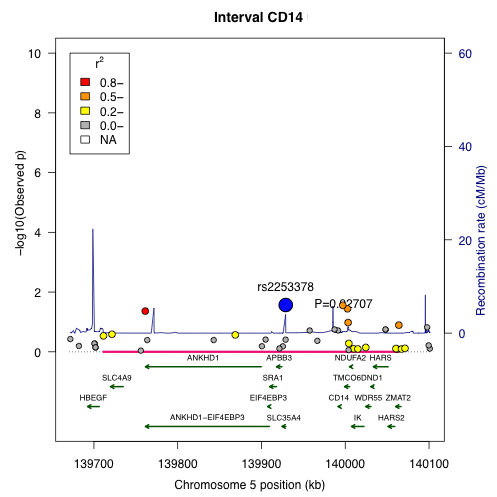

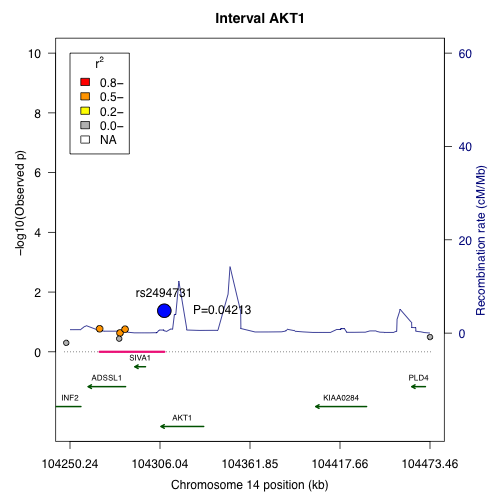

Supplement: Figure S4 — KEGG Toll-like receptor-signaling pathway top 5% intervals. (DOC) [file pone.0078546.s004.doc]

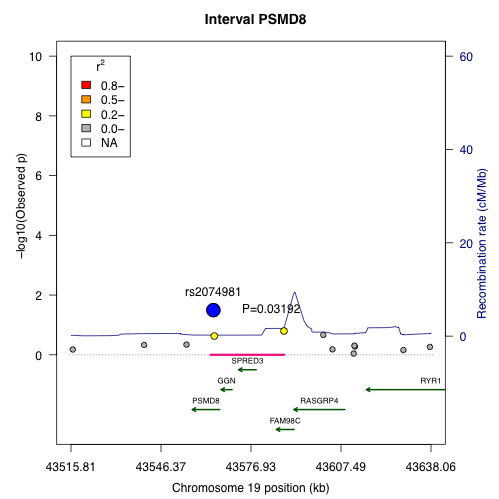


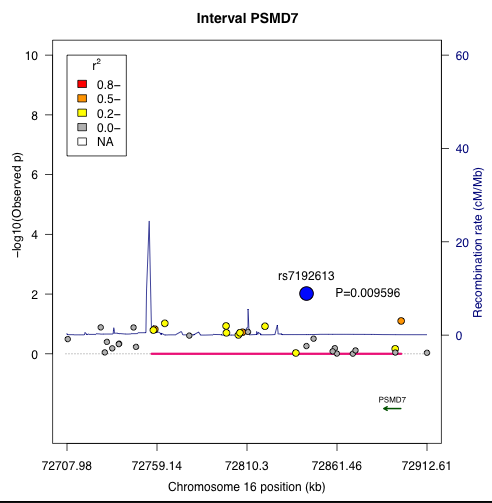

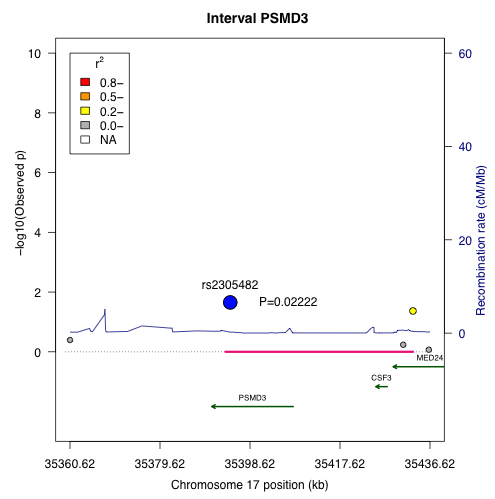

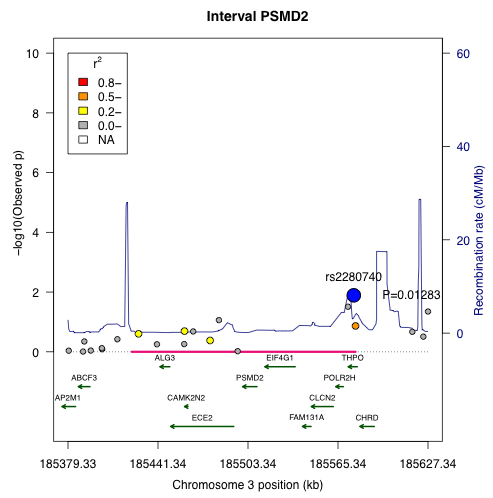

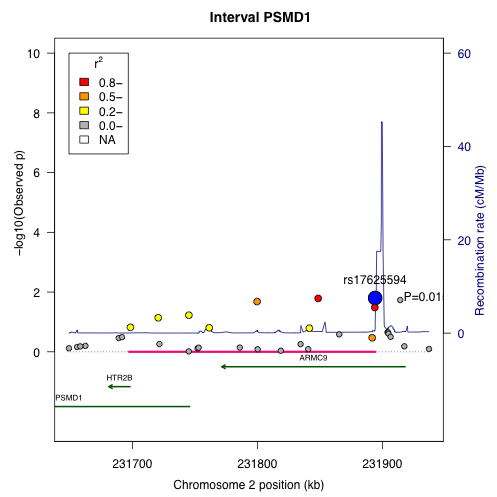

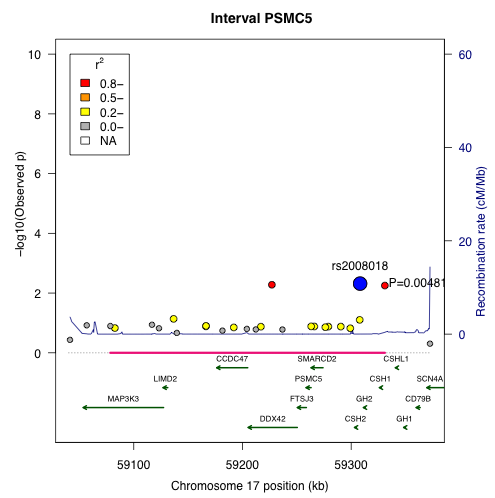

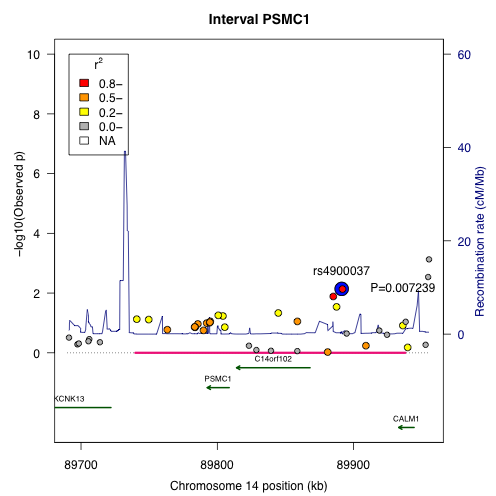

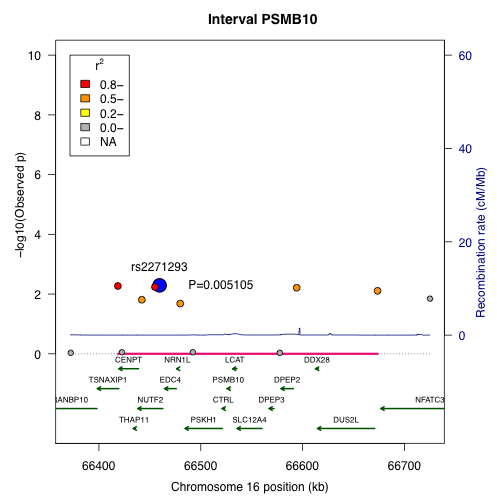

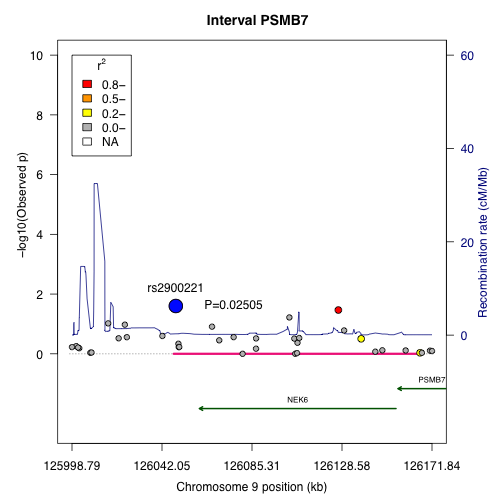

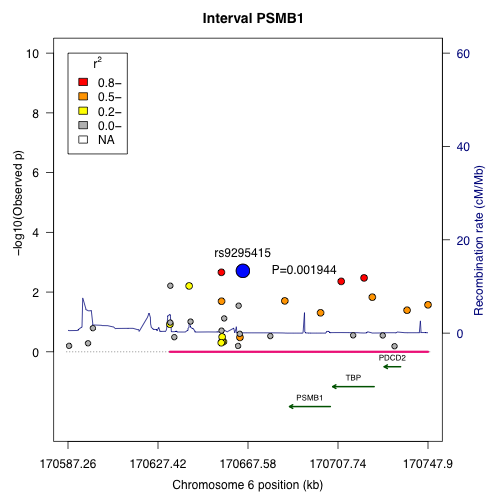

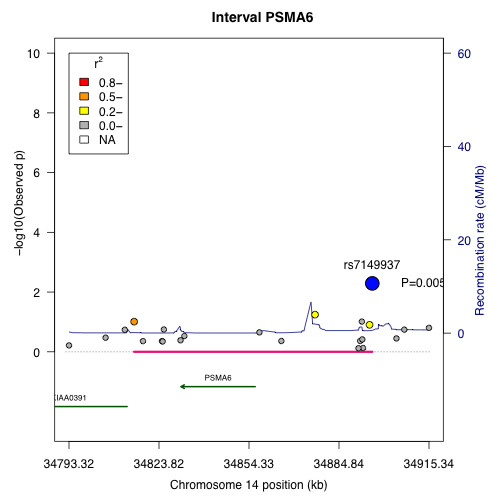

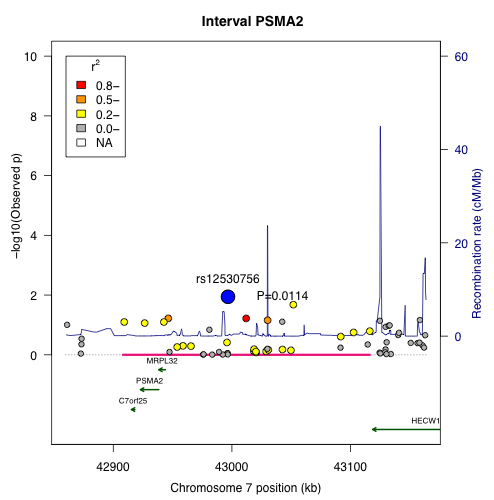

Supplement: Figure S5 — The Reactome regulation of ornithine decarboxylase pathway top 5% intervals. (DOC) [file pone.0078546.s005.doc]

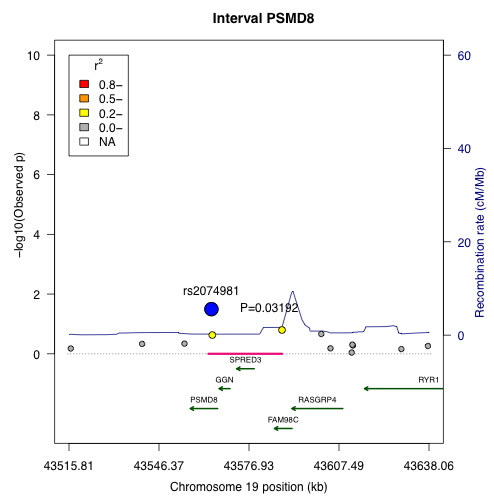


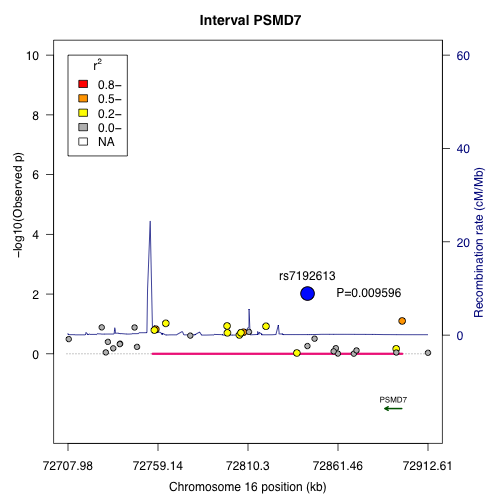

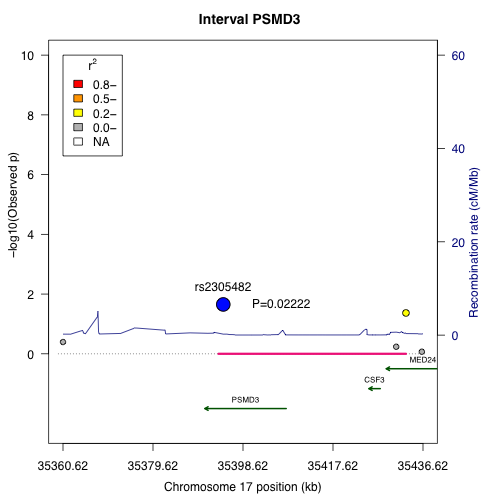

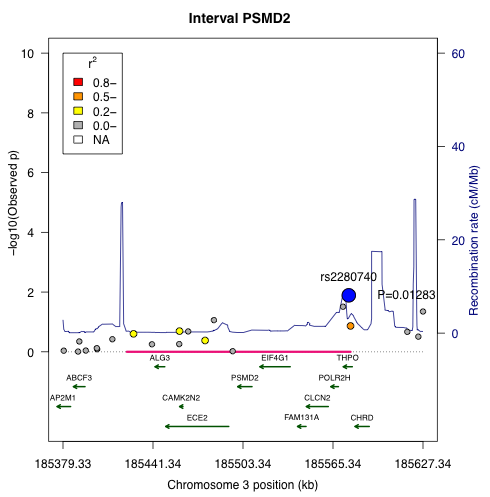

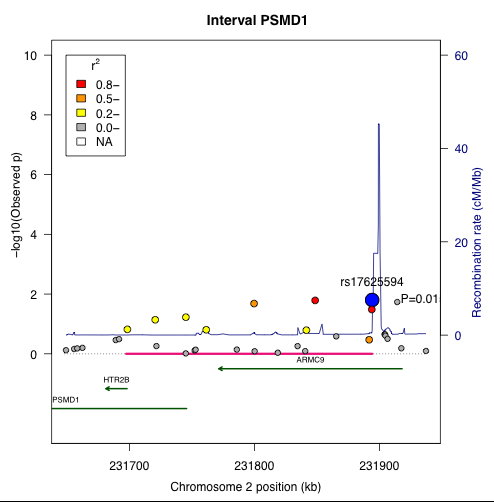

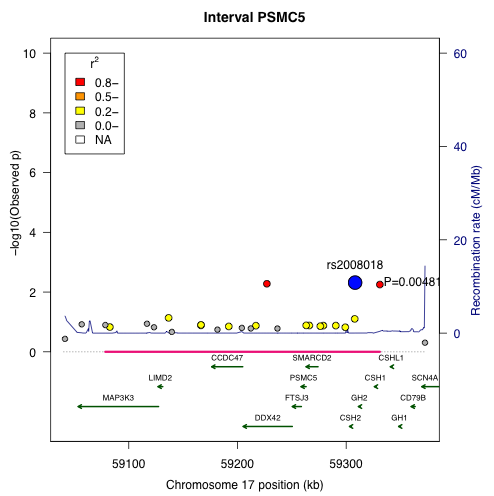

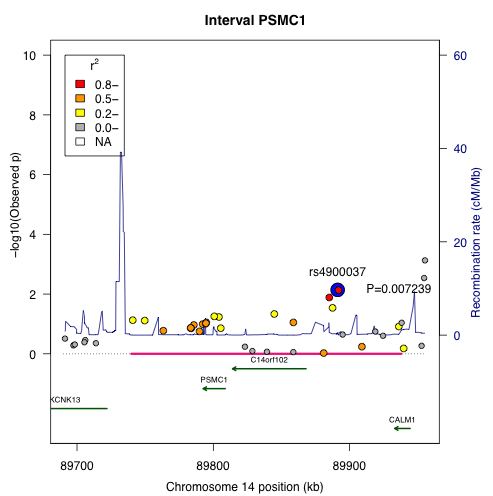

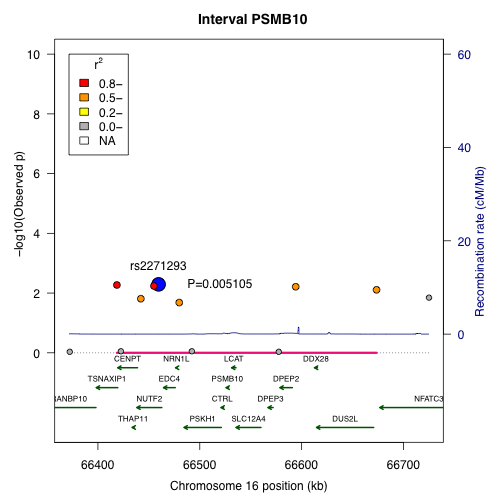

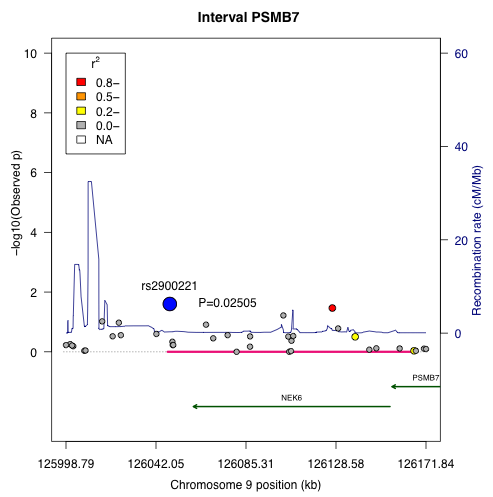

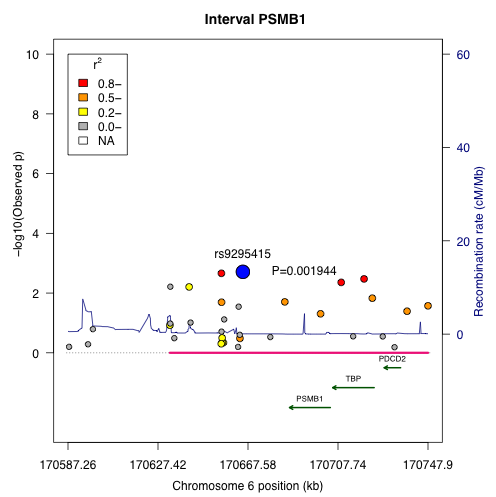

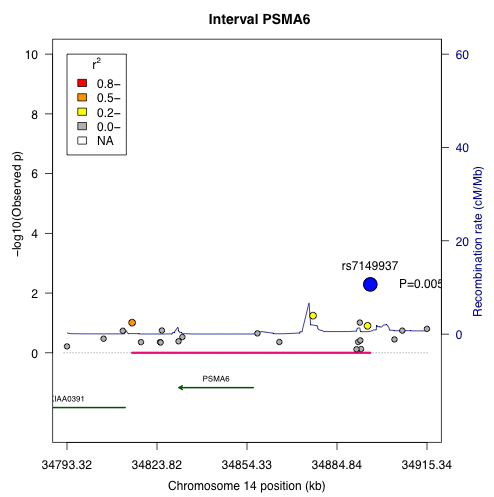

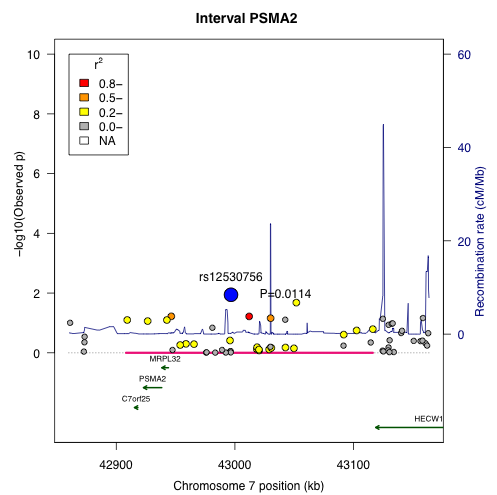

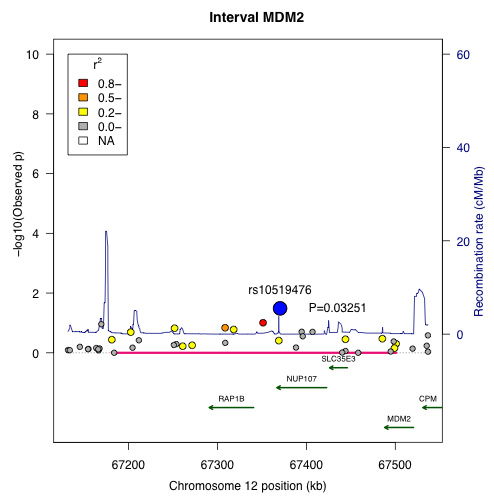

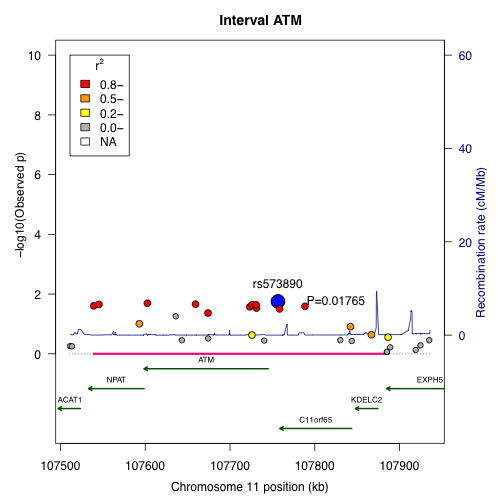

Supplement: Figure S6 — Reactome stabilization of P53 pathway top 5% intervals. (DOC) [file pone.0078546.s006.doc]

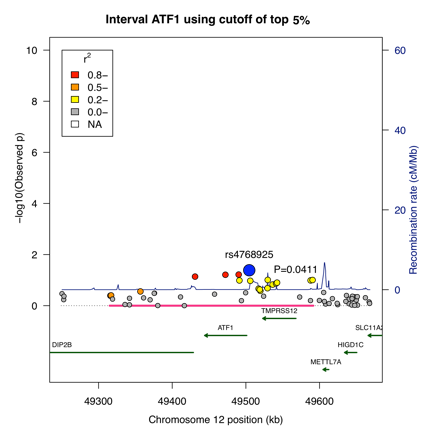

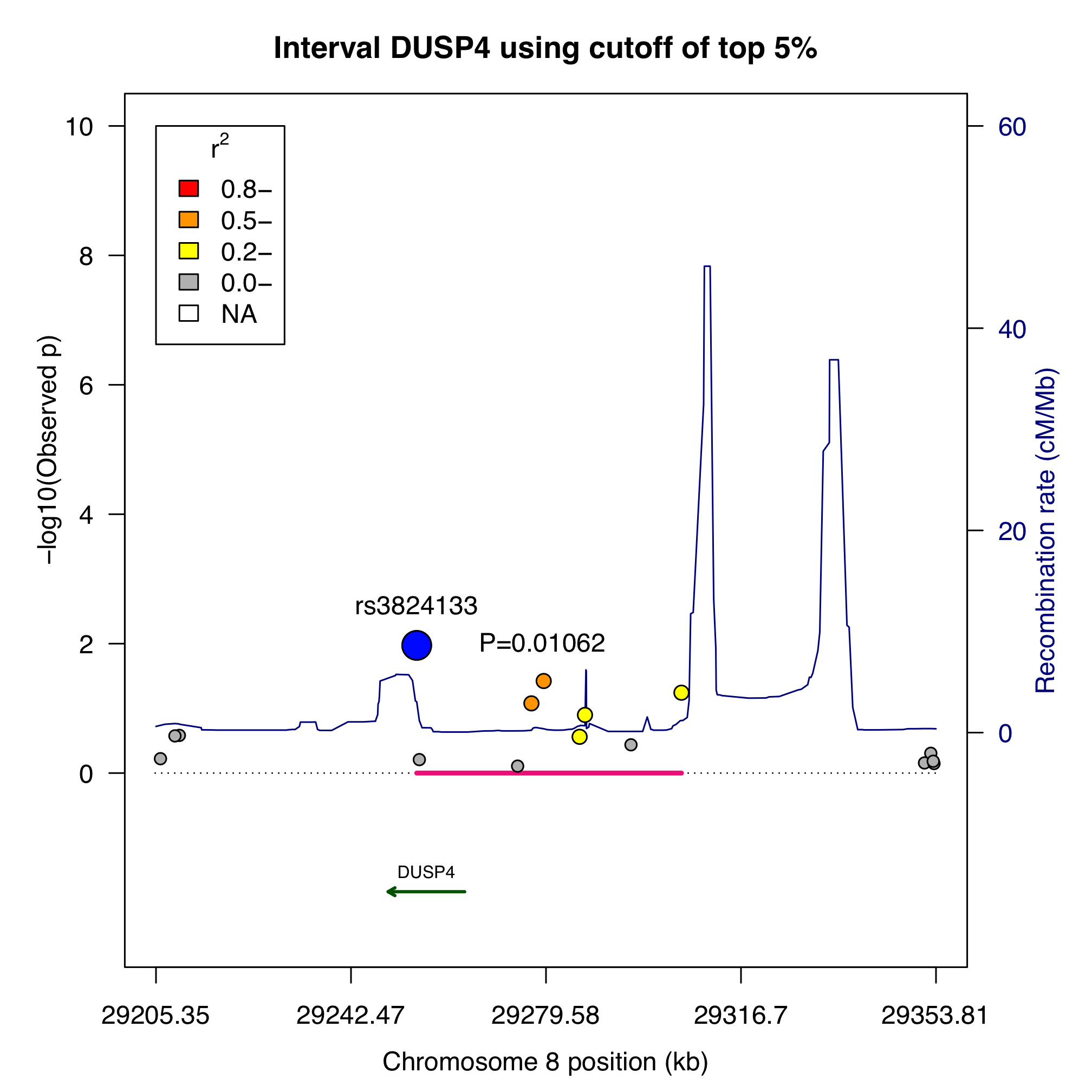

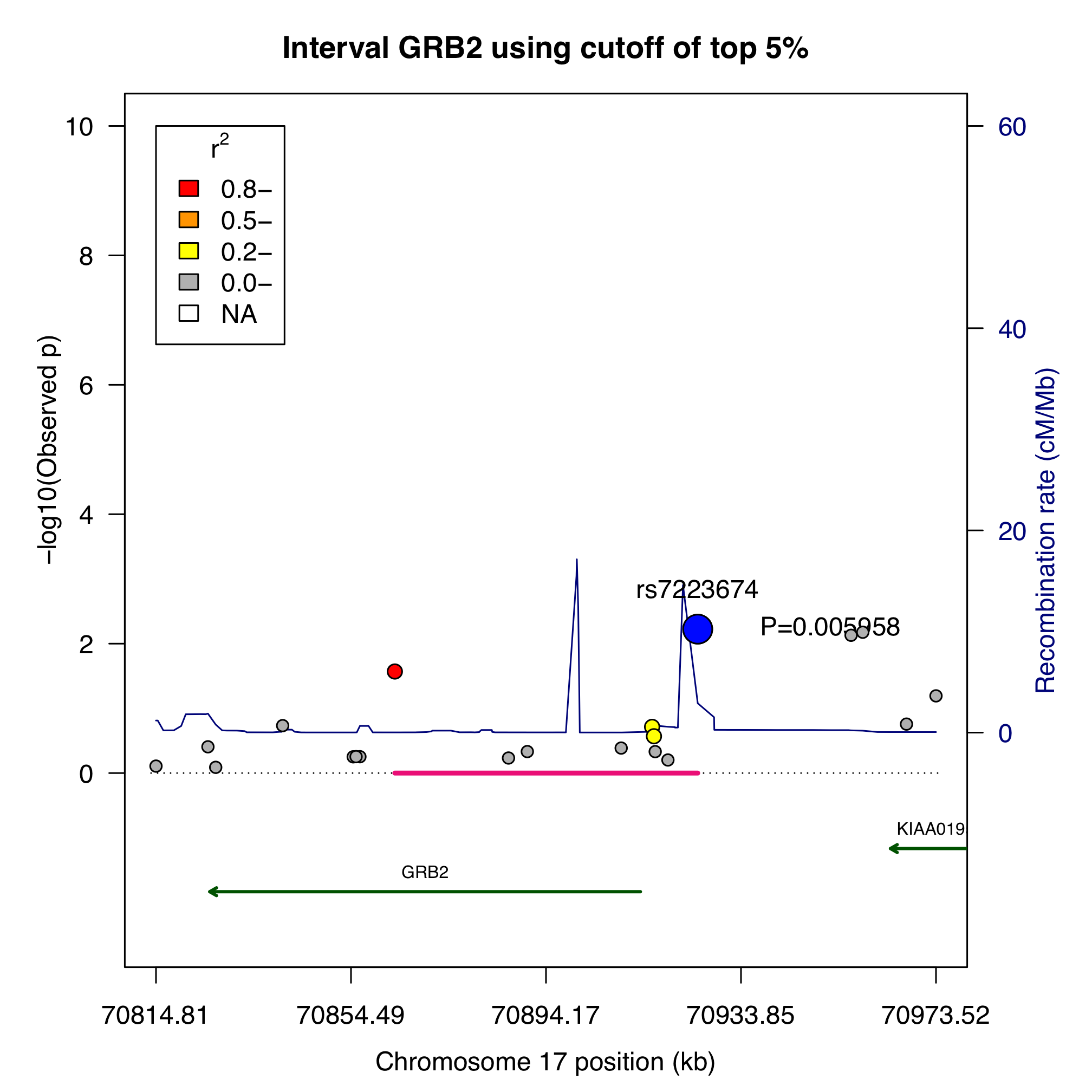

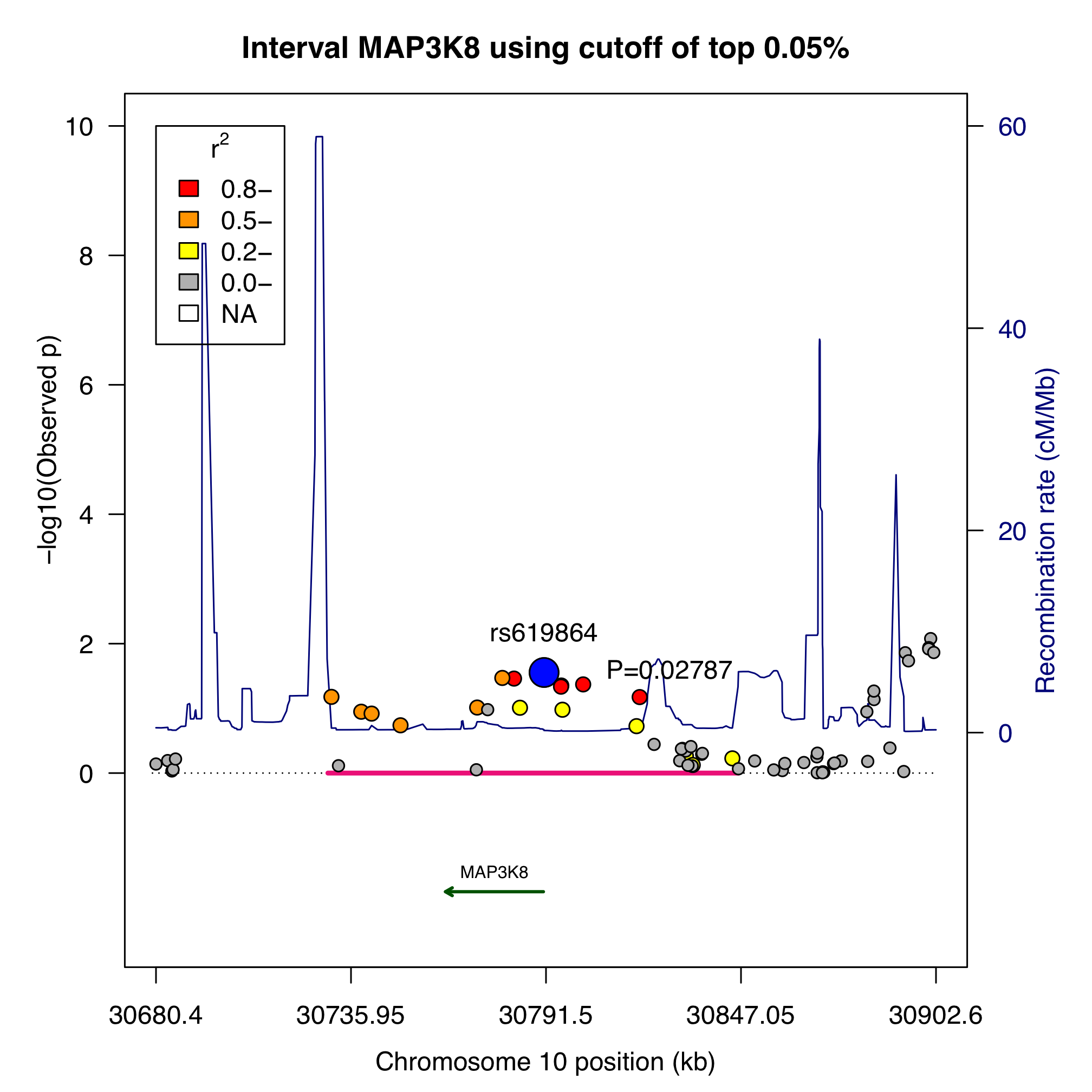

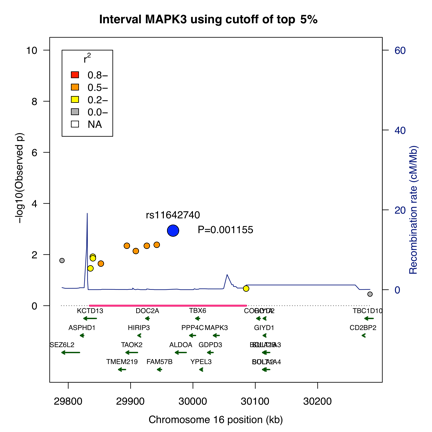

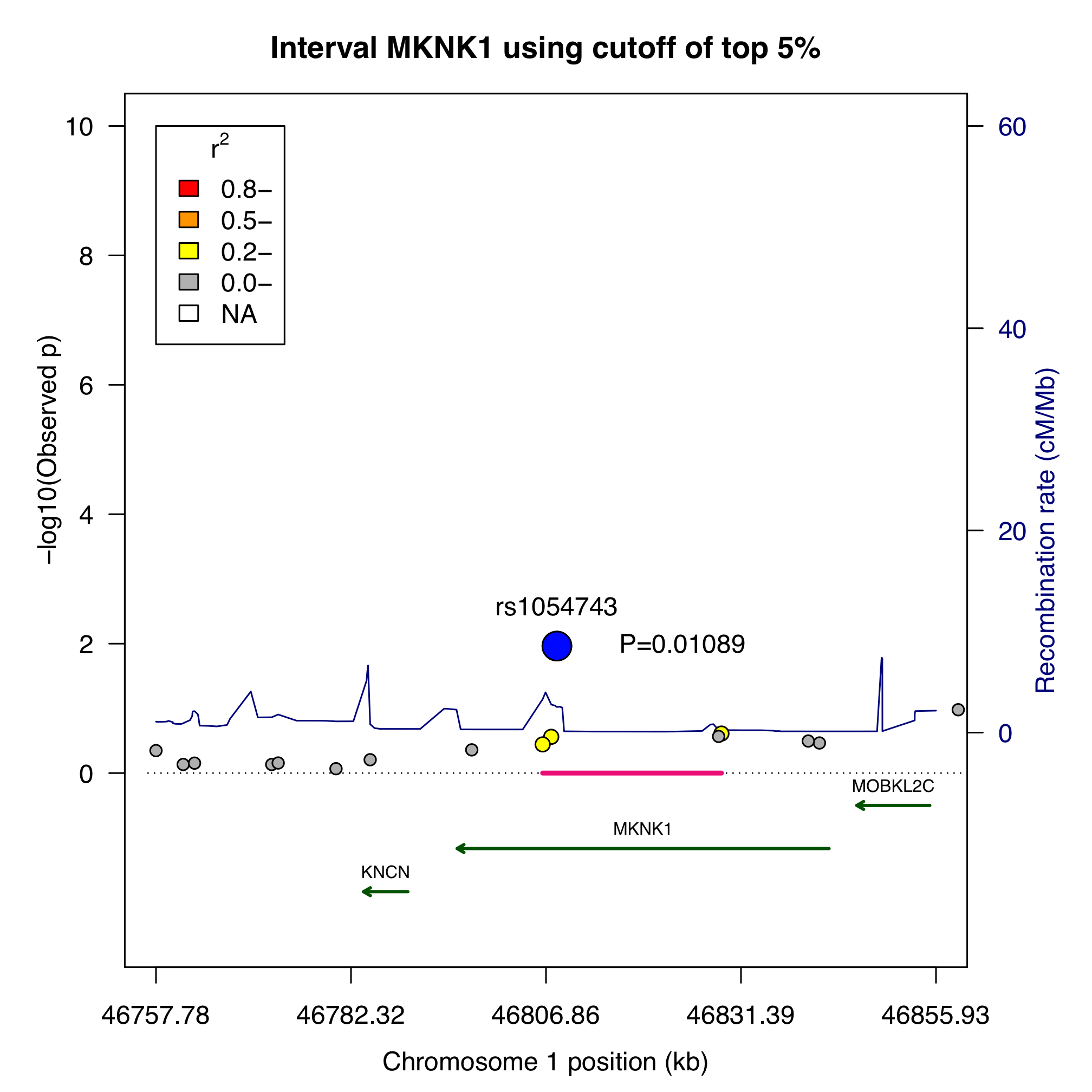

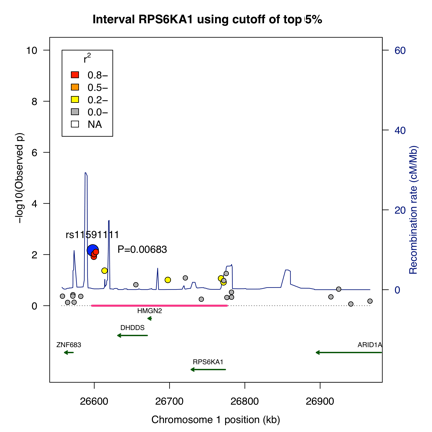

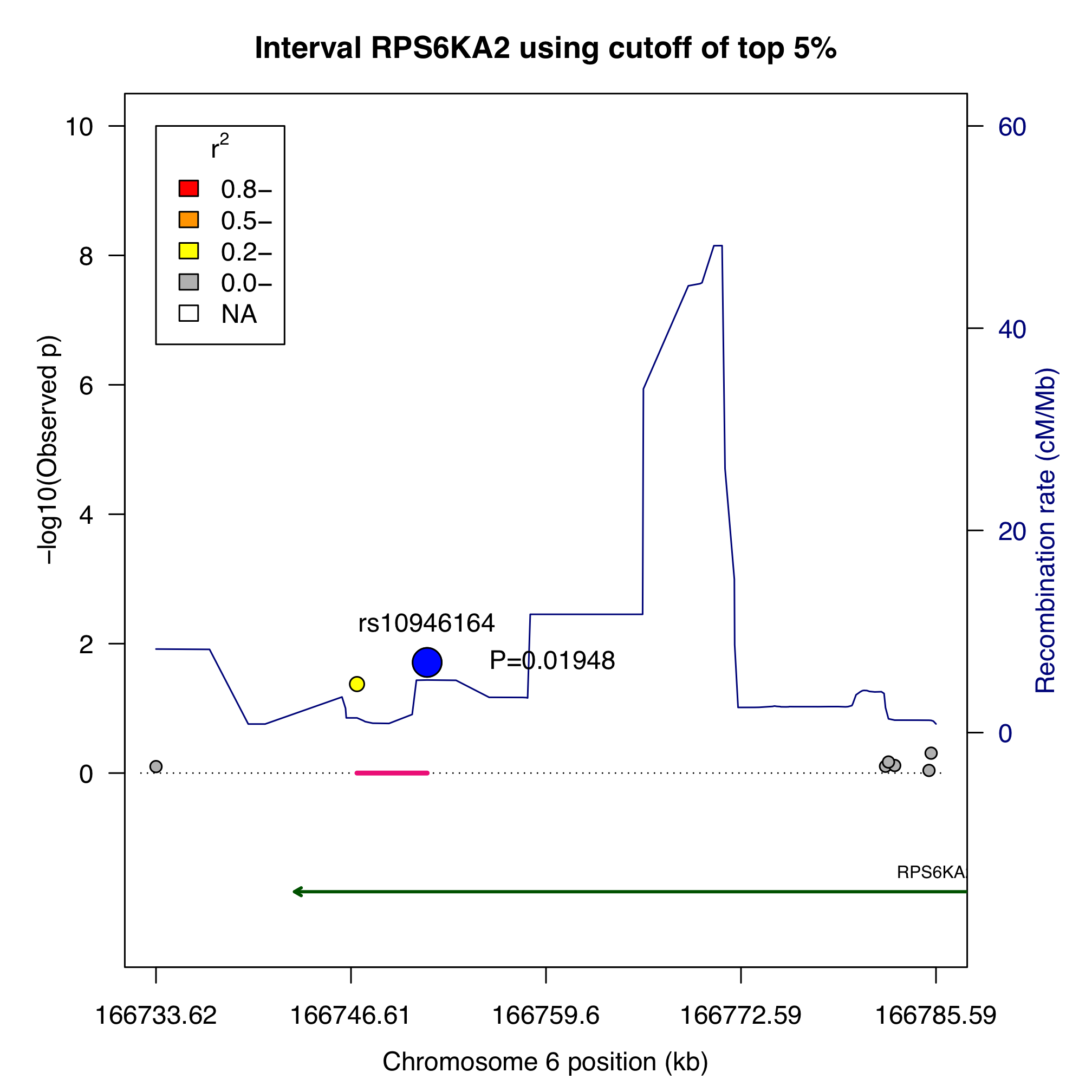

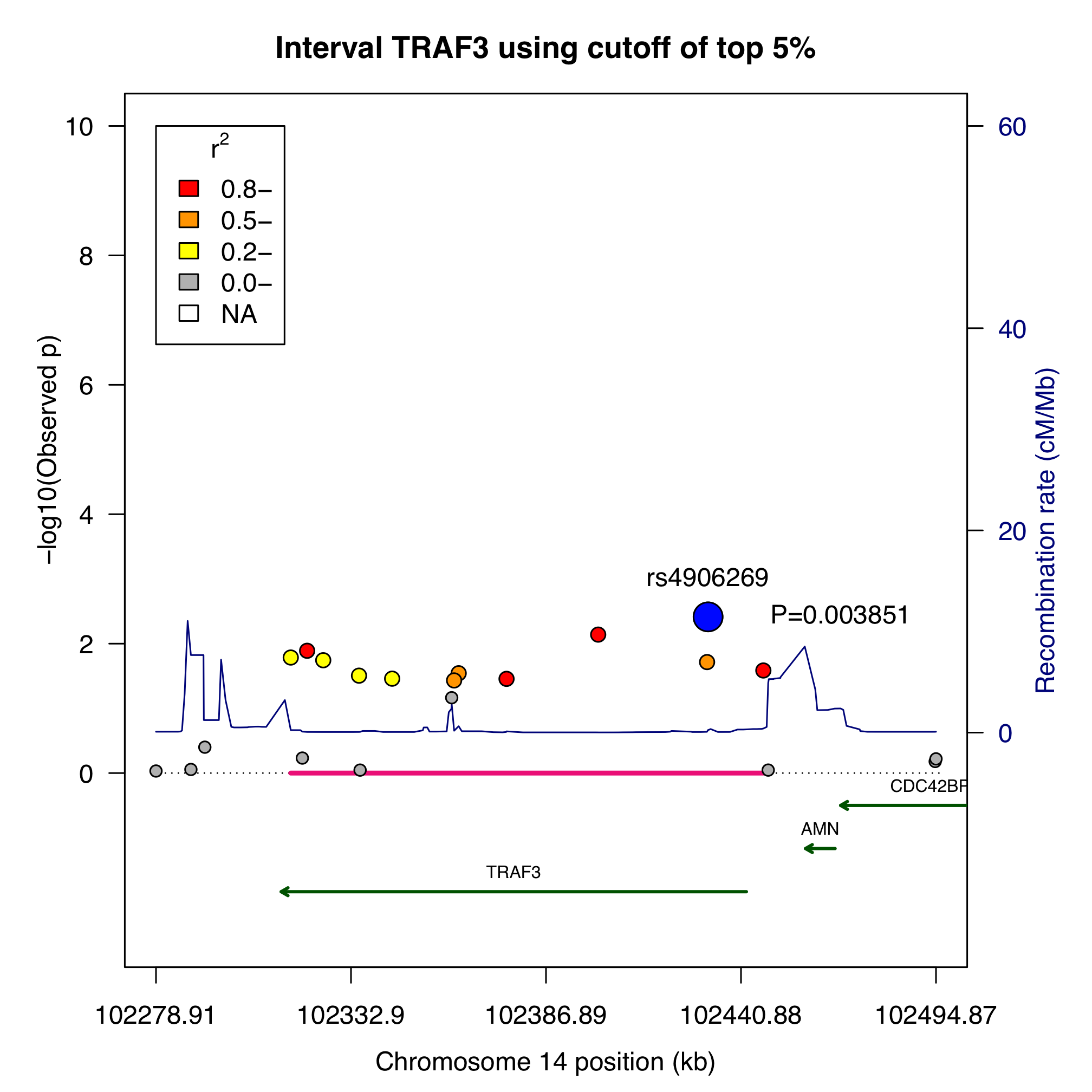

Supplement: Figure S7 — Signal Transduction ERK1/ERK2 MAPK top 5% intervals. (DOC) [file pone.0078546.s007.doc]

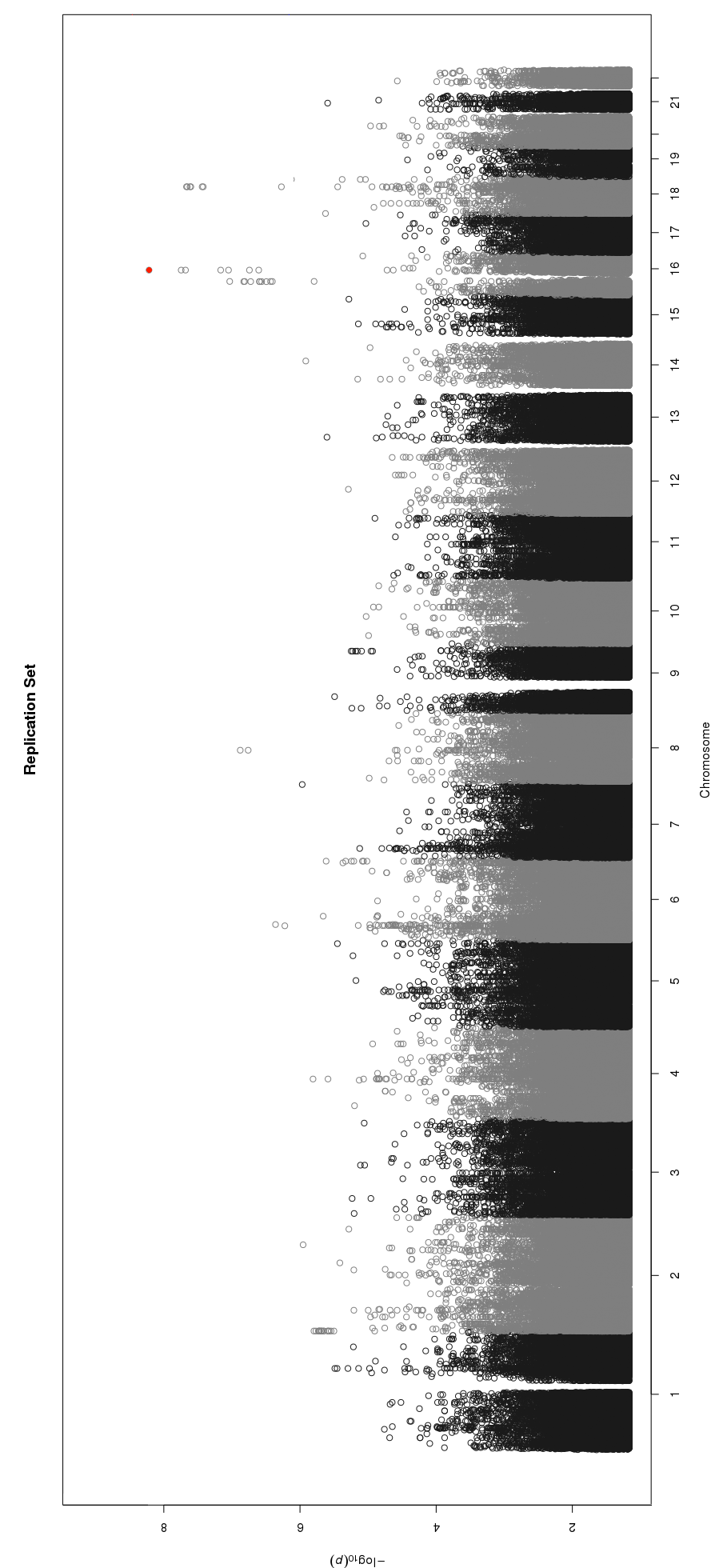

Supplement: Figure S8 — Replication Set Manhattan Plot. (DOC) [file pone.0078546.s008.doc]

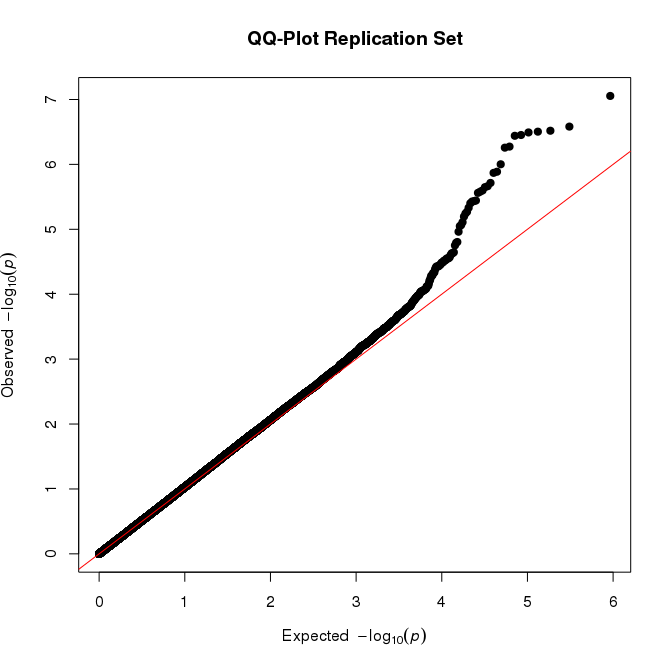

Supplement: Figure S9 — Replication Set Quantile-Quantile Plot. (DOC) [file pone.0078546.s009.doc]
