# Supplementary material for: Whole-Genome Pathway Analysis on 132,497 Individuals Identifies Novel Gene-Sets Associated with Body Mass Index
Source: PLoS One. 2014 Jan 31;9(1):e78546. doi: 10.1371/journal.pone.0078546 (PMC3908858; doi:10.1371/journal.pone.0078546)
Supplement: Table S1 — INRICH Results for Discovery Set cutoff top 10%. (DOC) [file pone.0078546.s010.doc]

Table S1. Discovery INRICH results

INRICH Results for Discovery Set cutoff top 10%

| Target_Size | Int_No | Empirical_P | Corrected_P | Pathway |
| --- | --- | --- | --- | --- |
| 86 | 52 | 4.00E-05 | 0.0173965 | BIOCARTA_MAPK_PATHWAY |
| 79 | 46 | 0.000119999 | 0.0353929 | REACTOME_G2_M_TRANSITION |
| 155 | 77 | 0.000199998 | 0.0505899 | REACTOME_MITOTIC_M_M_G1_PHASES |
| 67 | 40 | 0.000329997 | 0.0803839 | REACTOME_CENTROSOME_MATURATION |
| 59 | 37 | 0.000389996 | 0.0917816 | REACTOME_LOSS_OF_NLP_FROM_MITOTIC_CENTROSOMES |
| 59 | 35 | 0.000479995 | 0.112378 | ST_FAS_SIGNALING_PATHWAY |
| 39 | 27 | 0.000859991 | 0.189162 | BIOCARTA_P38MAPK_PATHWAY |
| 50 | 30 | 0.000879991 | 0.190962 | REACTOME_TRAF6_MEDIATED_INDUCTION_OF_THE_ANTIVIRAL_CYTOKINE_IFN_ALPHA_BETA_CASCADE |
| 41 | 26 | 0.00114999 | 0.227355 | REACTOME_MAP_KINASES_ACTIVATION_IN_TLR_CASCADE |
| 126 | 67 | 0.00155998 | 0.293141 | KEGG_NEUROTROPHIN_SIGNALING_PATHWAY |
| 58 | 31 | 0.00195998 | 0.35073 | REACTOME_SIGNALING_BY_WNT |
| 56 | 31 | 0.00245998 | 0.418916 | REACTOME_TOLL_LIKE_RECEPTOR_3_CASCADE |
| 108 | 51 | 0.00395996 | 0.568286 | REACTOME_CELL_CYCLE_CHECKPOINTS |
| 54 | 30 | 0.00454995 | 0.615677 | BIOCARTA_PPARA_PATHWAY |
| 103 | 49 | 0.00460995 | 0.619476 | REACTOME_INNATE_IMMUNITY_SIGNALING |
| 27 | 19 | 0.00515995 | 0.65127 | BIOCARTA_PYK2_PATHWAY |
| 44 | 26 | 0.00527995 | 0.655469 | KEGG_AMINO_SUGAR_AND_NUCLEOTIDE_SUGAR_METABOLISM |
| 75 | 35 | 0.00554994 | 0.678664 | REACTOME_DNA_REPLICATION_PRE_INITIATION |
| 30 | 19 | 0.00713993 | 0.770846 | REACTOME_MAPK_TARGETS_NUCLEAR_EVENTS_MEDIATED_BY_MAP_KINASES |
| 90 | 47 | 0.00793992 | 0.80184 | REACTOME_MITOTIC_PROMETAPHASE |
| 120 | 57 | 0.00899991 | 0.830834 | REACTOME_HOST_INTERACTIONS_OF_HIV_FACTORS |
| 46 | 23 | 0.00912991 | 0.833633 | REACTOME_STABILIZATION_OF_P53 |
| 43 | 21 | 0.0098399 | 0.853829 | REACTOME_P53_INDEPENDENT_DNA_DAMAGE_RESPONSE |
| 58 | 28 | 0.0100299 | 0.858828 | REACTOME_CYCLIN_E_ASSOCIATED_EVENTS_DURING_G1_S_TRANSITION_ |
| 24 | 15 | 0.0101799 | 0.863627 | REACTOME_NUCLEAR_EVENTS_KINASE_AND_TRANSCRIPTION_FACTOR_ACTIVATION |
| 38 | 25 | 0.0126199 | 0.915217 | ST_JNK_MAPK_PATHWAY |
| 53 | 33 | 0.0131599 | 0.920616 | KEGG_NON_SMALL_CELL_LUNG_CANCER |
| 21 | 13 | 0.0149099 | 0.939212 | REACTOME_ERK_MAPK_TARGETS |
| 100 | 41 | 0.0150198 | 0.940012 | KEGG_TOLL_LIKE_RECEPTOR_SIGNALING_PATHWAY |
| 44 | 22 | 0.0153198 | 0.943411 | KEGG_PROTEASOME |
| 70 | 39 | 0.0154798 | 0.944611 | KEGG_PANCREATIC_CANCER |
| 47 | 22 | 0.0158698 | 0.946811 | REACTOME_REGULATION_OF_ORNITHINE_DECARBOXYLASE |
| 51 | 32 | 0.0163398 | 0.95121 | KEGG_INOSITOL_PHOSPHATE_METABOLISM |
| 61 | 28 | 0.0174098 | 0.957608 | REACTOME_M_G1_TRANSITION |
| 35 | 20 | 0.0190798 | 0.967606 | REACTOME_GENERIC_TRANSCRIPTION_PATHWAY |
| 74 | 35 | 0.0197098 | 0.970406 | KEGG_VEGF_SIGNALING_PATHWAY |
| 83 | 39 | 0.0202098 | 0.973005 | REACTOME_TOLL_RECEPTOR_CASCADES |
| 72 | 45 | 0.0203098 | 0.973005 | KEGG_PHOSPHATIDYLINOSITOL_SIGNALING_SYSTEM |
| 31 | 19 | 0.0206998 | 0.975405 | REACTOME_REV_MEDIATED_NUCLEAR_EXPORT_OF_HIV1_RNA |
| 52 | 24 | 0.0218598 | 0.979804 | REACTOME_CDT1_ASSOCIATION_WITH_THE_CDC6_ORC_ORIGIN_COMPLEX |
| 29 | 18 | 0.0223398 | 0.980604 | REACTOME_REGULATION_OF_GLUCOKINASE_BY_GLUCOKINASE_REGULATORY_PROTEIN |
| 100 | 44 | 0.0228398 | 0.982603 | REACTOME_G1_S_TRANSITION |
| 31 | 20 | 0.0230698 | 0.982603 | BIOCARTA_AT1R_PATHWAY |
| 29 | 18 | 0.0238598 | 0.984203 | REACTOME_NEP_NS2_INTERACTS_WITH_THE_CELLULAR_EXPORT_MACHINERY |
| 134 | 57 | 0.0245798 | 0.986403 | REACTOME_PROCESSING_OF_CAPPED_INTRON_CONTAINING_PRE_MRNA |
| 99 | 53 | 0.0249898 | 0.987602 | REACTOME_TRKA_SIGNALLING_FROM_THE_PLASMA_MEMBRANE |
| 108 | 52 | 0.0273197 | 0.991602 | KEGG_T_CELL_RECEPTOR_SIGNALING_PATHWAY |
| 52 | 24 | 0.0275897 | 0.992002 | REACTOME_SCF_SKP2_MEDIATED_DEGRADATION_OF_P27_P21 |
| 63 | 28 | 0.0281897 | 0.992801 | REACTOME_ORC1_REMOVAL_FROM_CHROMATIN |
| 35 | 19 | 0.0289197 | 0.994001 | REACTOME_DOWN_STREAM_SIGNAL_TRANSDUCTION |
| 183 | 77 | 0.0290397 | 0.994201 | REACTOME_HIV_INFECTION |
| 74 | 35 | 0.0305197 | 0.995401 | REACTOME_IRS_RELATED_EVENTS |
| 32 | 14 | 0.0310197 | 0.995401 | REACTOME_GENERATION_OF_SECOND_MESSENGER_MOLECULES |
| 77 | 39 | 0.0312397 | 0.995401 | KEGG_FC_EPSILON_RI_SIGNALING_PATHWAY |
| 90 | 42 | 0.0312497 | 0.995401 | REACTOME_LATE_PHASE_OF_HIV_LIFE_CYCLE |
| 99 | 52 | 0.0314497 | 0.995401 | KEGG_GNRH_SIGNALING_PATHWAY |
| 33 | 18 | 0.0320097 | 0.995601 | BIOCARTA_MPR_PATHWAY |
| 30 | 18 | 0.0329797 | 0.996201 | REACTOME_NUCLEAR_IMPORT_OF_REV_PROTEIN |
| 26 | 16 | 0.0329897 | 0.996201 | KEGG_GALACTOSE_METABOLISM |
| 88 | 45 | 0.0331997 | 0.996201 | KEGG_PROSTATE_CANCER |
| 120 | 54 | 0.0337697 | 0.996401 | KEGG_LYSOSOME |
| 58 | 27 | 0.0340197 | 0.996401 | REACTOME_TCR_SIGNALING |
| 37 | 19 | 0.0349397 | 0.996601 | KEGG_SNARE_INTERACTIONS_IN_VESICULAR_TRANSPORT |
| 63 | 34 | 0.0354196 | 0.996601 | SIG_PIP3_SIGNALING_IN_CARDIAC_MYOCTES |
| 84 | 40 | 0.0354296 | 0.996601 | KEGG_PROGESTERONE_MEDIATED_OOCYTE_MATURATION |
| 48 | 22 | 0.0358096 | 0.996801 | REACTOME_SCF_BETA_TRCP_MEDIATED_DEGRADATION_OF_EMI1 |
| 47 | 21 | 0.0359096 | 0.996801 | REACTOME_VIF_MEDIATED_DEGRADATION_OF_APOBEC3G |
| 36 | 19 | 0.0362196 | 0.997001 | REACTOME_ACTIVATION_OF_ATR_IN_RESPONSE_TO_REPLICATION_STRESS |
| 35 | 17 | 0.0370296 | 0.997201 | SIG_REGULATION_OF_THE_ACTIN_CYTOSKELETON_BY_RHO_GTPASES |
| 78 | 39 | 0.0373196 | 0.997201 | REACTOME_MEMBRANE_TRAFFICKING |
| 58 | 26 | 0.0376096 | 0.997201 | KEGG_NOD_LIKE_RECEPTOR_SIGNALING_PATHWAY |
| 70 | 31 | 0.0384496 | 0.998 | REACTOME_REGULATION_OF_APC_ACTIVATORS_BETWEEN_G1_S_AND_EARLY_ANAPHASE |
| 44 | 24 | 0.0386096 | 0.998 | ST_T_CELL_SIGNAL_TRANSDUCTION |
| 154 | 72 | 0.0412596 | 0.9982 | KEGG_ALZHEIMERS_DISEASE |
| 23 | 13 | 0.0413196 | 0.9984 | BIOCARTA_RAS_PATHWAY |
| 148 | 58 | 0.0422196 | 0.9988 | REACTOME_FORMATION_AND_MATURATION_OF_MRNA_TRANSCRIPT |
| 32 | 18 | 0.0433296 | 0.999 | REACTOME_TRANSPORT_OF_THE_SLBP_INDEPENDENT_MATURE_MRNA |
| 41 | 21 | 0.0444596 | 0.9992 | REACTOME_G2_M_CHECKPOINTS |
| 42 | 23 | 0.0454095 | 0.9994 | BIOCARTA_CHREBP2_PATHWAY |
| 66 | 35 | 0.0455995 | 0.9994 | KEGG_EPITHELIAL_CELL_SIGNALING_IN_HELICOBACTER_PYLORI_INFECTION |
| 40 | 19 | 0.0474895 | 0.9994 | REACTOME_MRNA_SPLICING_MINOR_PATHWAY |
| 62 | 27 | 0.0481795 | 0.9996 | REACTOME_CDC20_PHOSPHO_APC_MEDIATED_DEGRADATION_OF_CYCLIN_A |
| 39 | 23 | 0.0482495 | 0.9996 | ST_B_CELL_ANTIGEN_RECEPTOR |
| 26 | 14 | 0.0489495 | 0.9996 | BIOCARTA_RACCYCD_PATHWAY |
| 22 | 14 | 0.0490495 | 0.9996 | BIOCARTA_EIF4_PATHWAY |
| 35 | 20 | 0.0513295 | 0.9996 | BIOCARTA_FMLP_PATHWAY |
| 157 | 85 | 0.0529695 | 0.9996 | REACTOME_AXON_GUIDANCE |
| 29 | 17 | 0.0532095 | 0.9996 | REACTOME_TRANSPORT_OF_RIBONUCLEOPROTEINS_INTO_THE_HOST_NUCLEUS |
| 22 | 15 | 0.0542095 | 0.9996 | BIOCARTA_CCR3_PATHWAY |
| 49 | 26 | 0.0544295 | 0.9996 | SIG_INSULIN_RECEPTOR_PATHWAY_IN_CARDIAC_MYOCYTES |
| 78 | 43 | 0.0545195 | 0.9996 | ST_INTEGRIN_SIGNALING_PATHWAY |
| 30 | 17 | 0.0549595 | 1 | BIOCARTA_FAS_PATHWAY |
| 35 | 19 | 0.0567594 | 1 | ST_P38_MAPK_PATHWAY |
| 21 | 14 | 0.0568594 | 1 | REACTOME_STEROID_HORMONES |
| 67 | 26 | 0.0577494 | 1 | REACTOME_PHASE_1_FUNCTIONALIZATION_OF_COMPOUNDS |
| 20 | 11 | 0.0580594 | 1 | BIOCARTA_NKCELLS_PATHWAY |
| 133 | 58 | 0.0584294 | 1 | KEGG_UBIQUITIN_MEDIATED_PROTEOLYSIS |
| 20 | 10 | 0.0594394 | 1 | REACTOME_COMPLEMENT_CASCADE |
| 35 | 19 | 0.0615694 | 1 | ST_GA13_PATHWAY |
| 52 | 29 | 0.0618994 | 1 | KEGG_ENDOMETRIAL_CANCER |
| 39 | 18 | 0.0627794 | 1 | REACTOME_HIV1_TRANSCRIPTION_INITIATION |
| 70 | 35 | 0.0633094 | 1 | KEGG_RENAL_CELL_CARCINOMA |
| 161 | 67 | 0.0634294 | 1 | REACTOME_METABOLISM_OF_AMINO_ACIDS |
| 57 | 24 | 0.0653793 | 1 | REACTOME_AUTODEGRADATION_OF_CDH1_BY_CDH1_APC |
| 85 | 45 | 0.0664593 | 1 | KEGG_ERBB_SIGNALING_PATHWAY |
| 20 | 11 | 0.0724593 | 1 | REACTOME_DUAL_INCISION_REACTION_IN_GG_NER |
| 42 | 22 | 0.0744493 | 1 | KEGG_BLADDER_CANCER |
| 22 | 12 | 0.0777592 | 1 | BIOCARTA_MTOR_PATHWAY |
| 103 | 46 | 0.0790592 | 1 | REACTOME_HIV_LIFE_CYCLE |
| 26 | 15 | 0.0797692 | 1 | BIOCARTA_BAD_PATHWAY |
| 100 | 48 | 0.0807392 | 1 | KEGG_MELANOGENESIS |
| 183 | 73 | 0.0809492 | 1 | KEGG_CHEMOKINE_SIGNALING_PATHWAY |
| 23 | 13 | 0.0819892 | 1 | BIOCARTA_GLEEVEC_PATHWAY |
| 23 | 15 | 0.0842092 | 1 | BIOCARTA_RAC1_PATHWAY |
| 25 | 14 | 0.0842092 | 1 | BIOCARTA_WNT_PATHWAY |
| 22 | 12 | 0.0859791 | 1 | BIOCARTA_AKT_PATHWAY |
| 22 | 14 | 0.0860391 | 1 | BIOCARTA_HER2_PATHWAY |
| 29 | 16 | 0.0872691 | 1 | ST_ERK1_ERK2_MAPK_PATHWAY |
| 83 | 44 | 0.0878591 | 1 | KEGG_SMALL_CELL_LUNG_CANCER |
| 38 | 20 | 0.0886691 | 1 | REACTOME_GLUCOSE_TRANSPORT |
| 45 | 27 | 0.0919091 | 1 | BIOCARTA_KERATINOCYTE_PATHWAY |
| 33 | 16 | 0.0944391 | 1 | SIG_CD40PATHWAYMAP |
| 23 | 15 | 0.095069 | 1 | BIOCARTA_CXCR4_PATHWAY |
| 63 | 32 | 0.095379 | 1 | KEGG_GLIOMA |
| 20 | 11 | 0.097379 | 1 | REACTOME_RNA_POLYMERASE_I_PROMOTER_ESCAPE |
| 95 | 42 | 0.097649 | 1 | REACTOME_METABOLISM_OF_RNA |
| 56 | 21 | 0.098009 | 1 | KEGG_ARACHIDONIC_ACID_METABOLISM |
| 113 | 46 | 0.098129 | 1 | KEGG_OXIDATIVE_PHOSPHORYLATION |
| 33 | 21 | 0.098829 | 1 | SIG_PIP3_SIGNALING_IN_B_LYMPHOCYTES |
| 61 | 30 | 0.099109 | 1 | REACTOME_CLATHRIN_DERIVED_VESICLE_BUDDING |
| 29 | 14 | 0.099359 | 1 | REACTOME_RNA_POLYMERASE_III_TRANSCRIPTION_INITIATION |
| 71 | 34 | 0.100079 | 1 | KEGG_MELANOMA |
| 30 | 16 | 0.102859 | 1 | BIOCARTA_EGF_PATHWAY |
| 82 | 45 | 0.103269 | 1 | REACTOME_OPIOID_SIGNALLING |
| 23 | 12 | 0.103419 | 1 | BIOCARTA_G2_PATHWAY |
| 41 | 20 | 0.103759 | 1 | REACTOME_AMINE_LIGAND_BINDING_RECEPTORS |
| 44 | 22 | 0.104669 | 1 | SIG_CHEMOTAXIS |
| 35 | 17 | 0.107369 | 1 | BIOCARTA_CARM_ER_PATHWAY |
| 73 | 43 | 0.107799 | 1 | KEGG_ADHERENS_JUNCTION |
| 49 | 23 | 0.110779 | 1 | REACTOME_SNRNP_ASSEMBLY |
| 128 | 71 | 0.111419 | 1 | KEGG_AXON_GUIDANCE |
| 128 | 54 | 0.113679 | 1 | REACTOME_APOPTOSIS |
| 31 | 17 | 0.114139 | 1 | REACTOME_VPR_MEDIATED_NUCLEAR_IMPORT_OF_PICS |
| 53 | 27 | 0.115619 | 1 | BIOCARTA_NFAT_PATHWAY |
| 53 | 28 | 0.115749 | 1 | KEGG_AMYOTROPHIC_LATERAL_SCLEROSIS_ALS |
| 130 | 48 | 0.118119 | 1 | REACTOME_ELONGATION_AND_PROCESSING_OF_CAPPED_TRANSCRIPTS |
| 24 | 14 | 0.119409 | 1 | BIOCARTA_NTHI_PATHWAY |
| 72 | 35 | 0.119719 | 1 | KEGG_CHRONIC_MYELOID_LEUKEMIA |
| 21 | 10 | 0.120929 | 1 | REACTOME_CHOLESTEROL_BIOSYNTHESIS |
| 134 | 56 | 0.121569 | 1 | KEGG_INSULIN_SIGNALING_PATHWAY |
| 67 | 37 | 0.121879 | 1 | REACTOME_NCAM_SIGNALING_FOR_NEURITE_OUT_GROWTH |
| 34 | 17 | 0.122049 | 1 | REACTOME_SIGNALLING_TO_ERKS |
| 31 | 20 | 0.122369 | 1 | REACTOME_SIGNALING_BY_ROBO_RECEPTOR |
| 54 | 27 | 0.122559 | 1 | REACTOME_GOLGI_ASSOCIATED_VESICLE_BIOGENESIS |
| 23 | 13 | 0.123819 | 1 | REACTOME_FURTHER_PLATELET_RELEASATE |
| 62 | 31 | 0.124229 | 1 | REACTOME_SIGNALING_BY_PDGF |
| 26 | 15 | 0.124509 | 1 | BIOCARTA_CREB_PATHWAY |
| 62 | 33 | 0.125209 | 1 | KEGG_COLORECTAL_CANCER |
| 37 | 24 | 0.126189 | 1 | REACTOME_PLC_BETA_MEDIATED_EVENTS |
| 47 | 23 | 0.128259 | 1 | REACTOME_APOPTOTIC_EXECUTION_PHASE |
| 35 | 16 | 0.129959 | 1 | BIOCARTA_TOLL_PATHWAY |
| 149 | 69 | 0.131879 | 1 | KEGG_WNT_SIGNALING_PATHWAY |
| 62 | 30 | 0.132589 | 1 | REACTOME_STEROID_METABOLISM |
| 195 | 96 | 0.132619 | 1 | KEGG_FOCAL_ADHESION |
| 26 | 15 | 0.133489 | 1 | BIOCARTA_VIP_PATHWAY |
| 34 | 16 | 0.137869 | 1 | REACTOME_RNA_POLYMERASE_III_TRANSCRIPTION |
| 66 | 31 | 0.140899 | 1 | KEGG_P53_SIGNALING_PATHWAY |
| 21 | 11 | 0.142269 | 1 | BIOCARTA_IGF1_PATHWAY |
| 76 | 35 | 0.142829 | 1 | KEGG_PEROXISOME |
| 112 | 50 | 0.143259 | 1 | KEGG_LEUKOCYTE_TRANSENDOTHELIAL_MIGRATION |
| 42 | 19 | 0.143819 | 1 | KEGG_STARCH_AND_SUCROSE_METABOLISM |
| 44 | 21 | 0.144859 | 1 | KEGG_VASOPRESSIN_REGULATED_WATER_REABSORPTION |
| 31 | 15 | 0.146299 | 1 | BIOCARTA_PDGF_PATHWAY |
| 119 | 41 | 0.146569 | 1 | REACTOME_BIOLOGICAL_OXIDATIONS |
| 36 | 19 | 0.147829 | 1 | REACTOME_GENES_INVOLVED_IN_APOPTOTIC_CLEAVAGE_OF_CELLULAR_PROTEINS |
| 55 | 25 | 0.148059 | 1 | KEGG_RNA_DEGRADATION |
| 21 | 11 | 0.152248 | 1 | BIOCARTA_CYTOKINE_PATHWAY |
| 66 | 31 | 0.152658 | 1 | KEGG_ADIPOCYTOKINE_SIGNALING_PATHWAY |
| 26 | 12 | 0.154328 | 1 | REACTOME_SYNTHESIS_OF_GPI_ANCHORED_PROTEINS |
| 28 | 17 | 0.156918 | 1 | REACTOME_MYOGENESSIS |
| 27 | 13 | 0.157368 | 1 | BIOCARTA_GSK3_PATHWAY |
| 122 | 57 | 0.157778 | 1 | REACTOME_G_ALPHA_S_SIGNALLING_EVENTS |
| 52 | 26 | 0.158268 | 1 | REACTOME_HORMONE_BIOSYNTHESIS |
| 35 | 15 | 0.160178 | 1 | KEGG_BASAL_TRANSCRIPTION_FACTORS |
| 102 | 40 | 0.161198 | 1 | REACTOME_S_PHASE |
| 51 | 23 | 0.163538 | 1 | REACTOME_TRANSPORT_OF_MATURE_MRNA_DERIVED_FROM_AN_INTRON_CONTAINING_TRANSCRIPT |
| 167 | 71 | 0.166618 | 1 | KEGG_ENDOCYTOSIS |
| 24 | 12 | 0.168068 | 1 | KEGG_MATURITY_ONSET_DIABETES_OF_THE_YOUNG |
| 68 | 38 | 0.169128 | 1 | KEGG_LONG_TERM_DEPRESSION |
| 32 | 17 | 0.169248 | 1 | BIOCARTA_IL1R_PATHWAY |
| 30 | 14 | 0.169888 | 1 | KEGG_CITRATE_CYCLE_TCA_CYCLE |
| 26 | 13 | 0.170488 | 1 | KEGG_SELENOAMINO_ACID_METABOLISM |
| 26 | 12 | 0.171898 | 1 | REACTOME_RNA_POL_II_CTD_PHOSPHORYLATION_AND_INTERACTION_WITH_CE |
| 32 | 15 | 0.172138 | 1 | REACTOME_MRNA_PROCESSING |
| 24 | 7 | 0.173088 | 1 | REACTOME_PD1_SIGNALING |
| 62 | 23 | 0.173098 | 1 | KEGG_GLYCOLYSIS_GLUCONEOGENESIS |
| 116 | 32 | 0.173838 | 1 | REACTOME_RNA_POLYMERASE_I_III_AND_MITOCHONDRIAL_TRANSCRIPTION |
| 53 | 20 | 0.173928 | 1 | KEGG_ARGININE_AND_PROLINE_METABOLISM |
| 103 | 39 | 0.174098 | 1 | REACTOME_MRNA_SPLICING |
| 33 | 16 | 0.178208 | 1 | ST_PHOSPHOINOSITIDE_3_KINASE_PATHWAY |
| 38 | 20 | 0.178768 | 1 | BIOCARTA_INTEGRIN_PATHWAY |
| 88 | 34 | 0.178968 | 1 | REACTOME_SYNTHESIS_OF_DNA |
| 46 | 26 | 0.179328 | 1 | SIG_BCR_SIGNALING_PATHWAY |
| 35 | 15 | 0.184298 | 1 | REACTOME_PYRUVATE_METABOLISM_AND_TCA_CYCLE |
| 35 | 17 | 0.184608 | 1 | KEGG_PRION_DISEASES |
| 20 | 9 | 0.185328 | 1 | REACTOME_RNA_POLYMERASE_III_TRANSCRIPTION_INITIATION_FROM_TYPE_2_PROMOTER |
| 37 | 20 | 0.186938 | 1 | BIOCARTA_MET_PATHWAY |
| 56 | 25 | 0.189168 | 1 | KEGG_ACUTE_MYELOID_LEUKEMIA |
| 28 | 14 | 0.192538 | 1 | REACTOME_ASSOCIATION_OF_TRIC_CCT_WITH_TARGET_PROTEINS_DURING_BIOSYNTHESIS |
| 22 | 10 | 0.193208 | 1 | BIOCARTA_CELLCYCLE_PATHWAY |
| 34 | 16 | 0.193618 | 1 | REACTOME_PI3K_AKT_SIGNALLING |
| 40 | 15 | 0.195028 | 1 | REACTOME_POST_TRANSLATIONAL_PROTEIN_MODIFICATION |
| 24 | 12 | 0.195988 | 1 | REACTOME_RNA_POLYMERASE_I_TRANSCRIPTION_INITIATION |
| 23 | 12 | 0.196608 | 1 | BIOCARTA_IGF1R_PATHWAY |
| 91 | 43 | 0.197288 | 1 | KEGG_FC_GAMMA_R_MEDIATED_PHAGOCYTOSIS |
| 111 | 48 | 0.200808 | 1 | KEGG_OOCYTE_MEIOSIS |
| 59 | 28 | 0.203208 | 1 | REACTOME_PLATELET_ACTIVATION_TRIGGERS |
| 38 | 17 | 0.204568 | 1 | REACTOME_PI3K_CASCADE |
| 27 | 14 | 0.204648 | 1 | BIOCARTA_G1_PATHWAY |
| 49 | 18 | 0.204718 | 1 | REACTOME_CYTOCHROME_P450_ARRANGED_BY_SUBSTRATE_TYPE |
| 43 | 19 | 0.205338 | 1 | REACTOME_DOWNSTREAM_SIGNALING_OF_ACTIVATED_FGFR |
| 83 | 33 | 0.207788 | 1 | REACTOME_CLASS_B2_SECRETIN_FAMILY_RECEPTORS |
| 27 | 14 | 0.208138 | 1 | SIG_IL4RECEPTOR_IN_B_LYPHOCYTES |
| 22 | 10 | 0.209828 | 1 | REACTOME_RNA_POLYMERASE_III_TRANSCRIPTION_INITIATION_FROM_TYPE_3_PROMOTER |
| 27 | 14 | 0.212398 | 1 | ST_GAQ_PATHWAY |
| 22 | 11 | 0.212628 | 1 | BIOCARTA_CERAMIDE_PATHWAY |
| 48 | 24 | 0.213458 | 1 | REACTOME_SIGNALING_BY_EGFR |
| 27 | 12 | 0.214048 | 1 | REACTOME_FGFR_LIGAND_BINDING_AND_ACTIVATION |
| 117 | 43 | 0.214678 | 1 | REACTOME_METABOLISM_OF_CARBOHYDRATES |
| 173 | 64 | 0.215608 | 1 | REACTOME_G_ALPHA_I_SIGNALLING_EVENTS |
| 31 | 14 | 0.218018 | 1 | REACTOME_SIGNAL_AMPLIFICATION |
| 34 | 16 | 0.220888 | 1 | KEGG_BUTANOATE_METABOLISM |
| 29 | 18 | 0.223528 | 1 | KEGG_O_GLYCAN_BIOSYNTHESIS |
| 25 | 11 | 0.226938 | 1 | KEGG_GLYCOSYLPHOSPHATIDYLINOSITOL_GPI_ANCHOR_BIOSYNTHESIS |
| 56 | 26 | 0.231328 | 1 | BIOCARTA_HIVNEF_PATHWAY |
| 29 | 14 | 0.231568 | 1 | BIOCARTA_TNFR1_PATHWAY |
| 28 | 13 | 0.232058 | 1 | REACTOME_G_PROTEIN_ACTIVATION |
| 137 | 48 | 0.232098 | 1 | REACTOME_INFLUENZA_LIFE_CYCLE |
| 27 | 13 | 0.232998 | 1 | BIOCARTA_GH_PATHWAY |
| 23 | 14 | 0.233828 | 1 | ST_MYOCYTE_AD_PATHWAY |
| 32 | 14 | 0.234238 | 1 | BIOCARTA_RHO_PATHWAY |
| 22 | 10 | 0.236008 | 1 | BIOCARTA_P53HYPOXIA_PATHWAY |
| 68 | 22 | 0.239238 | 1 | KEGG_RIG_I_LIKE_RECEPTOR_SIGNALING_PATHWAY |
| 55 | 24 | 0.241898 | 1 | KEGG_BASAL_CELL_CARCINOMA |
| 24 | 13 | 0.242248 | 1 | SA_B_CELL_RECEPTOR_COMPLEXES |
| 22 | 12 | 0.242978 | 1 | REACTOME_PYRIMIDINE_METABOLISM |
| 43 | 20 | 0.245108 | 1 | BIOCARTA_TCR_PATHWAY |
| 29 | 12 | 0.247168 | 1 | REACTOME_ACTIVATION_OF_THE_PRE_REPLICATIVE_COMPLEX |
| 22 | 10 | 0.248738 | 1 | REACTOME_MTOR_SIGNALLING |
| 37 | 18 | 0.248868 | 1 | BIOCARTA_FCER1_PATHWAY |
| 22 | 11 | 0.249138 | 1 | ST_GA12_PATHWAY |
| 63 | 24 | 0.253527 | 1 | REACTOME_COSTIMULATION_BY_THE_CD28_FAMILY |
| 26 | 11 | 0.257597 | 1 | REACTOME_SIGNALLING_TO_RAS |
| 32 | 14 | 0.259077 | 1 | REACTOME_GLUCAGON_TYPE_LIGAND_RECEPTORS |
| 21 | 13 | 0.259577 | 1 | BIOCARTA_SPPA_PATHWAY |
| 23 | 12 | 0.260017 | 1 | KEGG_NICOTINATE_AND_NICOTINAMIDE_METABOLISM |
| 84 | 46 | 0.260337 | 1 | KEGG_ECM_RECEPTOR_INTERACTION |
| 28 | 9 | 0.260787 | 1 | KEGG_ASTHMA |
| 20 | 11 | 0.266637 | 1 | BIOCARTA_ATM_PATHWAY |
| 110 | 42 | 0.270187 | 1 | KEGG_PARKINSONS_DISEASE |
| 20 | 9 | 0.270287 | 1 | BIOCARTA_ACTINY_PATHWAY |
| 21 | 11 | 0.272467 | 1 | BIOCARTA_TFF_PATHWAY |
| 31 | 15 | 0.273977 | 1 | ST_WNT_BETA_CATENIN_PATHWAY |
| 41 | 15 | 0.275247 | 1 | KEGG_TYPE_I_DIABETES_MELLITUS |
| 198 | 79 | 0.276787 | 1 | REACTOME_REGULATION_OF_INSULIN_SECRETION |
| 34 | 18 | 0.277367 | 1 | ST_ADRENERGIC |
| 32 | 17 | 0.277947 | 1 | KEGG_PROPANOATE_METABOLISM |
| 81 | 18 | 0.278897 | 1 | KEGG_ANTIGEN_PROCESSING_AND_PRESENTATION |
| 184 | 81 | 0.280177 | 1 | REACTOME_FORMATION_OF_PLATELET_PLUG |
| 59 | 23 | 0.280697 | 1 | REACTOME_TRANSCRIPTION_OF_THE_HIV_GENOME |
| 23 | 11 | 0.282147 | 1 | BIOCARTA_TPO_PATHWAY |
| 46 | 23 | 0.284727 | 1 | KEGG_TYPE_II_DIABETES_MELLITUS |
| 74 | 34 | 0.287307 | 1 | KEGG_B_CELL_RECEPTOR_SIGNALING_PATHWAY |
| 33 | 12 | 0.289207 | 1 | REACTOME_RNA_POLYMERASE_I_CHAIN_ELONGATION |
| 41 | 16 | 0.292707 | 1 | KEGG_FATTY_ACID_METABOLISM |
| 61 | 32 | 0.293937 | 1 | REACTOME_REGULATION_OF_INSULIN_SECRETION_BY_GLUCAGON_LIKE_PEPTIDE_1 |
| 25 | 14 | 0.294127 | 1 | ST_GRANULE_CELL_SURVIVAL_PATHWAY |
| 49 | 25 | 0.294577 | 1 | REACTOME_NUCLEAR_RECEPTOR_TRANSCRIPTION_PATHWAY |
| 69 | 37 | 0.297517 | 1 | KEGG_LONG_TERM_POTENTIATION |
| 21 | 11 | 0.298967 | 1 | BIOCARTA_ATRBRCA_PATHWAY |
| 21 | 10 | 0.300917 | 1 | REACTOME_RNA_POLYMERASE_I_TRANSCRIPTION_TERMINATION |
| 42 | 19 | 0.301007 | 1 | ST_DIFFERENTIATION_PATHWAY_IN_PC12_CELLS |
| 41 | 19 | 0.303367 | 1 | BIOCARTA_BIOPEPTIDES_PATHWAY |
| 41 | 18 | 0.303887 | 1 | KEGG_ALDOSTERONE_REGULATED_SODIUM_REABSORPTION |
| 34 | 20 | 0.304667 | 1 | ST_G_ALPHA_I_PATHWAY |
| 32 | 15 | 0.305437 | 1 | KEGG_ALANINE_ASPARTATE_AND_GLUTAMATE_METABOLISM |
| 128 | 60 | 0.307587 | 1 | KEGG_TIGHT_JUNCTION |
| 122 | 48 | 0.310807 | 1 | KEGG_CELL_CYCLE |
| 54 | 17 | 0.311447 | 1 | KEGG_CYTOSOLIC_DNA_SENSING_PATHWAY |
| 38 | 17 | 0.316237 | 1 | KEGG_TRYPTOPHAN_METABOLISM |
| 28 | 13 | 0.316677 | 1 | BIOCARTA_ERK_PATHWAY |
| 46 | 20 | 0.317647 | 1 | REACTOME_METABOLISM_OF_MRNA |
| 23 | 9 | 0.322177 | 1 | KEGG_NITROGEN_METABOLISM |
| 27 | 11 | 0.325837 | 1 | REACTOME_FRS2MEDIATED_CASCADE |
| 33 | 14 | 0.329887 | 1 | BIOCARTA_DEATH_PATHWAY |
| 25 | 11 | 0.330447 | 1 | REACTOME_ADP_SIGNALLING_THROUGH_P2Y_PURINOCEPTOR_1 |
| 29 | 15 | 0.330907 | 1 | KEGG_THYROID_CANCER |
| 89 | 36 | 0.331387 | 1 | WNT_SIGNALING |
| 165 | 71 | 0.331897 | 1 | REACTOME_PLATELET_ACTIVATION |
| 22 | 9 | 0.332897 | 1 | BIOCARTA_CSK_PATHWAY |
| 41 | 15 | 0.333867 | 1 | REACTOME_DOWNSTREAM_TCR_SIGNALING |
| 29 | 11 | 0.334647 | 1 | BIOCARTA_INFLAM_PATHWAY |
| 26 | 13 | 0.342827 | 1 | REACTOME_DARPP32_EVENTS |
| 32 | 12 | 0.344037 | 1 | REACTOME_FORMATION_OF_THE_EARLY_ELONGATION_COMPLEX |
| 23 | 12 | 0.344607 | 1 | REACTOME_INTEGRIN_ALPHAIIBBETA3_SIGNALING |
| 21 | 9 | 0.346267 | 1 | BIOCARTA_MITOCHONDRIA_PATHWAY |
| 22 | 9 | 0.349627 | 1 | BIOCARTA_INSULIN_PATHWAY |
| 78 | 16 | 0.351196 | 1 | REACTOME_RNA_POLYMERASE_I_PROMOTER_CLEARANCE |
| 28 | 11 | 0.351706 | 1 | REACTOME_DUAL_INCISION_REACTION_IN_TC_NER |
| 36 | 20 | 0.352886 | 1 | BIOCARTA_AGR_PATHWAY |
| 153 | 65 | 0.353236 | 1 | REACTOME_G_ALPHA_Q_SIGNALLING_EVENTS |
| 53 | 24 | 0.354176 | 1 | KEGG_VIBRIO_CHOLERAE_INFECTION |
| 25 | 14 | 0.358376 | 1 | REACTOME_CAM_PATHWAY |
| 34 | 10 | 0.361996 | 1 | KEGG_REGULATION_OF_AUTOPHAGY |
| 24 | 12 | 0.362656 | 1 | BIOCARTA_ECM_PATHWAY |
| 68 | 29 | 0.363666 | 1 | KEGG_PPAR_SIGNALING_PATHWAY |
| 124 | 24 | 0.364086 | 1 | KEGG_SYSTEMIC_LUPUS_ERYTHEMATOSUS |
| 25 | 11 | 0.366126 | 1 | BIOCARTA_STRESS_PATHWAY |
| 95 | 38 | 0.366436 | 1 | KEGG_PYRIMIDINE_METABOLISM |
| 32 | 16 | 0.369176 | 1 | REACTOME_ACTIVATION_OF_KAINATE_RECEPTORS_UPON_GLUTAMATE_BINDING |
| 43 | 17 | 0.371186 | 1 | KEGG_LYSINE_DEGRADATION |
| 27 | 11 | 0.371656 | 1 | REACTOME_THROMBIN_SIGNALLING_THROUGH_PROTEINASE_ACTIVATED_RECEPTORS |
| 23 | 9 | 0.372036 | 1 | REACTOME_THROMBOXANE_SIGNALLING_THROUGH_TP_RECEPTOR |
| 33 | 16 | 0.372276 | 1 | BIOCARTA_BCR_PATHWAY |
| 20 | 10 | 0.374456 | 1 | REACTOME_PEROXISOMAL_LIPID_METABOLISM |
| 49 | 18 | 0.375466 | 1 | REACTOME_CHAPERONIN_MEDIATED_PROTEIN_FOLDING |
| 83 | 42 | 0.377866 | 1 | KEGG_HYPERTROPHIC_CARDIOMYOPATHY_HCM |
| 118 | 42 | 0.379286 | 1 | KEGG_SPLICEOSOME |
| 26 | 11 | 0.379566 | 1 | ST_INTERLEUKIN_4_PATHWAY |
| 65 | 32 | 0.381856 | 1 | REACTOME_SEMAPHORIN_INTERACTIONS |
| 36 | 16 | 0.384466 | 1 | KEGG_SPHINGOLIPID_METABOLISM |
| 28 | 8 | 0.386526 | 1 | KEGG_LINOLEIC_ACID_METABOLISM |
| 34 | 15 | 0.389206 | 1 | KEGG_CYSTEINE_AND_METHIONINE_METABOLISM |
| 32 | 15 | 0.390486 | 1 | REACTOME_GLUCAGON_SIGNALING_IN_METABOLIC_REGULATION |
| 37 | 18 | 0.391756 | 1 | BIOCARTA_ALK_PATHWAY |
| 46 | 19 | 0.393336 | 1 | KEGG_NOTCH_SIGNALING_PATHWAY |
| 83 | 40 | 0.394506 | 1 | REACTOME_CELL_JUNCTION_ORGANIZATION |
| 28 | 14 | 0.399346 | 1 | BIOCARTA_HDAC_PATHWAY |
| 31 | 13 | 0.400626 | 1 | REACTOME_E2F_MEDIATED_REGULATION_OF_DNA_REPLICATION |
| 131 | 41 | 0.404666 | 1 | KEGG_NATURAL_KILLER_CELL_MEDIATED_CYTOTOXICITY |
| 48 | 16 | 0.407626 | 1 | KEGG_TASTE_TRANSDUCTION |
| 42 | 22 | 0.408846 | 1 | REACTOME_NCAM1_INTERACTIONS |
| 21 | 6 | 0.413516 | 1 | REACTOME_PHOSPHORYLATION_OF_CD3_AND_TCR_ZETA_CHAINS |
| 192 | 56 | 0.413826 | 1 | REACTOME_TRANSCRIPTION |
| 40 | 17 | 0.415606 | 1 | KEGG_PYRUVATE_METABOLISM |
| 38 | 16 | 0.418196 | 1 | BIOCARTA_IL2RB_PATHWAY |
| 68 | 25 | 0.418566 | 1 | KEGG_VIRAL_MYOCARDITIS |
| 21 | 9 | 0.418626 | 1 | REACTOME_ADP_SIGNALLING_THROUGH_P2Y_PURINOCEPTOR_12 |
| 41 | 18 | 0.422116 | 1 | KEGG_AMINOACYL_TRNA_BIOSYNTHESIS |
| 27 | 11 | 0.422356 | 1 | REACTOME_LIPOPROTEIN_METABOLISM |
| 22 | 10 | 0.422846 | 1 | REACTOME_DEADENYLATION_OF_MRNA |
| 113 | 54 | 0.428576 | 1 | KEGG_VASCULAR_SMOOTH_MUSCLE_CONTRACTION |
| 86 | 33 | 0.430306 | 1 | KEGG_APOPTOSIS |
| 68 | 25 | 0.431086 | 1 | KEGG_COMPLEMENT_AND_COAGULATION_CASCADES |
| 175 | 83 | 0.432466 | 1 | KEGG_CALCIUM_SIGNALING_PATHWAY |
| 22 | 11 | 0.433976 | 1 | BIOCARTA_CTCF_PATHWAY |
| 34 | 20 | 0.438706 | 1 | REACTOME_PLC_GAMMA1_SIGNALLING |
| 90 | 45 | 0.439866 | 1 | KEGG_DILATED_CARDIOMYOPATHY |
| 66 | 21 | 0.440276 | 1 | KEGG_LEISHMANIA_INFECTION |
| 33 | 15 | 0.440966 | 1 | BIOCARTA_GPCR_PATHWAY |
| 33 | 13 | 0.443206 | 1 | REACTOME_GLOBAL_GENOMIC_NER |
| 20 | 11 | 0.443806 | 1 | REACTOME_REGULATION_OF_INSULIN_SECRETION_BY_FREE_FATTY_ACIDS |
| 20 | 8 | 0.444416 | 1 | REACTOME_E2F_TRANSCRIPTIONAL_TARGETS_AT_G1_S |
| 22 | 8 | 0.450045 | 1 | BIOCARTA_IL2_PATHWAY |
| 22 | 8 | 0.450255 | 1 | BIOCARTA_DC_PATHWAY |
| 48 | 18 | 0.458175 | 1 | KEGG_MTOR_SIGNALING_PATHWAY |
| 25 | 12 | 0.459295 | 1 | REACTOME_GS_ALPHA_MEDIATED_EVENTS_IN_GLUCAGON_SIGNALLING |
| 23 | 10 | 0.460565 | 1 | REACTOME_METAL_ION_SLC_TRANSPORTERS |
| 31 | 10 | 0.461465 | 1 | REACTOME_GLUCONEOGENESIS |
| 29 | 12 | 0.468115 | 1 | REACTOME_PURINE_METABOLISM |
| 44 | 16 | 0.470215 | 1 | KEGG_NUCLEOTIDE_EXCISION_REPAIR |
| 23 | 12 | 0.471715 | 1 | REACTOME_COLLAGEN_MEDIATED_ACTIVATION_CASCADE |
| 26 | 13 | 0.478715 | 1 | BIOCARTA_EDG1_PATHWAY |
| 21 | 8 | 0.481435 | 1 | KEGG_GLYCOSAMINOGLYCAN_DEGRADATION |
| 63 | 21 | 0.481995 | 1 | REACTOME_ELECTRON_TRANSPORT_CHAIN |
| 21 | 9 | 0.485085 | 1 | BIOCARTA_IL12_PATHWAY |
| 91 | 32 | 0.490305 | 1 | REACTOME_RNA_POLYMERASE_II_TRANSCRIPTION |
| 70 | 29 | 0.491015 | 1 | REACTOME_METABLISM_OF_NUCLEOTIDES |
| 26 | 12 | 0.494725 | 1 | REACTOME_PLATELET_AGGREGATION_PLUG_FORMATION |
| 21 | 9 | 0.496275 | 1 | REACTOME_NEF_MEDIATES_DOWN_MODULATION_OF_CELL_SURFACE_RECEPTORS_BY_RECRUITING_THEM_TO_CLATHRIN_ADAPTERS |
| 44 | 19 | 0.497985 | 1 | KEGG_ABC_TRANSPORTERS |
| 80 | 37 | 0.501325 | 1 | REACTOME_INTEGRIN_CELL_SURFACE_INTERACTIONS |
| 129 | 43 | 0.508165 | 1 | REACTOME_INSULIN_SYNTHESIS_AND_SECRETION |
| 147 | 52 | 0.512515 | 1 | REACTOME_GLUCOSE_REGULATION_OF_INSULIN_SECRETION |
| 83 | 41 | 0.514115 | 1 | REACTOME_NEURORANSMITTER_RECEPTOR_BINDING_AND_DOWNSTREAM_TRANSMISSION_IN_THE_POSTSYNAPTIC_CELL |
| 21 | 9 | 0.520405 | 1 | REACTOME_CYTOSOLIC_TRNA_AMINOACYLATION |
| 47 | 16 | 0.522235 | 1 | KEGG_STEROID_HORMONE_BIOSYNTHESIS |
| 27 | 13 | 0.522235 | 1 | REACTOME_METABOLISM_OF_BILE_ACIDS_AND_BILE_SALTS |
| 23 | 8 | 0.526775 | 1 | REACTOME_PHOSPHOLIPASE_CMEDIATED_CASCADE |
| 88 | 41 | 0.528145 | 1 | KEGG_GAP_JUNCTION |
| 169 | 61 | 0.530145 | 1 | KEGG_HUNTINGTONS_DISEASE |
| 82 | 38 | 0.532145 | 1 | REACTOME_P75_NTR_RECEPTOR_MEDIATED_SIGNALLING |
| 114 | 35 | 0.536035 | 1 | REACTOME_REGULATION_OF_BETA_CELL_DEVELOPMENT |
| 34 | 13 | 0.537575 | 1 | KEGG_FRUCTOSE_AND_MANNOSE_METABOLISM |
| 30 | 11 | 0.540075 | 1 | REACTOME_TIGHT_JUNCTION_INTERACTIONS |
| 85 | 29 | 0.541465 | 1 | KEGG_HEMATOPOIETIC_CELL_LINEAGE |
| 45 | 20 | 0.541655 | 1 | KEGG_N_GLYCAN_BIOSYNTHESIS |
| 38 | 16 | 0.544085 | 1 | REACTOME_TRNA_AMINOACYLATION |
| 47 | 25 | 0.546135 | 1 | REACTOME_NRAGE_SIGNALS_DEATH_THROUGH_JNK |
| 22 | 8 | 0.548955 | 1 | BIOCARTA_IL6_PATHWAY |
| 23 | 11 | 0.550134 | 1 | REACTOME_SMOOTH_MUSCLE_CONTRACTION |
| 155 | 66 | 0.550644 | 1 | KEGG_PURINE_METABOLISM |
| 28 | 9 | 0.551234 | 1 | REACTOME_GAP_JUNCTION_TRAFFICKING |
| 25 | 9 | 0.555414 | 1 | REACTOME_BASIGIN_INTERACTIONS |
| 56 | 20 | 0.563324 | 1 | KEGG_PATHOGENIC_ESCHERICHIA_COLI_INFECTION |
| 104 | 39 | 0.563384 | 1 | REACTOME_DNA_REPAIR |
| 29 | 14 | 0.565814 | 1 | REACTOME_AMINE_COMPOUND_SLC_TRANSPORTERS |
| 114 | 55 | 0.566664 | 1 | REACTOME_RHO_GTPASE_CYCLE |
| 52 | 16 | 0.569624 | 1 | REACTOME_PHASE_II_CONJUGATION |
| 128 | 63 | 0.571294 | 1 | REACTOME_TRANSMISSION_ACROSS_CHEMICAL_SYNAPSES |
| 35 | 10 | 0.575704 | 1 | KEGG_PRIMARY_IMMUNODEFICIENCY |
| 35 | 9 | 0.576314 | 1 | KEGG_ALLOGRAFT_REJECTION |
| 22 | 9 | 0.580484 | 1 | BIOCARTA_NFKB_PATHWAY |
| 21 | 10 | 0.590804 | 1 | REACTOME_DOUBLE_STRAND_BREAK_REPAIR |
| 70 | 27 | 0.600894 | 1 | KEGG_CARDIAC_MUSCLE_CONTRACTION |
| 54 | 27 | 0.603634 | 1 | REACTOME_G_ALPHA_12_13_SIGNALLING_EVENTS |
| 22 | 10 | 0.605404 | 1 | BIOCARTA_PGC1A_PATHWAY |
| 28 | 13 | 0.608394 | 1 | REACTOME_THE_ROLE_OF_NEF_IN_HIV1_REPLICATION_AND_DISEASE_PATHOGENESIS |
| 43 | 19 | 0.610584 | 1 | KEGG_VALINE_LEUCINE_AND_ISOLEUCINE_DEGRADATION |
| 59 | 27 | 0.611934 | 1 | REACTOME_CELL_CELL_ADHESION_SYSTEMS |
| 82 | 35 | 0.613154 | 1 | REACTOME_GLUCOSE_AND_OTHER_SUGAR_SLC_TRANSPORTERS |
| 24 | 10 | 0.615144 | 1 | REACTOME_LYSOSOME_VESICLE_BIOGENESIS |
| 28 | 9 | 0.618344 | 1 | BIOCARTA_NKT_PATHWAY |
| 23 | 8 | 0.618404 | 1 | BIOCARTA_CASPASE_PATHWAY |
| 74 | 39 | 0.628824 | 1 | KEGG_ARRHYTHMOGENIC_RIGHT_VENTRICULAR_CARDIOMYOPATHY_ARVC |
| 169 | 52 | 0.628904 | 1 | REACTOME_PEPTIDE_LIGAND_BINDING_RECEPTORS |
| 61 | 29 | 0.630204 | 1 | REACTOME_CELL_DEATH_SIGNALLING_VIA_NRAGE_NRIF_AND_NADE |
| 44 | 15 | 0.632414 | 1 | REACTOME_TRANSCRIPTION_COUPLED_NER |
| 22 | 6 | 0.633104 | 1 | REACTOME_GLYCOLYSIS |
| 20 | 10 | 0.633764 | 1 | BIOCARTA_NOS1_PATHWAY |
| 101 | 29 | 0.637034 | 1 | REACTOME_REGULATION_OF_GENE_EXPRESSION_IN_BETA_CELLS |
| 55 | 16 | 0.638794 | 1 | REACTOME_TRANSLATION_INITIATION_COMPLEX_FORMATION |
| 24 | 10 | 0.639304 | 1 | REACTOME_EGFR_DOWNREGULATION |
| 55 | 5 | 0.642004 | 1 | REACTOME_RNA_POLYMERASE_I_PROMOTER_OPENING |
| 22 | 12 | 0.642134 | 1 | REACTOME_REGULATION_OF_INSULIN_SECRETION_BY_ACETYLCHOLINE |
| 26 | 13 | 0.643094 | 1 | KEGG_GLYCOSAMINOGLYCAN_BIOSYNTHESIS_HEPARAN_SULFATE |
| 20 | 8 | 0.653343 | 1 | REACTOME_G_BETA_GAMMA_SIGNALLING_THROUGH_PLC_BETA |
| 56 | 21 | 0.656623 | 1 | KEGG_HEDGEHOG_SIGNALING_PATHWAY |
| 41 | 13 | 0.661673 | 1 | REACTOME_HIV1_TRANSCRIPTION_ELONGATION |
| 21 | 10 | 0.663043 | 1 | BIOCARTA_CHEMICAL_PATHWAY |
| 85 | 34 | 0.666163 | 1 | KEGG_TGF_BETA_SIGNALING_PATHWAY |
| 28 | 9 | 0.669843 | 1 | ST_TUMOR_NECROSIS_FACTOR_PATHWAY |
| 154 | 44 | 0.672803 | 1 | KEGG_JAK_STAT_SIGNALING_PATHWAY |
| 30 | 14 | 0.673473 | 1 | BIOCARTA_NO1_PATHWAY |
| 28 | 11 | 0.674783 | 1 | KEGG_HOMOLOGOUS_RECOMBINATION |
| 105 | 29 | 0.675813 | 1 | REACTOME_GTP_HYDROLYSIS_AND_JOINING_OF_THE_60S_RIBOSOMAL_SUBUNIT |
| 42 | 13 | 0.677703 | 1 | KEGG_TYROSINE_METABOLISM |
| 22 | 7 | 0.678003 | 1 | BIOCARTA_INTRINSIC_PATHWAY |
| 73 | 27 | 0.679013 | 1 | KEGG_GLYCEROPHOSPHOLIPID_METABOLISM |
| 29 | 9 | 0.680993 | 1 | KEGG_RNA_POLYMERASE |
| 23 | 9 | 0.686533 | 1 | BIOCARTA_PTDINS_PATHWAY |
| 23 | 7 | 0.690193 | 1 | REACTOME_TOLL_LIKE_RECEPTOR_9_CASCADE |
| 56 | 17 | 0.690423 | 1 | REACTOME_GLUCOSE_METABOLISM |
| 21 | 6 | 0.693803 | 1 | REACTOME_SHCMEDIATED_CASCADE |
| 22 | 6 | 0.694143 | 1 | REACTOME_FORMATION_OF_TUBULIN_FOLDING_INTERMEDIATES_BY_CCT_TRIC |
| 28 | 8 | 0.694693 | 1 | REACTOME_PREFOLDIN_MEDIATED_TRANSFER_OF_SUBSTRATE_TO_CCT_TRIC |
| 48 | 13 | 0.696773 | 1 | REACTOME_FORMATION_OF_THE_TERNARY_COMPLEX_AND_SUBSEQUENTLY_THE_43S_COMPLEX |
| 119 | 33 | 0.698143 | 1 | REACTOME_TRANSLATION |
| 21 | 9 | 0.698933 | 1 | KEGG_BIOSYNTHESIS_OF_UNSATURATED_FATTY_ACIDS |
| 23 | 9 | 0.702003 | 1 | KEGG_PROTEIN_EXPORT |
| 20 | 10 | 0.704483 | 1 | KEGG_GLYCOSAMINOGLYCAN_BIOSYNTHESIS_CHONDROITIN_SULFATE |
| 32 | 11 | 0.704653 | 1 | KEGG_ETHER_LIPID_METABOLISM |
| 46 | 19 | 0.706313 | 1 | KEGG_GLYCEROLIPID_METABOLISM |
| 33 | 10 | 0.706953 | 1 | KEGG_PORPHYRIN_AND_CHLOROPHYLL_METABOLISM |
| 94 | 35 | 0.707293 | 1 | REACTOME_CELL_SURFACE_INTERACTIONS_AT_THE_VASCULAR_WALL |
| 85 | 31 | 0.718573 | 1 | REACTOME_PLATELET_DEGRANULATION |
| 36 | 16 | 0.719943 | 1 | BIOCARTA_PAR1_PATHWAY |
| 50 | 19 | 0.723903 | 1 | REACTOME_MUSCLE_CONTRACTION |
| 29 | 16 | 0.729253 | 1 | REACTOME_ADHERENS_JUNCTIONS_INTERACTIONS |
| 27 | 11 | 0.730563 | 1 | REACTOME_CD28_CO_STIMULATION |
| 37 | 8 | 0.731103 | 1 | KEGG_GRAFT_VERSUS_HOST_DISEASE |
| 29 | 13 | 0.732443 | 1 | REACTOME_TRAFFICKING_OF_AMPA_RECEPTORS |
| 32 | 15 | 0.733723 | 1 | REACTOME_POST_NMDA_RECEPTOR_ACTIVATION_EVENTS |
| 25 | 10 | 0.734473 | 1 | BIOCARTA_MCALPAIN_PATHWAY |
| 169 | 69 | 0.734643 | 1 | REACTOME_SLC_MEDIATED_TRANSMEMBRANE_TRANSPORT |
| 28 | 10 | 0.736773 | 1 | REACTOME_SEMA4D_IN_SEMAPHORIN_SIGNALING |
| 29 | 9 | 0.737423 | 1 | KEGG_HISTIDINE_METABOLISM |
| 49 | 16 | 0.740323 | 1 | REACTOME_NUCLEOTIDE_EXCISION_REPAIR |
| 49 | 12 | 0.744293 | 1 | KEGG_GLUTATHIONE_METABOLISM |
| 33 | 10 | 0.746543 | 1 | KEGG_BASE_EXCISION_REPAIR |
| 23 | 7 | 0.746703 | 1 | REACTOME_ABORTIVE_ELONGATION_OF_HIV1_TRANSCRIPT_IN_THE_ABSENCE_OF_TAT |
| 26 | 11 | 0.755532 | 1 | REACTOME_CREB_PHOPHORYLATION_THROUGH_THE_ACTIVATION_OF_RAS |
| 86 | 22 | 0.760422 | 1 | KEGG_RIBOSOME |
| 31 | 10 | 0.761642 | 1 | REACTOME_STRIATED_MUSCLE_CONTRACTION |
| 36 | 17 | 0.773972 | 1 | REACTOME_ACTIVATION_OF_NMDA_RECEPTOR_UPON_GLUTAMATE_BINDING_AND_POSTSYNAPTIC_EVENTS |
| 23 | 8 | 0.778262 | 1 | REACTOME_SEMA4D_INDUCED_CELL_MIGRATION_AND_GROWTH_CONE_COLLAPSE |
| 100 | 26 | 0.778292 | 1 | REACTOME_INFLUENZA_VIRAL_RNA_TRANSCRIPTION_AND_REPLICATION |
| 52 | 10 | 0.779922 | 1 | REACTOME_CHEMOKINE_RECEPTORS_BIND_CHEMOKINES |
| 24 | 11 | 0.787682 | 1 | KEGG_DORSO_VENTRAL_AXIS_FORMATION |
| 55 | 12 | 0.790402 | 1 | KEGG_RETINOL_METABOLISM |
| 31 | 8 | 0.791232 | 1 | KEGG_GLYCINE_SERINE_AND_THREONINE_METABOLISM |
| 94 | 24 | 0.796812 | 1 | REACTOME_FORMATION_OF_A_POOL_OF_FREE_40S_SUBUNITS |
| 84 | 21 | 0.797812 | 1 | REACTOME_VIRAL_MRNA_TRANSLATION |
| 26 | 7 | 0.802352 | 1 | KEGG_PENTOSE_PHOSPHATE_PATHWAY |
| 30 | 12 | 0.815012 | 1 | BIOCARTA_MYOSIN_PATHWAY |
| 27 | 11 | 0.823402 | 1 | REACTOME_NEUROTRANSMITTER_RELEASE_CYCLE |
| 22 | 8 | 0.825872 | 1 | KEGG_BETA_ALANINE_METABOLISM |
| 20 | 8 | 0.828642 | 1 | REACTOME_MITOCHONDRIAL_TRNA_AMINOACYLATION |
| 28 | 9 | 0.834912 | 1 | BIOCARTA_VEGF_PATHWAY |
| 28 | 9 | 0.836962 | 1 | REACTOME_G_PROTEIN_BETA_GAMMA_SIGNALLING |
| 84 | 20 | 0.843502 | 1 | REACTOME_PEPTIDE_CHAIN_ELONGATION |
| 59 | 21 | 0.848132 | 1 | REACTOME_REGULATION_OF_LIPID_METABOLISM_BY_PEROXISOME_PROLIFERATOR_ACTIVATED_RECEPTOR_ALPHA |
| 25 | 7 | 0.852591 | 1 | REACTOME_G_BETA_GAMMA_SIGNALLING_THROUGH_PI3KGAMMA |
| 50 | 10 | 0.852781 | 1 | KEGG_AUTOIMMUNE_THYROID_DISEASE |
| 42 | 13 | 0.864741 | 1 | REACTOME_METABOLISM_OF_VITAMINS_AND_COFACTORS |
| 30 | 11 | 0.866761 | 1 | REACTOME_INHIBITION_OF_INSULIN_SECRETION_BY_ADRENALINE_NORADRENALINE |
| 32 | 9 | 0.869121 | 1 | REACTOME_FORMATION_OF_FIBRIN_CLOT_CLOTTING_CASCADE |
| 33 | 9 | 0.882081 | 1 | REACTOME_MRNA_3_END_PROCESSING |
| 48 | 16 | 0.884991 | 1 | REACTOME_AMINO_ACID_AND_OLIGOPEPTIDE_SLC_TRANSPORTERS |
| 46 | 12 | 0.885871 | 1 | KEGG_INTESTINAL_IMMUNE_NETWORK_FOR_IGA_PRODUCTION |
| 94 | 34 | 0.897261 | 1 | REACTOME_INORGANIC_CATION_ANION_SLC_TRANSPORTERS |
| 19 | 4 | 0.902471 | 1 | KEGG_PENTOSE_AND_GLUCURONATE_INTERCONVERSIONS |
| 29 | 12 | 0.902501 | 1 | REACTOME_SPHINGOLIPID_METABOLISM |
| 43 | 11 | 0.908651 | 1 | KEGG_DRUG_METABOLISM_OTHER_ENZYMES |
| 24 | 5 | 0.909451 | 1 | REACTOME_ACTIVATED_TLR4_SIGNALLING |
| 23 | 5 | 0.917051 | 1 | KEGG_PROXIMAL_TUBULE_BICARBONATE_RECLAMATION |
| 29 | 9 | 0.919011 | 1 | REACTOME_INTRINSIC_PATHWAY_FOR_APOPTOSIS |
| 46 | 4 | 0.919671 | 1 | REACTOME_PACKAGING_OF_TELOMERE_ENDS |
| 64 | 11 | 0.919891 | 1 | KEGG_DRUG_METABOLISM_CYTOCHROME_P450 |
| 26 | 7 | 0.921281 | 1 | KEGG_GLYCOSPHINGOLIPID_BIOSYNTHESIS_LACTO_AND_NEOLACTO_SERIES |
| 36 | 9 | 0.929451 | 1 | KEGG_DNA_REPLICATION |
| 21 | 6 | 0.934121 | 1 | REACTOME_CTLA4_INHIBITORY_SIGNALING |
| 17 | 3 | 0.938111 | 1 | KEGG_ASCORBATE_AND_ALDARATE_METABOLISM |
| 62 | 10 | 0.940171 | 1 | KEGG_METABOLISM_OF_XENOBIOTICS_BY_CYTOCHROME_P450 |
| 30 | 7 | 0.948251 | 1 | REACTOME_TAT_MEDIATED_HIV1_ELONGATION_ARREST_AND_RECOVERY |
| 28 | 6 | 0.95175 | 1 | REACTOME_TOLL_LIKE_RECEPTOR_4_CASCADE |
| 23 | 7 | 0.95369 | 1 | REACTOME_SIGNALING_BY_BMP |
| 131 | 45 | 0.95448 | 1 | KEGG_CELL_ADHESION_MOLECULES_CAMS |
| 68 | 11 | 0.96461 | 1 | REACTOME_IMMUNOREGULATORY_INTERACTIONS_BETWEEN_A_LYMPHOID_AND_A_NON_LYMPHOID_CELL |
| 30 | 7 | 0.96566 | 1 | REACTOME_DNA_STRAND_ELONGATION |
| 31 | 7 | 0.96994 | 1 | REACTOME_AMINO_ACID_TRANSPORT_ACROSS_THE_PLASMA_MEMBRANE |
| 20 | 4 | 0.97265 | 1 | REACTOME_LAGGING_STRAND_SYNTHESIS |
| 26 | 5 | 0.97321 | 1 | REACTOME_EXTENSION_OF_TELOMERES |
| 23 | 5 | 0.98076 | 1 | KEGG_MISMATCH_REPAIR |
| 72 | 9 | 0.98667 | 1 | REACTOME_TELOMERE_MAINTENANCE |
