# Supplementary material for: Whole-Genome Pathway Analysis on 132,497 Individuals Identifies Novel Gene-Sets Associated with Body Mass Index
Source: PLoS One. 2014 Jan 31;9(1):e78546. doi: 10.1371/journal.pone.0078546 (PMC3908858; doi:10.1371/journal.pone.0078546)
Supplement: Table S2 — INRICH Results for Discovery Set cutoff top 5%. (DOC) [file pone.0078546.s011.doc]

Table S2. Discovery INRICH results

INRICH Results for Discovery Set cutoff top 5%

| Target_Size | Int_No | Empirical_P | Corrected_P | Pathway |
| --- | --- | --- | --- | --- |
| 126 | 49 | 0.00140999 | 0.295141 | KEGG_NEUROTROPHIN_SIGNALING_PATHWAY |
| 72 | 30 | 0.00402996 | 0.587483 | KEGG_CHRONIC_MYELOID_LEUKEMIA |
| 86 | 33 | 0.00449996 | 0.623675 | BIOCARTA_MAPK_PATHWAY |
| 58 | 21 | 0.00637994 | 0.740252 | REACTOME_SIGNALING_BY_WNT |
| 55 | 22 | 0.00888991 | 0.845431 | KEGG_RNA_DEGRADATION |
| 50 | 20 | 0.00932991 | 0.857628 | REACTOME_TRAF6_MEDIATED_INDUCTION_OF_THE_ANTIVIRAL_CYTOKINE_IFN_ALPHA_BETA_CASCADE |
| 59 | 23 | 0.0095899 | 0.864227 | ST_FAS_SIGNALING_PATHWAY |
| 155 | 49 | 0.0106599 | 0.883823 | REACTOME_MITOTIC_M_M_G1_PHASES |
| 47 | 16 | 0.0129799 | 0.922815 | REACTOME_REGULATION_OF_ORNITHINE_DECARBOXYLASE |
| 22 | 11 | 0.0136999 | 0.933013 | ST_GA12_PATHWAY |
| 54 | 21 | 0.0141699 | 0.938212 | BIOCARTA_PPARA_PATHWAY |
| 79 | 28 | 0.0148399 | 0.943411 | REACTOME_G2_M_TRANSITION |
| 49 | 20 | 0.0153198 | 0.947211 | SIG_INSULIN_RECEPTOR_PATHWAY_IN_CARDIAC_MYOCYTES |
| 46 | 16 | 0.0158798 | 0.95041 | REACTOME_STABILIZATION_OF_P53 |
| 63 | 26 | 0.0159598 | 0.95081 | SIG_PIP3_SIGNALING_IN_CARDIAC_MYOCTES |
| 51 | 24 | 0.0159998 | 0.95161 | KEGG_INOSITOL_PHOSPHATE_METABOLISM |
| 41 | 17 | 0.0179098 | 0.965607 | REACTOME_MAP_KINASES_ACTIVATION_IN_TLR_CASCADE |
| 88 | 33 | 0.0191598 | 0.970806 | KEGG_PROSTATE_CANCER |
| 42 | 18 | 0.0203498 | 0.976405 | KEGG_BLADDER_CANCER |
| 35 | 15 | 0.0206198 | 0.977405 | REACTOME_GENERIC_TRANSCRIPTION_PATHWAY |
| 154 | 53 | 0.0213198 | 0.980004 | KEGG_ALZHEIMERS_DISEASE |
| 53 | 24 | 0.0215398 | 0.980204 | KEGG_NON_SMALL_CELL_LUNG_CANCER |
| 56 | 20 | 0.0224298 | 0.983003 | REACTOME_TOLL_LIKE_RECEPTOR_3_CASCADE |
| 38 | 17 | 0.0240998 | 0.985803 | BIOCARTA_INTEGRIN_PATHWAY |
| 58 | 19 | 0.0246498 | 0.987403 | REACTOME_CYCLIN_E_ASSOCIATED_EVENTS_DURING_G1_S_TRANSITION_ |
| 62 | 20 | 0.0254997 | 0.987802 | REACTOME_CDC20_PHOSPHO_APC_MEDIATED_DEGRADATION_OF_CYCLIN_A |
| 81 | 18 | 0.0257897 | 0.988402 | KEGG_ANTIGEN_PROCESSING_AND_PRESENTATION |
| 52 | 17 | 0.0276897 | 0.991002 | REACTOME_SCF_SKP2_MEDIATED_DEGRADATION_OF_P27_P21 |
| 57 | 18 | 0.0281697 | 0.991402 | REACTOME_AUTODEGRADATION_OF_CDH1_BY_CDH1_APC |
| 43 | 14 | 0.0290697 | 0.992202 | REACTOME_P53_INDEPENDENT_DNA_DAMAGE_RESPONSE |
| 48 | 20 | 0.0290797 | 0.992202 | REACTOME_SIGNALING_BY_EGFR |
| 120 | 38 | 0.0299897 | 0.994401 | KEGG_LYSOSOME |
| 30 | 13 | 0.0303897 | 0.994401 | REACTOME_MAPK_TARGETS_NUCLEAR_EVENTS_MEDIATED_BY_MAP_KINASES |
| 59 | 22 | 0.0304397 | 0.994401 | REACTOME_LOSS_OF_NLP_FROM_MITOTIC_CENTROSOMES |
| 44 | 15 | 0.0306497 | 0.994401 | KEGG_PROTEASOME |
| 22 | 12 | 0.0323397 | 0.995601 | BIOCARTA_CCR3_PATHWAY |
| 29 | 13 | 0.0331797 | 0.995601 | ST_ERK1_ERK2_MAPK_PATHWAY |
| 70 | 27 | 0.0359096 | 0.997401 | KEGG_PANCREATIC_CANCER |
| 61 | 19 | 0.0365896 | 0.9976 | REACTOME_M_G1_TRANSITION |
| 103 | 32 | 0.0376196 | 0.998 | REACTOME_INNATE_IMMUNITY_SIGNALING |
| 120 | 38 | 0.0379096 | 0.998 | REACTOME_HOST_INTERACTIONS_OF_HIV_FACTORS |
| 56 | 20 | 0.0405596 | 0.9986 | KEGG_ACUTE_MYELOID_LEUKEMIA |
| 21 | 9 | 0.0427796 | 0.999 | REACTOME_ERK_MAPK_TARGETS |
| 161 | 47 | 0.0428496 | 0.999 | REACTOME_METABOLISM_OF_AMINO_ACIDS |
| 67 | 23 | 0.0458095 | 0.9998 | REACTOME_CENTROSOME_MATURATION |
| 84 | 28 | 0.0464195 | 1 | KEGG_PROGESTERONE_MEDIATED_OOCYTE_MATURATION |
| 56 | 16 | 0.0465895 | 1 | KEGG_ARACHIDONIC_ACID_METABOLISM |
| 75 | 22 | 0.0469395 | 1 | REACTOME_DNA_REPLICATION_PRE_INITIATION |
| 155 | 54 | 0.0485095 | 1 | KEGG_PURINE_METABOLISM |
| 24 | 10 | 0.0490595 | 1 | REACTOME_NUCLEAR_EVENTS_KINASE_AND_TRANSCRIPTION_FACTOR_ACTIVATION |
| 108 | 32 | 0.0494195 | 1 | REACTOME_CELL_CYCLE_CHECKPOINTS |
| 48 | 15 | 0.0504995 | 1 | REACTOME_SCF_BETA_TRCP_MEDIATED_DEGRADATION_OF_EMI1 |
| 52 | 16 | 0.0515895 | 1 | REACTOME_CDT1_ASSOCIATION_WITH_THE_CDC6_ORC_ORIGIN_COMPLEX |
| 83 | 26 | 0.0543595 | 1 | REACTOME_TOLL_RECEPTOR_CASCADES |
| 133 | 41 | 0.0555394 | 1 | KEGG_UBIQUITIN_MEDIATED_PROTEOLYSIS |
| 70 | 21 | 0.0568794 | 1 | REACTOME_REGULATION_OF_APC_ACTIVATORS_BETWEEN_G1_S_AND_EARLY_ANAPHASE |
| 67 | 19 | 0.0573894 | 1 | REACTOME_PHASE_1_FUNCTIONALIZATION_OF_COMPOUNDS |
| 74 | 23 | 0.0617194 | 1 | KEGG_VEGF_SIGNALING_PATHWAY |
| 47 | 14 | 0.0630894 | 1 | REACTOME_VIF_MEDIATED_DEGRADATION_OF_APOBEC3G |
| 58 | 18 | 0.0635494 | 1 | KEGG_NOD_LIKE_RECEPTOR_SIGNALING_PATHWAY |
| 52 | 21 | 0.0636494 | 1 | KEGG_ENDOMETRIAL_CANCER |
| 62 | 18 | 0.0649194 | 1 | KEGG_GLYCOLYSIS_GLUCONEOGENESIS |
| 45 | 21 | 0.0659693 | 1 | BIOCARTA_KERATINOCYTE_PATHWAY |
| 68 | 24 | 0.0679993 | 1 | KEGG_PPAR_SIGNALING_PATHWAY |
| 22 | 9 | 0.0732793 | 1 | BIOCARTA_AKT_PATHWAY |
| 137 | 36 | 0.0743793 | 1 | REACTOME_INFLUENZA_LIFE_CYCLE |
| 29 | 13 | 0.0768092 | 1 | KEGG_THYROID_CANCER |
| 23 | 10 | 0.0797992 | 1 | REACTOME_FURTHER_PLATELET_RELEASATE |
| 157 | 60 | 0.0805192 | 1 | REACTOME_AXON_GUIDANCE |
| 24 | 11 | 0.0807692 | 1 | BIOCARTA_NTHI_PATHWAY |
| 128 | 52 | 0.0824692 | 1 | KEGG_AXON_GUIDANCE |
| 99 | 36 | 0.0827192 | 1 | REACTOME_TRKA_SIGNALLING_FROM_THE_PLASMA_MEMBRANE |
| 63 | 18 | 0.0840392 | 1 | REACTOME_ORC1_REMOVAL_FROM_CHROMATIN |
| 90 | 29 | 0.0870891 | 1 | REACTOME_MITOTIC_PROMETAPHASE |
| 22 | 9 | 0.0872691 | 1 | BIOCARTA_CERAMIDE_PATHWAY |
| 21 | 11 | 0.0892491 | 1 | BIOCARTA_SPPA_PATHWAY |
| 100 | 26 | 0.0908891 | 1 | KEGG_TOLL_LIKE_RECEPTOR_SIGNALING_PATHWAY |
| 27 | 11 | 0.0929691 | 1 | BIOCARTA_G1_PATHWAY |
| 23 | 11 | 0.096039 | 1 | BIOCARTA_CXCR4_PATHWAY |
| 46 | 16 | 0.096249 | 1 | REACTOME_METABOLISM_OF_MRNA |
| 25 | 9 | 0.098349 | 1 | KEGG_GLYCOSYLPHOSPHATIDYLINOSITOL_GPI_ANCHOR_BIOSYNTHESIS |
| 56 | 17 | 0.099239 | 1 | KEGG_PATHOGENIC_ESCHERICHIA_COLI_INFECTION |
| 54 | 13 | 0.100029 | 1 | KEGG_CYTOSOLIC_DNA_SENSING_PATHWAY |
| 100 | 28 | 0.100849 | 1 | REACTOME_G1_S_TRANSITION |
| 91 | 32 | 0.101379 | 1 | KEGG_FC_GAMMA_R_MEDIATED_PHAGOCYTOSIS |
| 83 | 31 | 0.101809 | 1 | KEGG_SMALL_CELL_LUNG_CANCER |
| 20 | 9 | 0.103059 | 1 | REACTOME_PEROXISOMAL_LIPID_METABOLISM |
| 31 | 13 | 0.103779 | 1 | BIOCARTA_AT1R_PATHWAY |
| 36 | 14 | 0.106679 | 1 | KEGG_SPHINGOLIPID_METABOLISM |
| 32 | 11 | 0.106999 | 1 | BIOCARTA_RHO_PATHWAY |
| 22 | 8 | 0.108359 | 1 | BIOCARTA_MTOR_PATHWAY |
| 55 | 18 | 0.108979 | 1 | KEGG_BASAL_CELL_CARCINOMA |
| 113 | 31 | 0.110589 | 1 | KEGG_OXIDATIVE_PHOSPHORYLATION |
| 26 | 9 | 0.110759 | 1 | BIOCARTA_RACCYCD_PATHWAY |
| 35 | 12 | 0.112859 | 1 | REACTOME_DOWN_STREAM_SIGNAL_TRANSDUCTION |
| 108 | 34 | 0.113699 | 1 | KEGG_T_CELL_RECEPTOR_SIGNALING_PATHWAY |
| 35 | 13 | 0.115559 | 1 | ST_GA13_PATHWAY |
| 33 | 12 | 0.115729 | 1 | ST_PHOSPHOINOSITIDE_3_KINASE_PATHWAY |
| 26 | 9 | 0.116329 | 1 | REACTOME_SYNTHESIS_OF_GPI_ANCHORED_PROTEINS |
| 74 | 22 | 0.121999 | 1 | REACTOME_IRS_RELATED_EVENTS |
| 27 | 11 | 0.122659 | 1 | BIOCARTA_PYK2_PATHWAY |
| 44 | 16 | 0.125689 | 1 | ST_T_CELL_SIGNAL_TRANSDUCTION |
| 21 | 6 | 0.126439 | 1 | REACTOME_PHOSPHORYLATION_OF_CD3_AND_TCR_ZETA_CHAINS |
| 63 | 22 | 0.127569 | 1 | KEGG_GLIOMA |
| 21 | 8 | 0.131989 | 1 | REACTOME_NEF_MEDIATES_DOWN_MODULATION_OF_CELL_SURFACE_RECEPTORS_BY_RECRUITING_THEM_TO_CLATHRIN_ADAPTERS |
| 99 | 35 | 0.132349 | 1 | KEGG_GNRH_SIGNALING_PATHWAY |
| 31 | 12 | 0.132469 | 1 | REACTOME_REV_MEDIATED_NUCLEAR_EXPORT_OF_HIV1_RNA |
| 100 | 33 | 0.133169 | 1 | KEGG_MELANOGENESIS |
| 22 | 9 | 0.133319 | 1 | BIOCARTA_EIF4_PATHWAY |
| 72 | 30 | 0.133509 | 1 | KEGG_PHOSPHATIDYLINOSITOL_SIGNALING_SYSTEM |
| 28 | 7 | 0.134429 | 1 | KEGG_LINOLEIC_ACID_METABOLISM |
| 33 | 15 | 0.134519 | 1 | SIG_PIP3_SIGNALING_IN_B_LYMPHOCYTES |
| 35 | 13 | 0.136349 | 1 | BIOCARTA_FMLP_PATHWAY |
| 40 | 11 | 0.138599 | 1 | REACTOME_POST_TRANSLATIONAL_PROTEIN_MODIFICATION |
| 34 | 12 | 0.142279 | 1 | REACTOME_SIGNALLING_TO_ERKS |
| 29 | 10 | 0.144399 | 1 | REACTOME_PURINE_METABOLISM |
| 22 | 8 | 0.146119 | 1 | BIOCARTA_P53HYPOXIA_PATHWAY |
| 62 | 23 | 0.147369 | 1 | KEGG_COLORECTAL_CANCER |
| 66 | 22 | 0.148999 | 1 | KEGG_ADIPOCYTOKINE_SIGNALING_PATHWAY |
| 35 | 13 | 0.149189 | 1 | KEGG_PRION_DISEASES |
| 62 | 21 | 0.150148 | 1 | REACTOME_STEROID_METABOLISM |
| 173 | 44 | 0.153278 | 1 | REACTOME_G_ALPHA_I_SIGNALLING_EVENTS |
| 49 | 14 | 0.154228 | 1 | REACTOME_CHAPERONIN_MEDIATED_PROTEIN_FOLDING |
| 66 | 17 | 0.154288 | 1 | KEGG_LEISHMANIA_INFECTION |
| 70 | 23 | 0.155248 | 1 | KEGG_RENAL_CELL_CARCINOMA |
| 67 | 26 | 0.157248 | 1 | REACTOME_NCAM_SIGNALING_FOR_NEURITE_OUT_GROWTH |
| 32 | 9 | 0.157968 | 1 | REACTOME_GENERATION_OF_SECOND_MESSENGER_MOLECULES |
| 38 | 15 | 0.158868 | 1 | ST_JNK_MAPK_PATHWAY |
| 95 | 28 | 0.159268 | 1 | REACTOME_METABOLISM_OF_RNA |
| 34 | 16 | 0.161108 | 1 | ST_G_ALPHA_I_PATHWAY |
| 102 | 27 | 0.163578 | 1 | REACTOME_S_PHASE |
| 122 | 34 | 0.165758 | 1 | KEGG_CELL_CYCLE |
| 53 | 20 | 0.169098 | 1 | KEGG_AMYOTROPHIC_LATERAL_SCLEROSIS_ALS |
| 88 | 23 | 0.172698 | 1 | REACTOME_SYNTHESIS_OF_DNA |
| 37 | 14 | 0.174128 | 1 | BIOCARTA_ALK_PATHWAY |
| 61 | 20 | 0.175448 | 1 | REACTOME_CLATHRIN_DERIVED_VESICLE_BUDDING |
| 27 | 9 | 0.177228 | 1 | BIOCARTA_GSK3_PATHWAY |
| 20 | 7 | 0.177628 | 1 | BIOCARTA_ACTINY_PATHWAY |
| 39 | 15 | 0.177848 | 1 | ST_B_CELL_ANTIGEN_RECEPTOR |
| 23 | 8 | 0.178868 | 1 | BIOCARTA_RAS_PATHWAY |
| 29 | 11 | 0.179078 | 1 | REACTOME_TRANSPORT_OF_RIBONUCLEOPROTEINS_INTO_THE_HOST_NUCLEUS |
| 101 | 23 | 0.180048 | 1 | REACTOME_REGULATION_OF_GENE_EXPRESSION_IN_BETA_CELLS |
| 134 | 37 | 0.181678 | 1 | KEGG_INSULIN_SIGNALING_PATHWAY |
| 48 | 14 | 0.181838 | 1 | KEGG_MTOR_SIGNALING_PATHWAY |
| 29 | 11 | 0.183108 | 1 | REACTOME_NEP_NS2_INTERACTS_WITH_THE_CELLULAR_EXPORT_MACHINERY |
| 23 | 7 | 0.186138 | 1 | KEGG_NITROGEN_METABOLISM |
| 25 | 9 | 0.189238 | 1 | BIOCARTA_WNT_PATHWAY |
| 128 | 44 | 0.190448 | 1 | KEGG_TIGHT_JUNCTION |
| 28 | 10 | 0.191848 | 1 | REACTOME_ASSOCIATION_OF_TRIC_CCT_WITH_TARGET_PROTEINS_DURING_BIOSYNTHESIS |
| 27 | 9 | 0.191938 | 1 | REACTOME_LIPOPROTEIN_METABOLISM |
| 183 | 49 | 0.192778 | 1 | REACTOME_HIV_INFECTION |
| 49 | 13 | 0.193488 | 1 | REACTOME_CYTOCHROME_P450_ARRANGED_BY_SUBSTRATE_TYPE |
| 27 | 10 | 0.196718 | 1 | SIG_IL4RECEPTOR_IN_B_LYPHOCYTES |
| 22 | 6 | 0.198868 | 1 | REACTOME_GLYCOLYSIS |
| 44 | 14 | 0.199798 | 1 | KEGG_VASOPRESSIN_REGULATED_WATER_REABSORPTION |
| 22 | 7 | 0.200128 | 1 | BIOCARTA_CELLCYCLE_PATHWAY |
| 42 | 14 | 0.200388 | 1 | ST_DIFFERENTIATION_PATHWAY_IN_PC12_CELLS |
| 42 | 14 | 0.200568 | 1 | BIOCARTA_CHREBP2_PATHWAY |
| 26 | 9 | 0.202098 | 1 | KEGG_SELENOAMINO_ACID_METABOLISM |
| 37 | 14 | 0.203888 | 1 | BIOCARTA_MET_PATHWAY |
| 78 | 24 | 0.206188 | 1 | REACTOME_MEMBRANE_TRAFFICKING |
| 100 | 22 | 0.206538 | 1 | REACTOME_INFLUENZA_VIRAL_RNA_TRANSCRIPTION_AND_REPLICATION |
| 30 | 11 | 0.207278 | 1 | REACTOME_NUCLEAR_IMPORT_OF_REV_PROTEIN |
| 27 | 11 | 0.208088 | 1 | REACTOME_METABOLISM_OF_BILE_ACIDS_AND_BILE_SALTS |
| 39 | 14 | 0.209138 | 1 | BIOCARTA_P38MAPK_PATHWAY |
| 22 | 7 | 0.210228 | 1 | REACTOME_MTOR_SIGNALLING |
| 32 | 12 | 0.212158 | 1 | BIOCARTA_IL1R_PATHWAY |
| 114 | 26 | 0.212678 | 1 | REACTOME_REGULATION_OF_BETA_CELL_DEVELOPMENT |
| 26 | 8 | 0.215698 | 1 | REACTOME_SIGNALLING_TO_RAS |
| 105 | 23 | 0.216198 | 1 | REACTOME_GTP_HYDROLYSIS_AND_JOINING_OF_THE_60S_RIBOSOMAL_SUBUNIT |
| 95 | 27 | 0.220458 | 1 | KEGG_PYRIMIDINE_METABOLISM |
| 38 | 12 | 0.221708 | 1 | BIOCARTA_IL2RB_PATHWAY |
| 41 | 13 | 0.225848 | 1 | REACTOME_AMINE_LIGAND_BINDING_RECEPTORS |
| 31 | 8 | 0.226598 | 1 | REACTOME_GLUCONEOGENESIS |
| 32 | 11 | 0.231328 | 1 | REACTOME_TRANSPORT_OF_THE_SLBP_INDEPENDENT_MATURE_MRNA |
| 24 | 8 | 0.238128 | 1 | KEGG_MATURITY_ONSET_DIABETES_OF_THE_YOUNG |
| 68 | 15 | 0.238628 | 1 | KEGG_RIG_I_LIKE_RECEPTOR_SIGNALING_PATHWAY |
| 122 | 38 | 0.243078 | 1 | REACTOME_G_ALPHA_S_SIGNALLING_EVENTS |
| 28 | 12 | 0.244048 | 1 | REACTOME_MYOGENESSIS |
| 33 | 10 | 0.246828 | 1 | BIOCARTA_MPR_PATHWAY |
| 68 | 27 | 0.247338 | 1 | KEGG_LONG_TERM_DEPRESSION |
| 41 | 12 | 0.251297 | 1 | KEGG_TYPE_I_DIABETES_MELLITUS |
| 34 | 10 | 0.254287 | 1 | REACTOME_RNA_POLYMERASE_III_TRANSCRIPTION |
| 112 | 33 | 0.254677 | 1 | KEGG_LEUKOCYTE_TRANSENDOTHELIAL_MIGRATION |
| 27 | 9 | 0.255367 | 1 | BIOCARTA_GH_PATHWAY |
| 46 | 14 | 0.258837 | 1 | KEGG_NOTCH_SIGNALING_PATHWAY |
| 24 | 5 | 0.260067 | 1 | REACTOME_PD1_SIGNALING |
| 29 | 13 | 0.261717 | 1 | KEGG_O_GLYCAN_BIOSYNTHESIS |
| 111 | 32 | 0.262517 | 1 | KEGG_OOCYTE_MEIOSIS |
| 41 | 14 | 0.263057 | 1 | KEGG_AMINOACYL_TRNA_BIOSYNTHESIS |
| 73 | 29 | 0.264917 | 1 | KEGG_ADHERENS_JUNCTION |
| 51 | 15 | 0.268127 | 1 | REACTOME_TRANSPORT_OF_MATURE_MRNA_DERIVED_FROM_AN_INTRON_CONTAINING_TRANSCRIPT |
| 119 | 27 | 0.268527 | 1 | REACTOME_BIOLOGICAL_OXIDATIONS |
| 175 | 61 | 0.271327 | 1 | KEGG_CALCIUM_SIGNALING_PATHWAY |
| 27 | 10 | 0.271497 | 1 | ST_GAQ_PATHWAY |
| 89 | 25 | 0.273037 | 1 | WNT_SIGNALING |
| 38 | 13 | 0.275337 | 1 | REACTOME_TRNA_AMINOACYLATION |
| 21 | 7 | 0.275497 | 1 | BIOCARTA_IGF1_PATHWAY |
| 30 | 9 | 0.280717 | 1 | KEGG_CITRATE_CYCLE_TCA_CYCLE |
| 85 | 29 | 0.282507 | 1 | KEGG_ERBB_SIGNALING_PATHWAY |
| 29 | 10 | 0.283427 | 1 | REACTOME_REGULATION_OF_GLUCOKINASE_BY_GLUCOKINASE_REGULATORY_PROTEIN |
| 26 | 10 | 0.284667 | 1 | REACTOME_CREB_PHOPHORYLATION_THROUGH_THE_ACTIVATION_OF_RAS |
| 58 | 16 | 0.284687 | 1 | REACTOME_TCR_SIGNALING |
| 23 | 8 | 0.285197 | 1 | REACTOME_METAL_ION_SLC_TRANSPORTERS |
| 52 | 17 | 0.291347 | 1 | REACTOME_HORMONE_BIOSYNTHESIS |
| 76 | 22 | 0.292687 | 1 | KEGG_PEROXISOME |
| 41 | 13 | 0.292927 | 1 | BIOCARTA_BIOPEPTIDES_PATHWAY |
| 69 | 27 | 0.293627 | 1 | KEGG_LONG_TERM_POTENTIATION |
| 21 | 6 | 0.294747 | 1 | REACTOME_CHOLESTEROL_BIOSYNTHESIS |
| 134 | 34 | 0.295227 | 1 | REACTOME_PROCESSING_OF_CAPPED_INTRON_CONTAINING_PRE_MRNA |
| 35 | 10 | 0.296547 | 1 | BIOCARTA_TOLL_PATHWAY |
| 31 | 10 | 0.297537 | 1 | ST_WNT_BETA_CATENIN_PATHWAY |
| 23 | 8 | 0.299607 | 1 | KEGG_NICOTINATE_AND_NICOTINAMIDE_METABOLISM |
| 54 | 17 | 0.302157 | 1 | REACTOME_GOLGI_ASSOCIATED_VESICLE_BIOGENESIS |
| 29 | 8 | 0.302787 | 1 | REACTOME_RNA_POLYMERASE_III_TRANSCRIPTION_INITIATION |
| 83 | 29 | 0.304187 | 1 | REACTOME_CELL_JUNCTION_ORGANIZATION |
| 65 | 23 | 0.306307 | 1 | REACTOME_SEMAPHORIN_INTERACTIONS |
| 22 | 6 | 0.307637 | 1 | BIOCARTA_IL2_PATHWAY |
| 33 | 9 | 0.310407 | 1 | KEGG_BASE_EXCISION_REPAIR |
| 119 | 24 | 0.312427 | 1 | REACTOME_TRANSLATION |
| 20 | 6 | 0.312867 | 1 | REACTOME_COMPLEMENT_CASCADE |
| 24 | 8 | 0.315087 | 1 | REACTOME_EGFR_DOWNREGULATION |
| 43 | 13 | 0.320027 | 1 | BIOCARTA_TCR_PATHWAY |
| 49 | 18 | 0.321017 | 1 | REACTOME_NUCLEAR_RECEPTOR_TRANSCRIPTION_PATHWAY |
| 59 | 21 | 0.321177 | 1 | REACTOME_CELL_CELL_ADHESION_SYSTEMS |
| 53 | 16 | 0.324627 | 1 | BIOCARTA_NFAT_PATHWAY |
| 20 | 6 | 0.324867 | 1 | BIOCARTA_NKCELLS_PATHWAY |
| 84 | 17 | 0.325187 | 1 | REACTOME_VIRAL_MRNA_TRANSLATION |
| 46 | 17 | 0.338197 | 1 | SIG_BCR_SIGNALING_PATHWAY |
| 53 | 12 | 0.338547 | 1 | KEGG_ARGININE_AND_PROLINE_METABOLISM |
| 26 | 10 | 0.339947 | 1 | BIOCARTA_EDG1_PATHWAY |
| 40 | 12 | 0.342637 | 1 | KEGG_PYRUVATE_METABOLISM |
| 28 | 9 | 0.342697 | 1 | KEGG_HOMOLOGOUS_RECOMBINATION |
| 85 | 24 | 0.347167 | 1 | REACTOME_PLATELET_DEGRANULATION |
| 56 | 17 | 0.347577 | 1 | BIOCARTA_HIVNEF_PATHWAY |
| 22 | 7 | 0.349157 | 1 | REACTOME_DEADENYLATION_OF_MRNA |
| 20 | 7 | 0.350396 | 1 | BIOCARTA_ATM_PATHWAY |
| 83 | 30 | 0.350486 | 1 | REACTOME_NEURORANSMITTER_RECEPTOR_BINDING_AND_DOWNSTREAM_TRANSMISSION_IN_THE_POSTSYNAPTIC_CELL |
| 169 | 43 | 0.352296 | 1 | KEGG_HUNTINGTONS_DISEASE |
| 183 | 45 | 0.353616 | 1 | KEGG_CHEMOKINE_SIGNALING_PATHWAY |
| 39 | 10 | 0.356126 | 1 | REACTOME_HIV1_TRANSCRIPTION_INITIATION |
| 84 | 32 | 0.361706 | 1 | KEGG_ECM_RECEPTOR_INTERACTION |
| 35 | 9 | 0.361896 | 1 | SIG_REGULATION_OF_THE_ACTIN_CYTOSKELETON_BY_RHO_GTPASES |
| 34 | 10 | 0.362766 | 1 | REACTOME_PI3K_AKT_SIGNALLING |
| 77 | 23 | 0.364636 | 1 | KEGG_FC_EPSILON_RI_SIGNALING_PATHWAY |
| 110 | 27 | 0.366786 | 1 | KEGG_PARKINSONS_DISEASE |
| 78 | 26 | 0.370866 | 1 | ST_INTEGRIN_SIGNALING_PATHWAY |
| 86 | 17 | 0.371256 | 1 | KEGG_RIBOSOME |
| 22 | 6 | 0.371846 | 1 | BIOCARTA_INTRINSIC_PATHWAY |
| 74 | 22 | 0.376396 | 1 | KEGG_B_CELL_RECEPTOR_SIGNALING_PATHWAY |
| 66 | 20 | 0.377006 | 1 | KEGG_EPITHELIAL_CELL_SIGNALING_IN_HELICOBACTER_PYLORI_INFECTION |
| 83 | 20 | 0.378356 | 1 | REACTOME_CLASS_B2_SECRETIN_FAMILY_RECEPTORS |
| 62 | 19 | 0.378806 | 1 | REACTOME_SIGNALING_BY_PDGF |
| 21 | 8 | 0.379326 | 1 | REACTOME_STEROID_HORMONES |
| 32 | 11 | 0.389726 | 1 | REACTOME_ACTIVATION_OF_KAINATE_RECEPTORS_UPON_GLUTAMATE_BINDING |
| 22 | 7 | 0.393846 | 1 | BIOCARTA_NFKB_PATHWAY |
| 56 | 13 | 0.394406 | 1 | REACTOME_GLUCOSE_METABOLISM |
| 71 | 21 | 0.399876 | 1 | KEGG_MELANOMA |
| 28 | 10 | 0.400796 | 1 | REACTOME_THE_ROLE_OF_NEF_IN_HIV1_REPLICATION_AND_DISEASE_PATHOGENESIS |
| 85 | 25 | 0.402686 | 1 | KEGG_TGF_BETA_SIGNALING_PATHWAY |
| 90 | 24 | 0.404366 | 1 | REACTOME_LATE_PHASE_OF_HIV_LIFE_CYCLE |
| 117 | 27 | 0.404406 | 1 | REACTOME_METABOLISM_OF_CARBOHYDRATES |
| 20 | 5 | 0.404496 | 1 | REACTOME_RNA_POLYMERASE_III_TRANSCRIPTION_INITIATION_FROM_TYPE_2_PROMOTER |
| 29 | 7 | 0.409216 | 1 | KEGG_RNA_POLYMERASE |
| 55 | 12 | 0.409996 | 1 | REACTOME_TRANSLATION_INITIATION_COMPLEX_FORMATION |
| 84 | 16 | 0.410836 | 1 | REACTOME_PEPTIDE_CHAIN_ELONGATION |
| 31 | 10 | 0.416846 | 1 | REACTOME_VPR_MEDIATED_NUCLEAR_IMPORT_OF_PICS |
| 131 | 40 | 0.418196 | 1 | KEGG_CELL_ADHESION_MOLECULES_CAMS |
| 33 | 9 | 0.420086 | 1 | SIG_CD40PATHWAYMAP |
| 29 | 13 | 0.420816 | 1 | REACTOME_ADHERENS_JUNCTIONS_INTERACTIONS |
| 21 | 7 | 0.421056 | 1 | BIOCARTA_TFF_PATHWAY |
| 23 | 7 | 0.422166 | 1 | BIOCARTA_GLEEVEC_PATHWAY |
| 30 | 8 | 0.422836 | 1 | REACTOME_TIGHT_JUNCTION_INTERACTIONS |
| 24 | 8 | 0.428896 | 1 | BIOCARTA_ECM_PATHWAY |
| 23 | 7 | 0.429336 | 1 | BIOCARTA_TPO_PATHWAY |
| 35 | 8 | 0.430416 | 1 | KEGG_ALLOGRAFT_REJECTION |
| 129 | 29 | 0.431086 | 1 | REACTOME_INSULIN_SYNTHESIS_AND_SECRETION |
| 23 | 8 | 0.431366 | 1 | REACTOME_INTEGRIN_ALPHAIIBBETA3_SIGNALING |
| 36 | 14 | 0.434076 | 1 | REACTOME_ACTIVATION_OF_NMDA_RECEPTOR_UPON_GLUTAMATE_BINDING_AND_POSTSYNAPTIC_EVENTS |
| 29 | 11 | 0.445466 | 1 | REACTOME_SPHINGOLIPID_METABOLISM |
| 195 | 62 | 0.447806 | 1 | KEGG_FOCAL_ADHESION |
| 49 | 13 | 0.449446 | 1 | REACTOME_SNRNP_ASSEMBLY |
| 40 | 10 | 0.451815 | 1 | REACTOME_MRNA_SPLICING_MINOR_PATHWAY |
| 30 | 9 | 0.454925 | 1 | BIOCARTA_EGF_PATHWAY |
| 23 | 9 | 0.457825 | 1 | ST_MYOCYTE_AD_PATHWAY |
| 32 | 12 | 0.457955 | 1 | REACTOME_POST_NMDA_RECEPTOR_ACTIVATION_EVENTS |
| 128 | 32 | 0.459715 | 1 | REACTOME_APOPTOSIS |
| 20 | 7 | 0.460855 | 1 | REACTOME_MITOCHONDRIAL_TRNA_AMINOACYLATION |
| 22 | 8 | 0.466585 | 1 | BIOCARTA_HER2_PATHWAY |
| 24 | 8 | 0.467355 | 1 | SA_B_CELL_RECEPTOR_COMPLEXES |
| 23 | 6 | 0.468175 | 1 | BIOCARTA_G2_PATHWAY |
| 167 | 45 | 0.469815 | 1 | KEGG_ENDOCYTOSIS |
| 68 | 18 | 0.470585 | 1 | KEGG_VIRAL_MYOCARDITIS |
| 44 | 13 | 0.472645 | 1 | KEGG_ABC_TRANSPORTERS |
| 94 | 18 | 0.472985 | 1 | REACTOME_FORMATION_OF_A_POOL_OF_FREE_40S_SUBUNITS |
| 26 | 8 | 0.475655 | 1 | BIOCARTA_BAD_PATHWAY |
| 25 | 9 | 0.475975 | 1 | ST_GRANULE_CELL_SURVIVAL_PATHWAY |
| 28 | 6 | 0.479375 | 1 | REACTOME_PREFOLDIN_MEDIATED_TRANSFER_OF_SUBSTRATE_TO_CCT_TRIC |
| 50 | 10 | 0.481475 | 1 | KEGG_AUTOIMMUNE_THYROID_DISEASE |
| 35 | 10 | 0.481825 | 1 | ST_P38_MAPK_PATHWAY |
| 28 | 8 | 0.481915 | 1 | BIOCARTA_ERK_PATHWAY |
| 35 | 7 | 0.483105 | 1 | KEGG_PRIMARY_IMMUNODEFICIENCY |
| 83 | 29 | 0.485815 | 1 | KEGG_HYPERTROPHIC_CARDIOMYOPATHY_HCM |
| 38 | 11 | 0.486795 | 1 | REACTOME_GLUCOSE_TRANSPORT |
| 42 | 15 | 0.488245 | 1 | REACTOME_NCAM1_INTERACTIONS |
| 56 | 15 | 0.493025 | 1 | KEGG_HEDGEHOG_SIGNALING_PATHWAY |
| 34 | 14 | 0.495135 | 1 | REACTOME_PLC_GAMMA1_SIGNALLING |
| 37 | 15 | 0.496275 | 1 | REACTOME_PLC_BETA_MEDIATED_EVENTS |
| 25 | 6 | 0.499045 | 1 | REACTOME_BASIGIN_INTERACTIONS |
| 33 | 10 | 0.501125 | 1 | BIOCARTA_BCR_PATHWAY |
| 35 | 8 | 0.504045 | 1 | KEGG_BASAL_TRANSCRIPTION_FACTORS |
| 25 | 7 | 0.507665 | 1 | BIOCARTA_STRESS_PATHWAY |
| 29 | 7 | 0.510725 | 1 | BIOCARTA_INFLAM_PATHWAY |
| 36 | 9 | 0.511045 | 1 | REACTOME_ACTIVATION_OF_ATR_IN_RESPONSE_TO_REPLICATION_STRESS |
| 25 | 9 | 0.514075 | 1 | REACTOME_CAM_PATHWAY |
| 31 | 12 | 0.515155 | 1 | REACTOME_SIGNALING_BY_ROBO_RECEPTOR |
| 149 | 42 | 0.519885 | 1 | KEGG_WNT_SIGNALING_PATHWAY |
| 47 | 11 | 0.521855 | 1 | KEGG_STEROID_HORMONE_BIOSYNTHESIS |
| 59 | 16 | 0.523295 | 1 | REACTOME_REGULATION_OF_LIPID_METABOLISM_BY_PEROXISOME_PROLIFERATOR_ACTIVATED_RECEPTOR_ALPHA |
| 34 | 6 | 0.527125 | 1 | KEGG_REGULATION_OF_AUTOPHAGY |
| 82 | 27 | 0.527265 | 1 | REACTOME_OPIOID_SIGNALLING |
| 26 | 8 | 0.529775 | 1 | REACTOME_PLATELET_AGGREGATION_PLUG_FORMATION |
| 113 | 37 | 0.530845 | 1 | KEGG_VASCULAR_SMOOTH_MUSCLE_CONTRACTION |
| 28 | 7 | 0.531395 | 1 | REACTOME_G_PROTEIN_ACTIVATION |
| 21 | 5 | 0.534355 | 1 | REACTOME_ADP_SIGNALLING_THROUGH_P2Y_PURINOCEPTOR_12 |
| 21 | 7 | 0.538945 | 1 | REACTOME_DOUBLE_STRAND_BREAK_REPAIR |
| 44 | 12 | 0.541605 | 1 | KEGG_AMINO_SUGAR_AND_NUCLEOTIDE_SUGAR_METABOLISM |
| 32 | 8 | 0.544705 | 1 | REACTOME_MRNA_PROCESSING |
| 22 | 5 | 0.545325 | 1 | BIOCARTA_DC_PATHWAY |
| 63 | 13 | 0.546705 | 1 | REACTOME_ELECTRON_TRANSPORT_CHAIN |
| 27 | 6 | 0.549385 | 1 | REACTOME_THROMBIN_SIGNALLING_THROUGH_PROTEINASE_ACTIVATED_RECEPTORS |
| 49 | 9 | 0.554574 | 1 | KEGG_GLUTATHIONE_METABOLISM |
| 21 | 6 | 0.556814 | 1 | REACTOME_CYTOSOLIC_TRNA_AMINOACYLATION |
| 104 | 26 | 0.558064 | 1 | REACTOME_DNA_REPAIR |
| 20 | 5 | 0.558264 | 1 | REACTOME_E2F_TRANSCRIPTIONAL_TARGETS_AT_G1_S |
| 41 | 10 | 0.559164 | 1 | REACTOME_G2_M_CHECKPOINTS |
| 31 | 8 | 0.559684 | 1 | BIOCARTA_PDGF_PATHWAY |
| 22 | 5 | 0.562504 | 1 | REACTOME_RNA_POLYMERASE_III_TRANSCRIPTION_INITIATION_FROM_TYPE_3_PROMOTER |
| 31 | 8 | 0.563114 | 1 | REACTOME_E2F_MEDIATED_REGULATION_OF_DNA_REPLICATION |
| 66 | 17 | 0.568244 | 1 | KEGG_P53_SIGNALING_PATHWAY |
| 22 | 4 | 0.569644 | 1 | REACTOME_FORMATION_OF_TUBULIN_FOLDING_INTERMEDIATES_BY_CCT_TRIC |
| 37 | 9 | 0.569884 | 1 | KEGG_SNARE_INTERACTIONS_IN_VESICULAR_TRANSPORT |
| 23 | 5 | 0.571744 | 1 | REACTOME_TOLL_LIKE_RECEPTOR_9_CASCADE |
| 22 | 5 | 0.573444 | 1 | BIOCARTA_INSULIN_PATHWAY |
| 22 | 5 | 0.576444 | 1 | BIOCARTA_IL6_PATHWAY |
| 55 | 9 | 0.576554 | 1 | KEGG_RETINOL_METABOLISM |
| 20 | 7 | 0.576584 | 1 | KEGG_GLYCOSAMINOGLYCAN_BIOSYNTHESIS_CHONDROITIN_SULFATE |
| 103 | 26 | 0.579134 | 1 | REACTOME_HIV_LIFE_CYCLE |
| 21 | 5 | 0.582054 | 1 | KEGG_GLYCOSAMINOGLYCAN_DEGRADATION |
| 24 | 7 | 0.583484 | 1 | REACTOME_LYSOSOME_VESICLE_BIOGENESIS |
| 26 | 6 | 0.584194 | 1 | REACTOME_RNA_POL_II_CTD_PHOSPHORYLATION_AND_INTERACTION_WITH_CE |
| 21 | 6 | 0.585444 | 1 | BIOCARTA_ATRBRCA_PATHWAY |
| 23 | 7 | 0.586034 | 1 | REACTOME_SMOOTH_MUSCLE_CONTRACTION |
| 169 | 34 | 0.587454 | 1 | REACTOME_PEPTIDE_LIGAND_BINDING_RECEPTORS |
| 41 | 9 | 0.589874 | 1 | KEGG_FATTY_ACID_METABOLISM |
| 50 | 13 | 0.589984 | 1 | REACTOME_MUSCLE_CONTRACTION |
| 37 | 7 | 0.596594 | 1 | KEGG_GRAFT_VERSUS_HOST_DISEASE |
| 68 | 16 | 0.597524 | 1 | KEGG_COMPLEMENT_AND_COAGULATION_CASCADES |
| 41 | 9 | 0.600634 | 1 | REACTOME_DOWNSTREAM_TCR_SIGNALING |
| 33 | 9 | 0.601354 | 1 | BIOCARTA_GPCR_PATHWAY |
| 85 | 19 | 0.602034 | 1 | KEGG_HEMATOPOIETIC_CELL_LINEAGE |
| 116 | 18 | 0.606114 | 1 | REACTOME_RNA_POLYMERASE_I_III_AND_MITOCHONDRIAL_TRANSCRIPTION |
| 32 | 10 | 0.606554 | 1 | KEGG_PROPANOATE_METABOLISM |
| 26 | 6 | 0.617284 | 1 | ST_INTERLEUKIN_4_PATHWAY |
| 31 | 7 | 0.617744 | 1 | REACTOME_STRIATED_MUSCLE_CONTRACTION |
| 48 | 9 | 0.623304 | 1 | REACTOME_FORMATION_OF_THE_TERNARY_COMPLEX_AND_SUBSEQUENTLY_THE_43S_COMPLEX |
| 46 | 14 | 0.623644 | 1 | KEGG_TYPE_II_DIABETES_MELLITUS |
| 22 | 5 | 0.630074 | 1 | BIOCARTA_CSK_PATHWAY |
| 36 | 10 | 0.631114 | 1 | REACTOME_GENES_INVOLVED_IN_APOPTOTIC_CLEAVAGE_OF_CELLULAR_PROTEINS |
| 128 | 43 | 0.631674 | 1 | REACTOME_TRANSMISSION_ACROSS_CHEMICAL_SYNAPSES |
| 90 | 30 | 0.637604 | 1 | KEGG_DILATED_CARDIOMYOPATHY |
| 23 | 6 | 0.641554 | 1 | BIOCARTA_IGF1R_PATHWAY |
| 26 | 7 | 0.642044 | 1 | KEGG_GALACTOSE_METABOLISM |
| 82 | 25 | 0.644444 | 1 | REACTOME_P75_NTR_RECEPTOR_MEDIATED_SIGNALLING |
| 20 | 5 | 0.646304 | 1 | REACTOME_G_BETA_GAMMA_SIGNALLING_THROUGH_PLC_BETA |
| 103 | 22 | 0.654403 | 1 | REACTOME_MRNA_SPLICING |
| 73 | 18 | 0.654513 | 1 | KEGG_GLYCEROPHOSPHOLIPID_METABOLISM |
| 53 | 14 | 0.656423 | 1 | KEGG_VIBRIO_CHOLERAE_INFECTION |
| 45 | 12 | 0.657333 | 1 | KEGG_N_GLYCAN_BIOSYNTHESIS |
| 21 | 5 | 0.661163 | 1 | BIOCARTA_IL12_PATHWAY |
| 148 | 31 | 0.662173 | 1 | REACTOME_FORMATION_AND_MATURATION_OF_MRNA_TRANSCRIPT |
| 124 | 15 | 0.664393 | 1 | KEGG_SYSTEMIC_LUPUS_ERYTHEMATOSUS |
| 23 | 5 | 0.668193 | 1 | REACTOME_ABORTIVE_ELONGATION_OF_HIV1_TRANSCRIPT_IN_THE_ABSENCE_OF_TAT |
| 22 | 6 | 0.669343 | 1 | REACTOME_PYRIMIDINE_METABOLISM |
| 154 | 28 | 0.675233 | 1 | KEGG_JAK_STAT_SIGNALING_PATHWAY |
| 26 | 7 | 0.676753 | 1 | BIOCARTA_VIP_PATHWAY |
| 29 | 9 | 0.682083 | 1 | REACTOME_TRAFFICKING_OF_AMPA_RECEPTORS |
| 24 | 8 | 0.683043 | 1 | KEGG_DORSO_VENTRAL_AXIS_FORMATION |
| 29 | 6 | 0.686823 | 1 | KEGG_HISTIDINE_METABOLISM |
| 26 | 7 | 0.688533 | 1 | REACTOME_DARPP32_EVENTS |
| 23 | 6 | 0.688563 | 1 | BIOCARTA_PTDINS_PATHWAY |
| 82 | 23 | 0.692583 | 1 | REACTOME_GLUCOSE_AND_OTHER_SUGAR_SLC_TRANSPORTERS |
| 48 | 9 | 0.696473 | 1 | KEGG_TASTE_TRANSDUCTION |
| 192 | 34 | 0.703943 | 1 | REACTOME_TRANSCRIPTION |
| 28 | 6 | 0.708303 | 1 | REACTOME_G_PROTEIN_BETA_GAMMA_SIGNALLING |
| 33 | 7 | 0.708713 | 1 | BIOCARTA_DEATH_PATHWAY |
| 22 | 6 | 0.710383 | 1 | BIOCARTA_CTCF_PATHWAY |
| 37 | 9 | 0.711923 | 1 | BIOCARTA_FCER1_PATHWAY |
| 70 | 17 | 0.713333 | 1 | REACTOME_METABLISM_OF_NUCLEOTIDES |
| 131 | 25 | 0.718193 | 1 | KEGG_NATURAL_KILLER_CELL_MEDIATED_CYTOTOXICITY |
| 22 | 6 | 0.719003 | 1 | BIOCARTA_PGC1A_PATHWAY |
| 28 | 5 | 0.719193 | 1 | REACTOME_GAP_JUNCTION_TRAFFICKING |
| 74 | 27 | 0.720273 | 1 | KEGG_ARRHYTHMOGENIC_RIGHT_VENTRICULAR_CARDIOMYOPATHY_ARVC |
| 35 | 7 | 0.721413 | 1 | REACTOME_PYRUVATE_METABOLISM_AND_TCA_CYCLE |
| 55 | 3 | 0.724653 | 1 | REACTOME_RNA_POLYMERASE_I_PROMOTER_OPENING |
| 23 | 4 | 0.726123 | 1 | REACTOME_THROMBOXANE_SIGNALLING_THROUGH_TP_RECEPTOR |
| 44 | 11 | 0.728253 | 1 | SIG_CHEMOTAXIS |
| 34 | 8 | 0.730543 | 1 | KEGG_CYSTEINE_AND_METHIONINE_METABOLISM |
| 34 | 10 | 0.730773 | 1 | ST_ADRENERGIC |
| 32 | 7 | 0.736143 | 1 | KEGG_ETHER_LIPID_METABOLISM |
| 20 | 4 | 0.736373 | 1 | REACTOME_DUAL_INCISION_REACTION_IN_GG_NER |
| 21 | 5 | 0.736643 | 1 | BIOCARTA_CYTOKINE_PATHWAY |
| 165 | 43 | 0.738313 | 1 | REACTOME_PLATELET_ACTIVATION |
| 62 | 8 | 0.742663 | 1 | KEGG_METABOLISM_OF_XENOBIOTICS_BY_CYTOCHROME_P450 |
| 198 | 47 | 0.747633 | 1 | REACTOME_REGULATION_OF_INSULIN_SECRETION |
| 30 | 9 | 0.748083 | 1 | BIOCARTA_NO1_PATHWAY |
| 28 | 6 | 0.748383 | 1 | REACTOME_SEMA4D_IN_SEMAPHORIN_SIGNALING |
| 42 | 9 | 0.751482 | 1 | KEGG_STARCH_AND_SUCROSE_METABOLISM |
| 153 | 39 | 0.751482 | 1 | REACTOME_G_ALPHA_Q_SIGNALLING_EVENTS |
| 28 | 5 | 0.754982 | 1 | BIOCARTA_NKT_PATHWAY |
| 36 | 7 | 0.757022 | 1 | KEGG_DNA_REPLICATION |
| 47 | 11 | 0.759862 | 1 | REACTOME_APOPTOTIC_EXECUTION_PHASE |
| 43 | 9 | 0.760172 | 1 | REACTOME_DOWNSTREAM_SIGNALING_OF_ACTIVATED_FGFR |
| 91 | 19 | 0.760922 | 1 | REACTOME_RNA_POLYMERASE_II_TRANSCRIPTION |
| 20 | 4 | 0.764492 | 1 | REACTOME_RNA_POLYMERASE_I_PROMOTER_ESCAPE |
| 32 | 6 | 0.765822 | 1 | REACTOME_GLUCAGON_TYPE_LIGAND_RECEPTORS |
| 25 | 6 | 0.770752 | 1 | REACTOME_GS_ALPHA_MEDIATED_EVENTS_IN_GLUCAGON_SIGNALLING |
| 88 | 26 | 0.770922 | 1 | KEGG_GAP_JUNCTION |
| 25 | 4 | 0.771152 | 1 | REACTOME_G_BETA_GAMMA_SIGNALLING_THROUGH_PI3KGAMMA |
| 35 | 8 | 0.771492 | 1 | BIOCARTA_CARM_ER_PATHWAY |
| 30 | 7 | 0.771642 | 1 | BIOCARTA_FAS_PATHWAY |
| 147 | 31 | 0.772822 | 1 | REACTOME_GLUCOSE_REGULATION_OF_INSULIN_SECRETION |
| 70 | 17 | 0.774212 | 1 | KEGG_CARDIAC_MUSCLE_CONTRACTION |
| 31 | 6 | 0.774862 | 1 | REACTOME_SIGNAL_AMPLIFICATION |
| 184 | 48 | 0.783052 | 1 | REACTOME_FORMATION_OF_PLATELET_PLUG |
| 59 | 12 | 0.783292 | 1 | REACTOME_TRANSCRIPTION_OF_THE_HIV_GENOME |
| 130 | 25 | 0.786392 | 1 | REACTOME_ELONGATION_AND_PROCESSING_OF_CAPPED_TRANSCRIPTS |
| 52 | 9 | 0.788972 | 1 | REACTOME_PHASE_II_CONJUGATION |
| 28 | 7 | 0.789212 | 1 | BIOCARTA_HDAC_PATHWAY |
| 32 | 6 | 0.789892 | 1 | REACTOME_FORMATION_OF_THE_EARLY_ELONGATION_COMPLEX |
| 64 | 8 | 0.800212 | 1 | KEGG_DRUG_METABOLISM_CYTOCHROME_P450 |
| 25 | 6 | 0.801222 | 1 | BIOCARTA_MCALPAIN_PATHWAY |
| 41 | 9 | 0.803302 | 1 | KEGG_ALDOSTERONE_REGULATED_SODIUM_REABSORPTION |
| 20 | 6 | 0.806572 | 1 | BIOCARTA_NOS1_PATHWAY |
| 169 | 45 | 0.807292 | 1 | REACTOME_SLC_MEDIATED_TRANSMEMBRANE_TRANSPORT |
| 29 | 5 | 0.809302 | 1 | REACTOME_ACTIVATION_OF_THE_PRE_REPLICATIVE_COMPLEX |
| 28 | 5 | 0.811722 | 1 | REACTOME_DUAL_INCISION_REACTION_IN_TC_NER |
| 27 | 5 | 0.812902 | 1 | REACTOME_FGFR_LIGAND_BINDING_AND_ACTIVATION |
| 33 | 6 | 0.822822 | 1 | REACTOME_MRNA_3_END_PROCESSING |
| 94 | 21 | 0.825592 | 1 | REACTOME_CELL_SURFACE_INTERACTIONS_AT_THE_VASCULAR_WALL |
| 33 | 6 | 0.825872 | 1 | REACTOME_GLOBAL_GENOMIC_NER |
| 21 | 5 | 0.828262 | 1 | KEGG_BIOSYNTHESIS_OF_UNSATURATED_FATTY_ACIDS |
| 28 | 5 | 0.828382 | 1 | ST_TUMOR_NECROSIS_FACTOR_PATHWAY |
| 32 | 6 | 0.830262 | 1 | REACTOME_FORMATION_OF_FIBRIN_CLOT_CLOTTING_CASCADE |
| 43 | 8 | 0.835392 | 1 | KEGG_LYSINE_DEGRADATION |
| 78 | 8 | 0.836842 | 1 | REACTOME_RNA_POLYMERASE_I_PROMOTER_CLEARANCE |
| 23 | 6 | 0.838072 | 1 | BIOCARTA_RAC1_PATHWAY |
| 28 | 4 | 0.842202 | 1 | KEGG_ASTHMA |
| 29 | 6 | 0.842752 | 1 | REACTOME_INTRINSIC_PATHWAY_FOR_APOPTOSIS |
| 44 | 8 | 0.845242 | 1 | REACTOME_TRANSCRIPTION_COUPLED_NER |
| 118 | 23 | 0.848302 | 1 | KEGG_SPLICEOSOME |
| 38 | 8 | 0.849252 | 1 | KEGG_TRYPTOPHAN_METABOLISM |
| 29 | 6 | 0.852281 | 1 | BIOCARTA_TNFR1_PATHWAY |
| 46 | 8 | 0.852511 | 1 | KEGG_INTESTINAL_IMMUNE_NETWORK_FOR_IGA_PRODUCTION |
| 42 | 7 | 0.852631 | 1 | KEGG_TYROSINE_METABOLISM |
| 26 | 4 | 0.856621 | 1 | REACTOME_EXTENSION_OF_TELOMERES |
| 49 | 9 | 0.857151 | 1 | REACTOME_NUCLEOTIDE_EXCISION_REPAIR |
| 24 | 5 | 0.859121 | 1 | REACTOME_RNA_POLYMERASE_I_TRANSCRIPTION_INITIATION |
| 48 | 10 | 0.859821 | 1 | REACTOME_AMINO_ACID_AND_OLIGOPEPTIDE_SLC_TRANSPORTERS |
| 38 | 7 | 0.863831 | 1 | REACTOME_PI3K_CASCADE |
| 23 | 5 | 0.872081 | 1 | REACTOME_SIGNALING_BY_BMP |
| 30 | 5 | 0.873231 | 1 | REACTOME_TAT_MEDIATED_HIV1_ELONGATION_ARREST_AND_RECOVERY |
| 80 | 21 | 0.874331 | 1 | REACTOME_INTEGRIN_CELL_SURFACE_INTERACTIONS |
| 31 | 5 | 0.879141 | 1 | REACTOME_AMINO_ACID_TRANSPORT_ACROSS_THE_PLASMA_MEMBRANE |
| 23 | 4 | 0.880291 | 1 | REACTOME_SEMA4D_INDUCED_CELL_MIGRATION_AND_GROWTH_CONE_COLLAPSE |
| 61 | 17 | 0.882111 | 1 | REACTOME_CELL_DEATH_SIGNALLING_VIA_NRAGE_NRIF_AND_NADE |
| 36 | 11 | 0.885431 | 1 | BIOCARTA_AGR_PATHWAY |
| 94 | 22 | 0.886581 | 1 | REACTOME_INORGANIC_CATION_ANION_SLC_TRANSPORTERS |
| 25 | 4 | 0.887221 | 1 | REACTOME_ADP_SIGNALLING_THROUGH_P2Y_PURINOCEPTOR_1 |
| 86 | 17 | 0.887361 | 1 | KEGG_APOPTOSIS |
| 23 | 3 | 0.890301 | 1 | KEGG_PROXIMAL_TUBULE_BICARBONATE_RECLAMATION |
| 30 | 5 | 0.890481 | 1 | REACTOME_DNA_STRAND_ELONGATION |
| 36 | 9 | 0.890501 | 1 | BIOCARTA_PAR1_PATHWAY |
| 32 | 6 | 0.891501 | 1 | KEGG_ALANINE_ASPARTATE_AND_GLUTAMATE_METABOLISM |
| 31 | 4 | 0.894651 | 1 | KEGG_GLYCINE_SERINE_AND_THREONINE_METABOLISM |
| 20 | 3 | 0.896571 | 1 | REACTOME_LAGGING_STRAND_SYNTHESIS |
| 33 | 5 | 0.898261 | 1 | KEGG_PORPHYRIN_AND_CHLOROPHYLL_METABOLISM |
| 30 | 7 | 0.900011 | 1 | BIOCARTA_MYOSIN_PATHWAY |
| 34 | 6 | 0.903091 | 1 | KEGG_FRUCTOSE_AND_MANNOSE_METABOLISM |
| 21 | 5 | 0.903651 | 1 | BIOCARTA_CHEMICAL_PATHWAY |
| 44 | 7 | 0.906671 | 1 | KEGG_NUCLEOTIDE_EXCISION_REPAIR |
| 27 | 4 | 0.909841 | 1 | REACTOME_FRS2MEDIATED_CASCADE |
| 33 | 4 | 0.913541 | 1 | REACTOME_RNA_POLYMERASE_I_CHAIN_ELONGATION |
| 21 | 3 | 0.913881 | 1 | BIOCARTA_MITOCHONDRIA_PATHWAY |
| 34 | 6 | 0.918121 | 1 | KEGG_BUTANOATE_METABOLISM |
| 29 | 7 | 0.918921 | 1 | REACTOME_AMINE_COMPOUND_SLC_TRANSPORTERS |
| 32 | 6 | 0.918991 | 1 | REACTOME_GLUCAGON_SIGNALING_IN_METABOLIC_REGULATION |
| 68 | 8 | 0.919581 | 1 | REACTOME_IMMUNOREGULATORY_INTERACTIONS_BETWEEN_A_LYMPHOID_AND_A_NON_LYMPHOID_CELL |
| 26 | 6 | 0.920271 | 1 | KEGG_GLYCOSAMINOGLYCAN_BIOSYNTHESIS_HEPARAN_SULFATE |
| 61 | 16 | 0.920921 | 1 | REACTOME_REGULATION_OF_INSULIN_SECRETION_BY_GLUCAGON_LIKE_PEPTIDE_1 |
| 30 | 6 | 0.920961 | 1 | REACTOME_INHIBITION_OF_INSULIN_SECRETION_BY_ADRENALINE_NORADRENALINE |
| 27 | 6 | 0.923391 | 1 | REACTOME_NEUROTRANSMITTER_RELEASE_CYCLE |
| 59 | 12 | 0.923861 | 1 | REACTOME_PLATELET_ACTIVATION_TRIGGERS |
| 19 | 2 | 0.926931 | 1 | KEGG_PENTOSE_AND_GLUCURONATE_INTERCONVERSIONS |
| 21 | 3 | 0.931511 | 1 | REACTOME_RNA_POLYMERASE_I_TRANSCRIPTION_TERMINATION |
| 46 | 2 | 0.931941 | 1 | REACTOME_PACKAGING_OF_TELOMERE_ENDS |
| 26 | 5 | 0.936061 | 1 | BIOCARTA_CREB_PATHWAY |
| 46 | 10 | 0.936281 | 1 | KEGG_GLYCEROLIPID_METABOLISM |
| 26 | 3 | 0.936371 | 1 | KEGG_PENTOSE_PHOSPHATE_PATHWAY |
| 72 | 6 | 0.943281 | 1 | REACTOME_TELOMERE_MAINTENANCE |
| 41 | 6 | 0.945171 | 1 | REACTOME_HIV1_TRANSCRIPTION_ELONGATION |
| 47 | 13 | 0.948451 | 1 | REACTOME_NRAGE_SIGNALS_DEATH_THROUGH_JNK |
| 63 | 10 | 0.95233 | 1 | REACTOME_COSTIMULATION_BY_THE_CD28_FAMILY |
| 28 | 4 | 0.95504 | 1 | BIOCARTA_VEGF_PATHWAY |
| 20 | 5 | 0.95662 | 1 | REACTOME_REGULATION_OF_INSULIN_SECRETION_BY_FREE_FATTY_ACIDS |
| 23 | 3 | 0.95987 | 1 | KEGG_MISMATCH_REPAIR |
| 28 | 3 | 0.96357 | 1 | REACTOME_TOLL_LIKE_RECEPTOR_4_CASCADE |
| 24 | 2 | 0.96457 | 1 | REACTOME_ACTIVATED_TLR4_SIGNALLING |
| 22 | 6 | 0.97081 | 1 | REACTOME_REGULATION_OF_INSULIN_SECRETION_BY_ACETYLCHOLINE |
| 23 | 2 | 0.98047 | 1 | REACTOME_PHOSPHOLIPASE_CMEDIATED_CASCADE |
| 23 | 3 | 0.9814 | 1 | KEGG_PROTEIN_EXPORT |
| 23 | 4 | 0.98149 | 1 | REACTOME_COLLAGEN_MEDIATED_ACTIVATION_CASCADE |
| 27 | 4 | 0.98321 | 1 | REACTOME_CD28_CO_STIMULATION |
| 22 | 3 | 0.9838 | 1 | KEGG_BETA_ALANINE_METABOLISM |
| 23 | 2 | 0.98442 | 1 | BIOCARTA_CASPASE_PATHWAY |
| 43 | 5 | 0.98456 | 1 | KEGG_DRUG_METABOLISM_OTHER_ENZYMES |
| 42 | 5 | 0.98659 | 1 | REACTOME_METABOLISM_OF_VITAMINS_AND_COFACTORS |
| 52 | 3 | 0.99027 | 1 | REACTOME_CHEMOKINE_RECEPTORS_BIND_CHEMOKINES |
| 21 | 2 | 0.99031 | 1 | REACTOME_CTLA4_INHIBITORY_SIGNALING |
| 54 | 12 | 0.99054 | 1 | REACTOME_G_ALPHA_12_13_SIGNALLING_EVENTS |
| 26 | 2 | 0.99441 | 1 | KEGG_GLYCOSPHINGOLIPID_BIOSYNTHESIS_LACTO_AND_NEOLACTO_SERIES |
| 43 | 6 | 0.99673 | 1 | KEGG_VALINE_LEUCINE_AND_ISOLEUCINE_DEGRADATION |
| 114 | 26 | 0.99812 | 1 | REACTOME_RHO_GTPASE_CYCLE |
