# Supplementary material for: Whole-Genome Pathway Analysis on 132,497 Individuals Identifies Novel Gene-Sets Associated with Body Mass Index
Source: PLoS One. 2014 Jan 31;9(1):e78546. doi: 10.1371/journal.pone.0078546 (PMC3908858; doi:10.1371/journal.pone.0078546)
Supplement: Table S3 — INRICH Results for Discovery Set cutoff top 1%. (DOC) [file pone.0078546.s012.doc]

Table S3. Discovery INRICH results

INRICH Results for Discovery Set cutoff top 1%

| Target_Size | Int_No | Empirical_P | Corrected_P | Pathway |
| --- | --- | --- | --- | --- |
| 173 | 20 | 0.00381996 | 0.468506 | REACTOME_G_ALPHA_I_SIGNALLING_EVENTS |
| 108 | 15 | 0.00453995 | 0.531494 | REACTOME_CELL_CYCLE_CHECKPOINTS |
| 35 | 8 | 0.00555994 | 0.605879 | KEGG_PRION_DISEASES |
| 22 | 6 | 0.00841992 | 0.746051 | BIOCARTA_EIF4_PATHWAY |
| 36 | 7 | 0.00856991 | 0.75145 | REACTOME_ACTIVATION_OF_ATR_IN_RESPONSE_TO_REPLICATION_STRESS |
| 23 | 5 | 0.0107499 | 0.824235 | BIOCARTA_G2_PATHWAY |
| 21 | 5 | 0.0109999 | 0.832833 | REACTOME_ERK_MAPK_TARGETS |
| 58 | 9 | 0.0110799 | 0.835033 | REACTOME_SIGNALING_BY_WNT |
| 95 | 13 | 0.0154798 | 0.904419 | REACTOME_METABOLISM_OF_RNA |
| 41 | 8 | 0.0183098 | 0.934213 | KEGG_AMINOACYL_TRNA_BIOSYNTHESIS |
| 155 | 18 | 0.0196998 | 0.942012 | REACTOME_MITOTIC_M_M_G1_PHASES |
| 41 | 7 | 0.0208598 | 0.946611 | REACTOME_G2_M_CHECKPOINTS |
| 24 | 5 | 0.0232898 | 0.962807 | REACTOME_NUCLEAR_EVENTS_KINASE_AND_TRANSCRIPTION_FACTOR_ACTIVATION |
| 23 | 5 | 0.0262597 | 0.975405 | BIOCARTA_TPO_PATHWAY |
| 116 | 10 | 0.0275797 | 0.980004 | REACTOME_RNA_POLYMERASE_I_III_AND_MITOCHONDRIAL_TRANSCRIPTION |
| 99 | 15 | 0.0276697 | 0.980004 | REACTOME_TRKA_SIGNALLING_FROM_THE_PLASMA_MEMBRANE |
| 55 | 7 | 0.0279497 | 0.980604 | REACTOME_TRANSLATION_INITIATION_COMPLEX_FORMATION |
| 52 | 10 | 0.0288097 | 0.982404 | KEGG_ENDOMETRIAL_CANCER |
| 46 | 7 | 0.0294197 | 0.983803 | REACTOME_STABILIZATION_OF_P53 |
| 90 | 12 | 0.0307897 | 0.986203 | REACTOME_LATE_PHASE_OF_HIV_LIFE_CYCLE |
| 133 | 16 | 0.0313897 | 0.987403 | KEGG_UBIQUITIN_MEDIATED_PROTEOLYSIS |
| 120 | 14 | 0.0331697 | 0.988802 | KEGG_LYSOSOME |
| 22 | 4 | 0.0331197 | 0.988802 | REACTOME_MTOR_SIGNALLING |
| 104 | 13 | 0.0341497 | 0.989402 | REACTOME_DNA_REPAIR |
| 26 | 6 | 0.0346397 | 0.989802 | BIOCARTA_EDG1_PATHWAY |
| 27 | 5 | 0.0352096 | 0.991002 | BIOCARTA_GH_PATHWAY |
| 31 | 6 | 0.0352196 | 0.991002 | REACTOME_REV_MEDIATED_NUCLEAR_EXPORT_OF_HIV1_RNA |
| 51 | 10 | 0.0369396 | 0.992801 | KEGG_INOSITOL_PHOSPHATE_METABOLISM |
| 38 | 7 | 0.0405496 | 0.995401 | REACTOME_TRNA_AMINOACYLATION |
| 86 | 11 | 0.0415396 | 0.996201 | BIOCARTA_MAPK_PATHWAY |
| 73 | 13 | 0.0414096 | 0.996201 | KEGG_ADHERENS_JUNCTION |
| 77 | 11 | 0.0430696 | 0.996801 | KEGG_FC_EPSILON_RI_SIGNALING_PATHWAY |
| 62 | 9 | 0.0452595 | 0.997401 | REACTOME_STEROID_METABOLISM |
| 24 | 5 | 0.0469295 | 0.9982 | BIOCARTA_NTHI_PATHWAY |
| 56 | 8 | 0.0483095 | 0.999 | KEGG_ACUTE_MYELOID_LEUKEMIA |
| 54 | 8 | 0.0501895 | 0.9994 | BIOCARTA_PPARA_PATHWAY |
| 122 | 13 | 0.0543595 | 0.9994 | KEGG_CELL_CYCLE |
| 91 | 13 | 0.0523995 | 0.9994 | KEGG_FC_GAMMA_R_MEDIATED_PHAGOCYTOSIS |
| 72 | 13 | 0.0528295 | 0.9994 | KEGG_PHOSPHATIDYLINOSITOL_SIGNALING_SYSTEM |
| 46 | 8 | 0.0563394 | 0.9994 | KEGG_TYPE_II_DIABETES_MELLITUS |
| 74 | 9 | 0.0568394 | 0.9994 | KEGG_VEGF_SIGNALING_PATHWAY |
| 57 | 7 | 0.0536695 | 0.9994 | REACTOME_AUTODEGRADATION_OF_CDH1_BY_CDH1_APC |
| 78 | 6 | 0.0533495 | 0.9994 | REACTOME_RNA_POLYMERASE_I_PROMOTER_CLEARANCE |
| 49 | 7 | 0.0565194 | 0.9994 | REACTOME_SNRNP_ASSEMBLY |
| 33 | 5 | 0.0591794 | 0.9996 | BIOCARTA_MPR_PATHWAY |
| 99 | 14 | 0.0580594 | 0.9996 | KEGG_GNRH_SIGNALING_PATHWAY |
| 22 | 4 | 0.0650793 | 0.9998 | BIOCARTA_MTOR_PATHWAY |
| 27 | 5 | 0.0691493 | 0.9998 | BIOCARTA_PYK2_PATHWAY |
| 154 | 18 | 0.0756792 | 0.9998 | KEGG_ALZHEIMERS_DISEASE |
| 90 | 14 | 0.0657093 | 0.9998 | KEGG_DILATED_CARDIOMYOPATHY |
| 126 | 15 | 0.0690993 | 0.9998 | KEGG_NEUROTROPHIN_SIGNALING_PATHWAY |
| 44 | 6 | 0.0624294 | 0.9998 | KEGG_PROTEASOME |
| 36 | 6 | 0.0684393 | 0.9998 | KEGG_SPHINGOLIPID_METABOLISM |
| 103 | 11 | 0.0767592 | 0.9998 | REACTOME_INNATE_IMMUNITY_SIGNALING |
| 41 | 6 | 0.0700793 | 0.9998 | REACTOME_MAP_KINASES_ACTIVATION_IN_TLR_CASCADE |
| 30 | 5 | 0.0684793 | 0.9998 | REACTOME_MAPK_TARGETS_NUCLEAR_EVENTS_MEDIATED_BY_MAP_KINASES |
| 90 | 11 | 0.0632994 | 0.9998 | REACTOME_MITOTIC_PROMETAPHASE |
| 70 | 8 | 0.0740293 | 0.9998 | REACTOME_REGULATION_OF_APC_ACTIVATORS_BETWEEN_G1_S_AND_EARLY_ANAPHASE |
| 29 | 5 | 0.0800292 | 0.9998 | REACTOME_REGULATION_OF_GLUCOKINASE_BY_GLUCOKINASE_REGULATORY_PROTEIN |
| 23 | 5 | 0.0722893 | 0.9998 | ST_MYOCYTE_AD_PATHWAY |
| 20 | 3 | 0.112989 | 1 | BIOCARTA_ACTINY_PATHWAY |
| 36 | 5 | 0.525525 | 1 | BIOCARTA_AGR_PATHWAY |
| 22 | 2 | 0.559904 | 1 | BIOCARTA_AKT_PATHWAY |
| 37 | 4 | 0.372956 | 1 | BIOCARTA_ALK_PATHWAY |
| 31 | 5 | 0.123039 | 1 | BIOCARTA_AT1R_PATHWAY |
| 20 | 2 | 0.479585 | 1 | BIOCARTA_ATM_PATHWAY |
| 21 | 4 | 0.0830992 | 1 | BIOCARTA_ATRBRCA_PATHWAY |
| 26 | 3 | 0.400806 | 1 | BIOCARTA_BAD_PATHWAY |
| 33 | 3 | 0.501855 | 1 | BIOCARTA_BCR_PATHWAY |
| 41 | 5 | 0.201738 | 1 | BIOCARTA_BIOPEPTIDES_PATHWAY |
| 35 | 3 | 0.577484 | 1 | BIOCARTA_CARM_ER_PATHWAY |
| 22 | 4 | 0.256727 | 1 | BIOCARTA_CCR3_PATHWAY |
| 22 | 3 | 0.245798 | 1 | BIOCARTA_CERAMIDE_PATHWAY |
| 21 | 2 | 0.728903 | 1 | BIOCARTA_CHEMICAL_PATHWAY |
| 42 | 4 | 0.360266 | 1 | BIOCARTA_CHREBP2_PATHWAY |
| 26 | 4 | 0.181478 | 1 | BIOCARTA_CREB_PATHWAY |
| 22 | 2 | 0.400106 | 1 | BIOCARTA_CSK_PATHWAY |
| 23 | 4 | 0.177418 | 1 | BIOCARTA_CXCR4_PATHWAY |
| 21 | 2 | 0.427906 | 1 | BIOCARTA_CYTOKINE_PATHWAY |
| 24 | 2 | 0.611224 | 1 | BIOCARTA_ECM_PATHWAY |
| 30 | 4 | 0.205758 | 1 | BIOCARTA_EGF_PATHWAY |
| 28 | 3 | 0.365156 | 1 | BIOCARTA_ERK_PATHWAY |
| 37 | 3 | 0.526685 | 1 | BIOCARTA_FCER1_PATHWAY |
| 35 | 4 | 0.293057 | 1 | BIOCARTA_FMLP_PATHWAY |
| 27 | 2 | 0.689693 | 1 | BIOCARTA_G1_PATHWAY |
| 23 | 2 | 0.608984 | 1 | BIOCARTA_GLEEVEC_PATHWAY |
| 33 | 3 | 0.478015 | 1 | BIOCARTA_GPCR_PATHWAY |
| 27 | 2 | 0.542925 | 1 | BIOCARTA_GSK3_PATHWAY |
| 28 | 2 | 0.670093 | 1 | BIOCARTA_HDAC_PATHWAY |
| 22 | 4 | 0.166868 | 1 | BIOCARTA_HER2_PATHWAY |
| 56 | 4 | 0.601904 | 1 | BIOCARTA_HIVNEF_PATHWAY |
| 21 | 2 | 0.410756 | 1 | BIOCARTA_IGF1_PATHWAY |
| 23 | 3 | 0.312327 | 1 | BIOCARTA_IGF1R_PATHWAY |
| 21 | 2 | 0.400896 | 1 | BIOCARTA_IL12_PATHWAY |
| 32 | 3 | 0.450815 | 1 | BIOCARTA_IL1R_PATHWAY |
| 38 | 3 | 0.573014 | 1 | BIOCARTA_IL2RB_PATHWAY |
| 29 | 4 | 0.097999 | 1 | BIOCARTA_INFLAM_PATHWAY |
| 22 | 2 | 0.372286 | 1 | BIOCARTA_INSULIN_PATHWAY |
| 38 | 5 | 0.191908 | 1 | BIOCARTA_INTEGRIN_PATHWAY |
| 22 | 3 | 0.190498 | 1 | BIOCARTA_INTRINSIC_PATHWAY |
| 45 | 6 | 0.315437 | 1 | BIOCARTA_KERATINOCYTE_PATHWAY |
| 25 | 2 | 0.592354 | 1 | BIOCARTA_MCALPAIN_PATHWAY |
| 37 | 6 | 0.096149 | 1 | BIOCARTA_MET_PATHWAY |
| 30 | 2 | 0.804962 | 1 | BIOCARTA_MYOSIN_PATHWAY |
| 53 | 3 | 0.744373 | 1 | BIOCARTA_NFAT_PATHWAY |
| 20 | 2 | 0.500875 | 1 | BIOCARTA_NKCELLS_PATHWAY |
| 20 | 2 | 0.713213 | 1 | BIOCARTA_NOS1_PATHWAY |
| 39 | 5 | 0.172068 | 1 | BIOCARTA_P38MAPK_PATHWAY |
| 22 | 3 | 0.260017 | 1 | BIOCARTA_P53HYPOXIA_PATHWAY |
| 36 | 2 | 0.886631 | 1 | BIOCARTA_PAR1_PATHWAY |
| 31 | 4 | 0.176488 | 1 | BIOCARTA_PDGF_PATHWAY |
| 22 | 3 | 0.335617 | 1 | BIOCARTA_PGC1A_PATHWAY |
| 23 | 3 | 0.384216 | 1 | BIOCARTA_PTDINS_PATHWAY |
| 26 | 2 | 0.521635 | 1 | BIOCARTA_RACCYCD_PATHWAY |
| 23 | 2 | 0.598544 | 1 | BIOCARTA_RAS_PATHWAY |
| 32 | 4 | 0.192618 | 1 | BIOCARTA_RHO_PATHWAY |
| 21 | 4 | 0.206898 | 1 | BIOCARTA_SPPA_PATHWAY |
| 25 | 2 | 0.553184 | 1 | BIOCARTA_STRESS_PATHWAY |
| 43 | 4 | 0.386386 | 1 | BIOCARTA_TCR_PATHWAY |
| 21 | 3 | 0.325217 | 1 | BIOCARTA_TFF_PATHWAY |
| 29 | 2 | 0.651173 | 1 | BIOCARTA_TNFR1_PATHWAY |
| 35 | 2 | 0.689863 | 1 | BIOCARTA_TOLL_PATHWAY |
| 28 | 3 | 0.332167 | 1 | BIOCARTA_VEGF_PATHWAY |
| 25 | 4 | 0.123399 | 1 | BIOCARTA_WNT_PATHWAY |
| 66 | 5 | 0.588594 | 1 | KEGG_ADIPOCYTOKINE_SIGNALING_PATHWAY |
| 41 | 4 | 0.355026 | 1 | KEGG_ALDOSTERONE_REGULATED_SODIUM_REABSORPTION |
| 35 | 4 | 0.256877 | 1 | KEGG_ALLOGRAFT_REJECTION |
| 44 | 4 | 0.493835 | 1 | KEGG_AMINO_SUGAR_AND_NUCLEOTIDE_SUGAR_METABOLISM |
| 53 | 7 | 0.248688 | 1 | KEGG_AMYOTROPHIC_LATERAL_SCLEROSIS_ALS |
| 81 | 4 | 0.686033 | 1 | KEGG_ANTIGEN_PROCESSING_AND_PRESENTATION |
| 86 | 5 | 0.762012 | 1 | KEGG_APOPTOSIS |
| 56 | 3 | 0.594434 | 1 | KEGG_ARACHIDONIC_ACID_METABOLISM |
| 53 | 4 | 0.358486 | 1 | KEGG_ARGININE_AND_PROLINE_METABOLISM |
| 74 | 13 | 0.120279 | 1 | KEGG_ARRHYTHMOGENIC_RIGHT_VENTRICULAR_CARDIOMYOPATHY_ARVC |
| 28 | 2 | 0.573334 | 1 | KEGG_ASTHMA |
| 50 | 3 | 0.691153 | 1 | KEGG_AUTOIMMUNE_THYROID_DISEASE |
| 128 | 19 | 0.0899091 | 1 | KEGG_AXON_GUIDANCE |
| 74 | 6 | 0.514325 | 1 | KEGG_B_CELL_RECEPTOR_SIGNALING_PATHWAY |
| 55 | 6 | 0.187078 | 1 | KEGG_BASAL_CELL_CARCINOMA |
| 35 | 2 | 0.622624 | 1 | KEGG_BASAL_TRANSCRIPTION_FACTORS |
| 33 | 3 | 0.286047 | 1 | KEGG_BASE_EXCISION_REPAIR |
| 22 | 2 | 0.533505 | 1 | KEGG_BETA_ALANINE_METABOLISM |
| 42 | 5 | 0.305917 | 1 | KEGG_BLADDER_CANCER |
| 34 | 2 | 0.729373 | 1 | KEGG_BUTANOATE_METABOLISM |
| 175 | 22 | 0.150628 | 1 | KEGG_CALCIUM_SIGNALING_PATHWAY |
| 70 | 9 | 0.096949 | 1 | KEGG_CARDIAC_MUSCLE_CONTRACTION |
| 131 | 12 | 0.668613 | 1 | KEGG_CELL_ADHESION_MOLECULES_CAMS |
| 183 | 15 | 0.264097 | 1 | KEGG_CHEMOKINE_SIGNALING_PATHWAY |
| 72 | 7 | 0.389536 | 1 | KEGG_CHRONIC_MYELOID_LEUKEMIA |
| 30 | 2 | 0.623114 | 1 | KEGG_CITRATE_CYCLE_TCA_CYCLE |
| 62 | 8 | 0.220748 | 1 | KEGG_COLORECTAL_CANCER |
| 68 | 5 | 0.588044 | 1 | KEGG_COMPLEMENT_AND_COAGULATION_CASCADES |
| 34 | 3 | 0.429976 | 1 | KEGG_CYSTEINE_AND_METHIONINE_METABOLISM |
| 36 | 2 | 0.687313 | 1 | KEGG_DNA_REPLICATION |
| 24 | 3 | 0.411236 | 1 | KEGG_DORSO_VENTRAL_AXIS_FORMATION |
| 43 | 2 | 0.769502 | 1 | KEGG_DRUG_METABOLISM_OTHER_ENZYMES |
| 84 | 8 | 0.644004 | 1 | KEGG_ECM_RECEPTOR_INTERACTION |
| 167 | 18 | 0.123979 | 1 | KEGG_ENDOCYTOSIS |
| 66 | 3 | 0.897091 | 1 | KEGG_EPITHELIAL_CELL_SIGNALING_IN_HELICOBACTER_PYLORI_INFECTION |
| 85 | 9 | 0.520625 | 1 | KEGG_ERBB_SIGNALING_PATHWAY |
| 32 | 2 | 0.652403 | 1 | KEGG_ETHER_LIPID_METABOLISM |
| 195 | 18 | 0.536075 | 1 | KEGG_FOCAL_ADHESION |
| 34 | 2 | 0.716433 | 1 | KEGG_FRUCTOSE_AND_MANNOSE_METABOLISM |
| 88 | 11 | 0.246818 | 1 | KEGG_GAP_JUNCTION |
| 63 | 6 | 0.408146 | 1 | KEGG_GLIOMA |
| 49 | 2 | 0.701763 | 1 | KEGG_GLUTATHIONE_METABOLISM |
| 46 | 2 | 0.908061 | 1 | KEGG_GLYCEROLIPID_METABOLISM |
| 73 | 5 | 0.627644 | 1 | KEGG_GLYCEROPHOSPHOLIPID_METABOLISM |
| 62 | 4 | 0.469925 | 1 | KEGG_GLYCOLYSIS_GLUCONEOGENESIS |
| 20 | 2 | 0.698423 | 1 | KEGG_GLYCOSAMINOGLYCAN_BIOSYNTHESIS_CHONDROITIN_SULFATE |
| 26 | 2 | 0.746513 | 1 | KEGG_GLYCOSAMINOGLYCAN_BIOSYNTHESIS_HEPARAN_SULFATE |
| 25 | 3 | 0.201788 | 1 | KEGG_GLYCOSYLPHOSPHATIDYLINOSITOL_GPI_ANCHOR_BIOSYNTHESIS |
| 37 | 3 | 0.510865 | 1 | KEGG_GRAFT_VERSUS_HOST_DISEASE |
| 56 | 4 | 0.595174 | 1 | KEGG_HEDGEHOG_SIGNALING_PATHWAY |
| 85 | 8 | 0.177878 | 1 | KEGG_HEMATOPOIETIC_CELL_LINEAGE |
| 29 | 3 | 0.309827 | 1 | KEGG_HISTIDINE_METABOLISM |
| 28 | 5 | 0.106969 | 1 | KEGG_HOMOLOGOUS_RECOMBINATION |
| 169 | 15 | 0.255687 | 1 | KEGG_HUNTINGTONS_DISEASE |
| 83 | 11 | 0.214918 | 1 | KEGG_HYPERTROPHIC_CARDIOMYOPATHY_HCM |
| 134 | 9 | 0.571654 | 1 | KEGG_INSULIN_SIGNALING_PATHWAY |
| 46 | 2 | 0.855441 | 1 | KEGG_INTESTINAL_IMMUNE_NETWORK_FOR_IGA_PRODUCTION |
| 154 | 6 | 0.810772 | 1 | KEGG_JAK_STAT_SIGNALING_PATHWAY |
| 66 | 5 | 0.395626 | 1 | KEGG_LEISHMANIA_INFECTION |
| 112 | 10 | 0.416676 | 1 | KEGG_LEUKOCYTE_TRANSENDOTHELIAL_MIGRATION |
| 28 | 2 | 0.382926 | 1 | KEGG_LINOLEIC_ACID_METABOLISM |
| 68 | 11 | 0.121679 | 1 | KEGG_LONG_TERM_DEPRESSION |
| 69 | 11 | 0.166368 | 1 | KEGG_LONG_TERM_POTENTIATION |
| 43 | 3 | 0.553244 | 1 | KEGG_LYSINE_DEGRADATION |
| 24 | 2 | 0.518955 | 1 | KEGG_MATURITY_ONSET_DIABETES_OF_THE_YOUNG |
| 100 | 11 | 0.169738 | 1 | KEGG_MELANOGENESIS |
| 71 | 6 | 0.591754 | 1 | KEGG_MELANOMA |
| 48 | 5 | 0.232788 | 1 | KEGG_MTOR_SIGNALING_PATHWAY |
| 45 | 2 | 0.902611 | 1 | KEGG_N_GLYCAN_BIOSYNTHESIS |
| 131 | 8 | 0.521915 | 1 | KEGG_NATURAL_KILLER_CELL_MEDIATED_CYTOTOXICITY |
| 23 | 2 | 0.543155 | 1 | KEGG_NICOTINATE_AND_NICOTINAMIDE_METABOLISM |
| 58 | 4 | 0.451485 | 1 | KEGG_NOD_LIKE_RECEPTOR_SIGNALING_PATHWAY |
| 53 | 9 | 0.0901891 | 1 | KEGG_NON_SMALL_CELL_LUNG_CANCER |
| 46 | 2 | 0.852881 | 1 | KEGG_NOTCH_SIGNALING_PATHWAY |
| 44 | 3 | 0.531275 | 1 | KEGG_NUCLEOTIDE_EXCISION_REPAIR |
| 29 | 4 | 0.410616 | 1 | KEGG_O_GLYCAN_BIOSYNTHESIS |
| 111 | 11 | 0.185578 | 1 | KEGG_OOCYTE_MEIOSIS |
| 113 | 8 | 0.386966 | 1 | KEGG_OXIDATIVE_PHOSPHORYLATION |
| 66 | 6 | 0.394386 | 1 | KEGG_P53_SIGNALING_PATHWAY |
| 70 | 6 | 0.570544 | 1 | KEGG_PANCREATIC_CANCER |
| 110 | 9 | 0.317977 | 1 | KEGG_PARKINSONS_DISEASE |
| 56 | 3 | 0.644874 | 1 | KEGG_PATHOGENIC_ESCHERICHIA_COLI_INFECTION |
| 26 | 2 | 0.458465 | 1 | KEGG_PENTOSE_PHOSPHATE_PATHWAY |
| 76 | 4 | 0.830492 | 1 | KEGG_PEROXISOME |
| 33 | 2 | 0.645574 | 1 | KEGG_PORPHYRIN_AND_CHLOROPHYLL_METABOLISM |
| 68 | 7 | 0.277717 | 1 | KEGG_PPAR_SIGNALING_PATHWAY |
| 35 | 3 | 0.376116 | 1 | KEGG_PRIMARY_IMMUNODEFICIENCY |
| 84 | 8 | 0.229738 | 1 | KEGG_PROGESTERONE_MEDIATED_OOCYTE_MATURATION |
| 32 | 4 | 0.320627 | 1 | KEGG_PROPANOATE_METABOLISM |
| 88 | 7 | 0.588104 | 1 | KEGG_PROSTATE_CANCER |
| 23 | 2 | 0.598044 | 1 | KEGG_PROTEIN_EXPORT |
| 155 | 18 | 0.116089 | 1 | KEGG_PURINE_METABOLISM |
| 95 | 10 | 0.150668 | 1 | KEGG_PYRIMIDINE_METABOLISM |
| 40 | 5 | 0.164958 | 1 | KEGG_PYRUVATE_METABOLISM |
| 70 | 8 | 0.205258 | 1 | KEGG_RENAL_CELL_CARCINOMA |
| 86 | 5 | 0.483865 | 1 | KEGG_RIBOSOME |
| 68 | 3 | 0.661663 | 1 | KEGG_RIG_I_LIKE_RECEPTOR_SIGNALING_PATHWAY |
| 55 | 6 | 0.206348 | 1 | KEGG_RNA_DEGRADATION |
| 29 | 3 | 0.314507 | 1 | KEGG_RNA_POLYMERASE |
| 26 | 3 | 0.335377 | 1 | KEGG_SELENOAMINO_ACID_METABOLISM |
| 83 | 8 | 0.530985 | 1 | KEGG_SMALL_CELL_LUNG_CANCER |
| 118 | 6 | 0.829382 | 1 | KEGG_SPLICEOSOME |
| 42 | 2 | 0.831702 | 1 | KEGG_STARCH_AND_SUCROSE_METABOLISM |
| 47 | 3 | 0.591944 | 1 | KEGG_STEROID_HORMONE_BIOSYNTHESIS |
| 124 | 5 | 0.652893 | 1 | KEGG_SYSTEMIC_LUPUS_ERYTHEMATOSUS |
| 108 | 11 | 0.164158 | 1 | KEGG_T_CELL_RECEPTOR_SIGNALING_PATHWAY |
| 85 | 10 | 0.128699 | 1 | KEGG_TGF_BETA_SIGNALING_PATHWAY |
| 29 | 4 | 0.233428 | 1 | KEGG_THYROID_CANCER |
| 128 | 15 | 0.240508 | 1 | KEGG_TIGHT_JUNCTION |
| 100 | 7 | 0.290097 | 1 | KEGG_TOLL_LIKE_RECEPTOR_SIGNALING_PATHWAY |
| 38 | 4 | 0.291787 | 1 | KEGG_TRYPTOPHAN_METABOLISM |
| 41 | 5 | 0.255067 | 1 | KEGG_TYPE_I_DIABETES_MELLITUS |
| 42 | 3 | 0.522115 | 1 | KEGG_TYROSINE_METABOLISM |
| 43 | 2 | 0.890371 | 1 | KEGG_VALINE_LEUCINE_AND_ISOLEUCINE_DEGRADATION |
| 113 | 16 | 0.126139 | 1 | KEGG_VASCULAR_SMOOTH_MUSCLE_CONTRACTION |
| 44 | 5 | 0.198078 | 1 | KEGG_VASOPRESSIN_REGULATED_WATER_REABSORPTION |
| 53 | 6 | 0.216958 | 1 | KEGG_VIBRIO_CHOLERAE_INFECTION |
| 68 | 7 | 0.343197 | 1 | KEGG_VIRAL_MYOCARDITIS |
| 149 | 13 | 0.454295 | 1 | KEGG_WNT_SIGNALING_PATHWAY |
| 23 | 3 | 0.263497 | 1 | REACTOME_ABORTIVE_ELONGATION_OF_HIV1_TRANSCRIPT_IN_THE_ABSENCE_OF_TAT |
| 32 | 4 | 0.411816 | 1 | REACTOME_ACTIVATION_OF_KAINATE_RECEPTORS_UPON_GLUTAMATE_BINDING |
| 36 | 3 | 0.823592 | 1 | REACTOME_ACTIVATION_OF_NMDA_RECEPTOR_UPON_GLUTAMATE_BINDING_AND_POSTSYNAPTIC_EVENTS |
| 29 | 2 | 0.572614 | 1 | REACTOME_ACTIVATION_OF_THE_PRE_REPLICATIVE_COMPLEX |
| 29 | 5 | 0.406356 | 1 | REACTOME_ADHERENS_JUNCTIONS_INTERACTIONS |
| 25 | 2 | 0.491325 | 1 | REACTOME_ADP_SIGNALLING_THROUGH_P2Y_PURINOCEPTOR_1 |
| 29 | 3 | 0.511825 | 1 | REACTOME_AMINE_COMPOUND_SLC_TRANSPORTERS |
| 41 | 5 | 0.139029 | 1 | REACTOME_AMINE_LIGAND_BINDING_RECEPTORS |
| 48 | 3 | 0.639344 | 1 | REACTOME_AMINO_ACID_AND_OLIGOPEPTIDE_SLC_TRANSPORTERS |
| 128 | 9 | 0.622124 | 1 | REACTOME_APOPTOSIS |
| 47 | 2 | 0.875871 | 1 | REACTOME_APOPTOTIC_EXECUTION_PHASE |
| 28 | 5 | 0.0840692 | 1 | REACTOME_ASSOCIATION_OF_TRIC_CCT_WITH_TARGET_PROTEINS_DURING_BIOSYNTHESIS |
| 157 | 19 | 0.219598 | 1 | REACTOME_AXON_GUIDANCE |
| 119 | 6 | 0.679423 | 1 | REACTOME_BIOLOGICAL_OXIDATIONS |
| 25 | 3 | 0.448586 | 1 | REACTOME_CAM_PATHWAY |
| 62 | 7 | 0.0905191 | 1 | REACTOME_CDC20_PHOSPHO_APC_MEDIATED_DEGRADATION_OF_CYCLIN_A |
| 52 | 6 | 0.112739 | 1 | REACTOME_CDT1_ASSOCIATION_WITH_THE_CDC6_ORC_ORIGIN_COMPLEX |
| 59 | 8 | 0.276057 | 1 | REACTOME_CELL_CELL_ADHESION_SYSTEMS |
| 61 | 2 | 0.99114 | 1 | REACTOME_CELL_DEATH_SIGNALLING_VIA_NRAGE_NRIF_AND_NADE |
| 83 | 11 | 0.192018 | 1 | REACTOME_CELL_JUNCTION_ORGANIZATION |
| 94 | 3 | 0.97692 | 1 | REACTOME_CELL_SURFACE_INTERACTIONS_AT_THE_VASCULAR_WALL |
| 67 | 5 | 0.439036 | 1 | REACTOME_CENTROSOME_MATURATION |
| 49 | 5 | 0.170068 | 1 | REACTOME_CHAPERONIN_MEDIATED_PROTEIN_FOLDING |
| 21 | 3 | 0.115799 | 1 | REACTOME_CHOLESTEROL_BIOSYNTHESIS |
| 83 | 2 | 0.96577 | 1 | REACTOME_CLASS_B2_SECRETIN_FAMILY_RECEPTORS |
| 61 | 5 | 0.523145 | 1 | REACTOME_CLATHRIN_DERIVED_VESICLE_BUDDING |
| 20 | 3 | 0.158618 | 1 | REACTOME_COMPLEMENT_CASCADE |
| 63 | 2 | 0.943461 | 1 | REACTOME_COSTIMULATION_BY_THE_CD28_FAMILY |
| 58 | 6 | 0.175888 | 1 | REACTOME_CYCLIN_E_ASSOCIATED_EVENTS_DURING_G1_S_TRANSITION_ |
| 49 | 4 | 0.367736 | 1 | REACTOME_CYTOCHROME_P450_ARRANGED_BY_SUBSTRATE_TYPE |
| 21 | 4 | 0.0911091 | 1 | REACTOME_CYTOSOLIC_TRNA_AMINOACYLATION |
| 26 | 3 | 0.400846 | 1 | REACTOME_DARPP32_EVENTS |
| 22 | 4 | 0.0949091 | 1 | REACTOME_DEADENYLATION_OF_MRNA |
| 75 | 7 | 0.201128 | 1 | REACTOME_DNA_REPLICATION_PRE_INITIATION |
| 30 | 3 | 0.378366 | 1 | REACTOME_DNA_STRAND_ELONGATION |
| 21 | 3 | 0.272867 | 1 | REACTOME_DOUBLE_STRAND_BREAK_REPAIR |
| 35 | 5 | 0.0925291 | 1 | REACTOME_DOWN_STREAM_SIGNAL_TRANSDUCTION |
| 43 | 3 | 0.564234 | 1 | REACTOME_DOWNSTREAM_SIGNALING_OF_ACTIVATED_FGFR |
| 41 | 2 | 0.810892 | 1 | REACTOME_DOWNSTREAM_TCR_SIGNALING |
| 20 | 2 | 0.403016 | 1 | REACTOME_DUAL_INCISION_REACTION_IN_GG_NER |
| 28 | 3 | 0.343897 | 1 | REACTOME_DUAL_INCISION_REACTION_IN_TC_NER |
| 31 | 2 | 0.699763 | 1 | REACTOME_E2F_MEDIATED_REGULATION_OF_DNA_REPLICATION |
| 63 | 3 | 0.697073 | 1 | REACTOME_ELECTRON_TRANSPORT_CHAIN |
| 130 | 7 | 0.776402 | 1 | REACTOME_ELONGATION_AND_PROCESSING_OF_CAPPED_TRANSCRIPTS |
| 148 | 9 | 0.663273 | 1 | REACTOME_FORMATION_AND_MATURATION_OF_MRNA_TRANSCRIPT |
| 94 | 5 | 0.579014 | 1 | REACTOME_FORMATION_OF_A_POOL_OF_FREE_40S_SUBUNITS |
| 32 | 3 | 0.415146 | 1 | REACTOME_FORMATION_OF_FIBRIN_CLOT_CLOTTING_CASCADE |
| 184 | 15 | 0.604634 | 1 | REACTOME_FORMATION_OF_PLATELET_PLUG |
| 32 | 4 | 0.205338 | 1 | REACTOME_FORMATION_OF_THE_EARLY_ELONGATION_COMPLEX |
| 48 | 4 | 0.256087 | 1 | REACTOME_FORMATION_OF_THE_TERNARY_COMPLEX_AND_SUBSEQUENTLY_THE_43S_COMPLEX |
| 23 | 2 | 0.656413 | 1 | REACTOME_FURTHER_PLATELET_RELEASATE |
| 153 | 10 | 0.818972 | 1 | REACTOME_G_ALPHA_Q_SIGNALLING_EVENTS |
| 122 | 9 | 0.642774 | 1 | REACTOME_G_ALPHA_S_SIGNALLING_EVENTS |
| 20 | 3 | 0.261987 | 1 | REACTOME_G_BETA_GAMMA_SIGNALLING_THROUGH_PLC_BETA |
| 28 | 3 | 0.239488 | 1 | REACTOME_G_PROTEIN_ACTIVATION |
| 28 | 3 | 0.382176 | 1 | REACTOME_G_PROTEIN_BETA_GAMMA_SIGNALLING |
| 100 | 9 | 0.224298 | 1 | REACTOME_G1_S_TRANSITION |
| 79 | 7 | 0.227638 | 1 | REACTOME_G2_M_TRANSITION |
| 28 | 2 | 0.425586 | 1 | REACTOME_GAP_JUNCTION_TRAFFICKING |
| 32 | 3 | 0.288017 | 1 | REACTOME_GENERATION_OF_SECOND_MESSENGER_MOLECULES |
| 35 | 5 | 0.127629 | 1 | REACTOME_GENERIC_TRANSCRIPTION_PATHWAY |
| 36 | 2 | 0.798392 | 1 | REACTOME_GENES_INVOLVED_IN_APOPTOTIC_CLEAVAGE_OF_CELLULAR_PROTEINS |
| 33 | 3 | 0.377706 | 1 | REACTOME_GLOBAL_GENOMIC_NER |
| 32 | 4 | 0.204708 | 1 | REACTOME_GLUCAGON_SIGNALING_IN_METABOLIC_REGULATION |
| 32 | 2 | 0.560824 | 1 | REACTOME_GLUCAGON_TYPE_LIGAND_RECEPTORS |
| 31 | 2 | 0.468695 | 1 | REACTOME_GLUCONEOGENESIS |
| 82 | 7 | 0.473675 | 1 | REACTOME_GLUCOSE_AND_OTHER_SUGAR_SLC_TRANSPORTERS |
| 56 | 4 | 0.392536 | 1 | REACTOME_GLUCOSE_METABOLISM |
| 147 | 9 | 0.647624 | 1 | REACTOME_GLUCOSE_REGULATION_OF_INSULIN_SECRETION |
| 38 | 5 | 0.201298 | 1 | REACTOME_GLUCOSE_TRANSPORT |
| 54 | 4 | 0.637644 | 1 | REACTOME_GOLGI_ASSOCIATED_VESICLE_BIOGENESIS |
| 25 | 4 | 0.117769 | 1 | REACTOME_GS_ALPHA_MEDIATED_EVENTS_IN_GLUCAGON_SIGNALLING |
| 105 | 9 | 0.112779 | 1 | REACTOME_GTP_HYDROLYSIS_AND_JOINING_OF_THE_60S_RIBOSOMAL_SUBUNIT |
| 183 | 18 | 0.158968 | 1 | REACTOME_HIV_INFECTION |
| 103 | 12 | 0.0940491 | 1 | REACTOME_HIV_LIFE_CYCLE |
| 41 | 4 | 0.344137 | 1 | REACTOME_HIV1_TRANSCRIPTION_ELONGATION |
| 39 | 5 | 0.107979 | 1 | REACTOME_HIV1_TRANSCRIPTION_INITIATION |
| 52 | 5 | 0.448746 | 1 | REACTOME_HORMONE_BIOSYNTHESIS |
| 120 | 14 | 0.0929391 | 1 | REACTOME_HOST_INTERACTIONS_OF_HIV_FACTORS |
| 68 | 3 | 0.683203 | 1 | REACTOME_IMMUNOREGULATORY_INTERACTIONS_BETWEEN_A_LYMPHOID_AND_A_NON_LYMPHOID_CELL |
| 137 | 13 | 0.0947391 | 1 | REACTOME_INFLUENZA_LIFE_CYCLE |
| 100 | 7 | 0.333897 | 1 | REACTOME_INFLUENZA_VIRAL_RNA_TRANSCRIPTION_AND_REPLICATION |
| 30 | 3 | 0.411706 | 1 | REACTOME_INHIBITION_OF_INSULIN_SECRETION_BY_ADRENALINE_NORADRENALINE |
| 94 | 6 | 0.808392 | 1 | REACTOME_INORGANIC_CATION_ANION_SLC_TRANSPORTERS |
| 129 | 7 | 0.731813 | 1 | REACTOME_INSULIN_SYNTHESIS_AND_SECRETION |
| 23 | 3 | 0.317737 | 1 | REACTOME_INTEGRIN_ALPHAIIBBETA3_SIGNALING |
| 80 | 7 | 0.550714 | 1 | REACTOME_INTEGRIN_CELL_SURFACE_INTERACTIONS |
| 74 | 8 | 0.0902391 | 1 | REACTOME_IRS_RELATED_EVENTS |
| 59 | 4 | 0.567014 | 1 | REACTOME_LOSS_OF_NLP_FROM_MITOTIC_CENTROSOMES |
| 24 | 2 | 0.619394 | 1 | REACTOME_LYSOSOME_VESICLE_BIOGENESIS |
| 61 | 7 | 0.0884691 | 1 | REACTOME_M_G1_TRANSITION |
| 78 | 5 | 0.717503 | 1 | REACTOME_MEMBRANE_TRAFFICKING |
| 70 | 7 | 0.266237 | 1 | REACTOME_METABLISM_OF_NUCLEOTIDES |
| 161 | 14 | 0.209868 | 1 | REACTOME_METABOLISM_OF_AMINO_ACIDS |
| 27 | 3 | 0.429096 | 1 | REACTOME_METABOLISM_OF_BILE_ACIDS_AND_BILE_SALTS |
| 117 | 10 | 0.173668 | 1 | REACTOME_METABOLISM_OF_CARBOHYDRATES |
| 46 | 6 | 0.126519 | 1 | REACTOME_METABOLISM_OF_MRNA |
| 23 | 2 | 0.365386 | 1 | REACTOME_METAL_ION_SLC_TRANSPORTERS |
| 20 | 3 | 0.291247 | 1 | REACTOME_MITOCHONDRIAL_TRNA_AMINOACYLATION |
| 32 | 3 | 0.413796 | 1 | REACTOME_MRNA_PROCESSING |
| 103 | 6 | 0.744723 | 1 | REACTOME_MRNA_SPLICING |
| 40 | 4 | 0.374436 | 1 | REACTOME_MRNA_SPLICING_MINOR_PATHWAY |
| 50 | 5 | 0.330607 | 1 | REACTOME_MUSCLE_CONTRACTION |
| 28 | 3 | 0.608194 | 1 | REACTOME_MYOGENESSIS |
| 67 | 8 | 0.286397 | 1 | REACTOME_NCAM_SIGNALING_FOR_NEURITE_OUT_GROWTH |
| 42 | 5 | 0.391496 | 1 | REACTOME_NCAM1_INTERACTIONS |
| 21 | 2 | 0.607964 | 1 | REACTOME_NEF_MEDIATES_DOWN_MODULATION_OF_CELL_SURFACE_RECEPTORS_BY_RECRUITING_THEM_TO_CLATHRIN_ADAPTERS |
| 29 | 5 | 0.0850191 | 1 | REACTOME_NEP_NS2_INTERACTS_WITH_THE_CELLULAR_EXPORT_MACHINERY |
| 83 | 9 | 0.540775 | 1 | REACTOME_NEURORANSMITTER_RECEPTOR_BINDING_AND_DOWNSTREAM_TRANSMISSION_IN_THE_POSTSYNAPTIC_CELL |
| 47 | 2 | 0.98005 | 1 | REACTOME_NRAGE_SIGNALS_DEATH_THROUGH_JNK |
| 30 | 5 | 0.0892991 | 1 | REACTOME_NUCLEAR_IMPORT_OF_REV_PROTEIN |
| 49 | 8 | 0.138009 | 1 | REACTOME_NUCLEAR_RECEPTOR_TRANSCRIPTION_PATHWAY |
| 49 | 5 | 0.265107 | 1 | REACTOME_NUCLEOTIDE_EXCISION_REPAIR |
| 82 | 12 | 0.096359 | 1 | REACTOME_OPIOID_SIGNALLING |
| 63 | 7 | 0.102489 | 1 | REACTOME_ORC1_REMOVAL_FROM_CHROMATIN |
| 43 | 5 | 0.126989 | 1 | REACTOME_P53_INDEPENDENT_DNA_DAMAGE_RESPONSE |
| 82 | 4 | 0.97082 | 1 | REACTOME_P75_NTR_RECEPTOR_MEDIATED_SIGNALLING |
| 84 | 5 | 0.458875 | 1 | REACTOME_PEPTIDE_CHAIN_ELONGATION |
| 169 | 11 | 0.367106 | 1 | REACTOME_PEPTIDE_LIGAND_BINDING_RECEPTORS |
| 67 | 5 | 0.361506 | 1 | REACTOME_PHASE_1_FUNCTIONALIZATION_OF_COMPOUNDS |
| 52 | 2 | 0.794942 | 1 | REACTOME_PHASE_II_CONJUGATION |
| 23 | 2 | 0.395636 | 1 | REACTOME_PHOSPHOLIPASE_CMEDIATED_CASCADE |
| 21 | 2 | 0.383426 | 1 | REACTOME_PHOSPHORYLATION_OF_CD3_AND_TCR_ZETA_CHAINS |
| 34 | 3 | 0.515535 | 1 | REACTOME_PI3K_AKT_SIGNALLING |
| 38 | 2 | 0.735113 | 1 | REACTOME_PI3K_CASCADE |
| 165 | 13 | 0.659093 | 1 | REACTOME_PLATELET_ACTIVATION |
| 59 | 2 | 0.946691 | 1 | REACTOME_PLATELET_ACTIVATION_TRIGGERS |
| 26 | 3 | 0.340227 | 1 | REACTOME_PLATELET_AGGREGATION_PLUG_FORMATION |
| 85 | 8 | 0.403056 | 1 | REACTOME_PLATELET_DEGRANULATION |
| 37 | 7 | 0.167918 | 1 | REACTOME_PLC_BETA_MEDIATED_EVENTS |
| 34 | 6 | 0.224088 | 1 | REACTOME_PLC_GAMMA1_SIGNALLING |
| 32 | 2 | 0.902131 | 1 | REACTOME_POST_NMDA_RECEPTOR_ACTIVATION_EVENTS |
| 40 | 3 | 0.351476 | 1 | REACTOME_POST_TRANSLATIONAL_PROTEIN_MODIFICATION |
| 134 | 11 | 0.359166 | 1 | REACTOME_PROCESSING_OF_CAPPED_INTRON_CONTAINING_PRE_MRNA |
| 29 | 4 | 0.127219 | 1 | REACTOME_PURINE_METABOLISM |
| 22 | 2 | 0.580484 | 1 | REACTOME_PYRIMIDINE_METABOLISM |
| 114 | 7 | 0.477575 | 1 | REACTOME_REGULATION_OF_BETA_CELL_DEVELOPMENT |
| 101 | 7 | 0.327817 | 1 | REACTOME_REGULATION_OF_GENE_EXPRESSION_IN_BETA_CELLS |
| 198 | 17 | 0.311647 | 1 | REACTOME_REGULATION_OF_INSULIN_SECRETION |
| 22 | 5 | 0.199048 | 1 | REACTOME_REGULATION_OF_INSULIN_SECRETION_BY_ACETYLCHOLINE |
| 20 | 4 | 0.288797 | 1 | REACTOME_REGULATION_OF_INSULIN_SECRETION_BY_FREE_FATTY_ACIDS |
| 61 | 7 | 0.359756 | 1 | REACTOME_REGULATION_OF_INSULIN_SECRETION_BY_GLUCAGON_LIKE_PEPTIDE_1 |
| 59 | 4 | 0.679093 | 1 | REACTOME_REGULATION_OF_LIPID_METABOLISM_BY_PEROXISOME_PROLIFERATOR_ACTIVATED_RECEPTOR_ALPHA |
| 47 | 5 | 0.178148 | 1 | REACTOME_REGULATION_OF_ORNITHINE_DECARBOXYLASE |
| 114 | 6 | 0.97449 | 1 | REACTOME_RHO_GTPASE_CYCLE |
| 26 | 3 | 0.280167 | 1 | REACTOME_RNA_POL_II_CTD_PHOSPHORYLATION_AND_INTERACTION_WITH_CE |
| 33 | 3 | 0.234108 | 1 | REACTOME_RNA_POLYMERASE_I_CHAIN_ELONGATION |
| 20 | 3 | 0.138239 | 1 | REACTOME_RNA_POLYMERASE_I_PROMOTER_ESCAPE |
| 55 | 2 | 0.232268 | 1 | REACTOME_RNA_POLYMERASE_I_PROMOTER_OPENING |
| 24 | 4 | 0.119969 | 1 | REACTOME_RNA_POLYMERASE_I_TRANSCRIPTION_INITIATION |
| 21 | 2 | 0.409816 | 1 | REACTOME_RNA_POLYMERASE_I_TRANSCRIPTION_TERMINATION |
| 91 | 6 | 0.614134 | 1 | REACTOME_RNA_POLYMERASE_II_TRANSCRIPTION |
| 34 | 4 | 0.171048 | 1 | REACTOME_RNA_POLYMERASE_III_TRANSCRIPTION |
| 29 | 4 | 0.095429 | 1 | REACTOME_RNA_POLYMERASE_III_TRANSCRIPTION_INITIATION |
| 20 | 2 | 0.326537 | 1 | REACTOME_RNA_POLYMERASE_III_TRANSCRIPTION_INITIATION_FROM_TYPE_2_PROMOTER |
| 22 | 2 | 0.379816 | 1 | REACTOME_RNA_POLYMERASE_III_TRANSCRIPTION_INITIATION_FROM_TYPE_3_PROMOTER |
| 102 | 10 | 0.138469 | 1 | REACTOME_S_PHASE |
| 48 | 6 | 0.0861991 | 1 | REACTOME_SCF_BETA_TRCP_MEDIATED_DEGRADATION_OF_EMI1 |
| 52 | 5 | 0.252637 | 1 | REACTOME_SCF_SKP2_MEDIATED_DEGRADATION_OF_P27_P21 |
| 28 | 3 | 0.362976 | 1 | REACTOME_SEMA4D_IN_SEMAPHORIN_SIGNALING |
| 23 | 2 | 0.542525 | 1 | REACTOME_SEMA4D_INDUCED_CELL_MIGRATION_AND_GROWTH_CONE_COLLAPSE |
| 65 | 6 | 0.672943 | 1 | REACTOME_SEMAPHORIN_INTERACTIONS |
| 31 | 2 | 0.583914 | 1 | REACTOME_SIGNAL_AMPLIFICATION |
| 48 | 3 | 0.777012 | 1 | REACTOME_SIGNALING_BY_EGFR |
| 62 | 7 | 0.235278 | 1 | REACTOME_SIGNALING_BY_PDGF |
| 31 | 5 | 0.258047 | 1 | REACTOME_SIGNALING_BY_ROBO_RECEPTOR |
| 34 | 5 | 0.105009 | 1 | REACTOME_SIGNALLING_TO_ERKS |
| 26 | 2 | 0.524325 | 1 | REACTOME_SIGNALLING_TO_RAS |
| 169 | 13 | 0.640604 | 1 | REACTOME_SLC_MEDIATED_TRANSMEMBRANE_TRANSPORT |
| 23 | 4 | 0.133429 | 1 | REACTOME_SMOOTH_MUSCLE_CONTRACTION |
| 29 | 3 | 0.568394 | 1 | REACTOME_SPHINGOLIPID_METABOLISM |
| 21 | 3 | 0.343087 | 1 | REACTOME_STEROID_HORMONES |
| 88 | 9 | 0.123509 | 1 | REACTOME_SYNTHESIS_OF_DNA |
| 26 | 3 | 0.215878 | 1 | REACTOME_SYNTHESIS_OF_GPI_ANCHORED_PROTEINS |
| 30 | 3 | 0.396956 | 1 | REACTOME_TAT_MEDIATED_HIV1_ELONGATION_ARREST_AND_RECOVERY |
| 58 | 5 | 0.376236 | 1 | REACTOME_TCR_SIGNALING |
| 28 | 3 | 0.653163 | 1 | REACTOME_THE_ROLE_OF_NEF_IN_HIV1_REPLICATION_AND_DISEASE_PATHOGENESIS |
| 27 | 2 | 0.474215 | 1 | REACTOME_THROMBIN_SIGNALLING_THROUGH_PROTEINASE_ACTIVATED_RECEPTORS |
| 23 | 2 | 0.335277 | 1 | REACTOME_THROMBOXANE_SIGNALLING_THROUGH_TP_RECEPTOR |
| 30 | 3 | 0.351676 | 1 | REACTOME_TIGHT_JUNCTION_INTERACTIONS |
| 56 | 6 | 0.156298 | 1 | REACTOME_TOLL_LIKE_RECEPTOR_3_CASCADE |
| 23 | 2 | 0.421636 | 1 | REACTOME_TOLL_LIKE_RECEPTOR_9_CASCADE |
| 83 | 8 | 0.174618 | 1 | REACTOME_TOLL_RECEPTOR_CASCADES |
| 50 | 6 | 0.121919 | 1 | REACTOME_TRAF6_MEDIATED_INDUCTION_OF_THE_ANTIVIRAL_CYTOKINE_IFN_ALPHA_BETA_CASCADE |
| 29 | 4 | 0.304057 | 1 | REACTOME_TRAFFICKING_OF_AMPA_RECEPTORS |
| 192 | 13 | 0.215808 | 1 | REACTOME_TRANSCRIPTION |
| 44 | 4 | 0.386946 | 1 | REACTOME_TRANSCRIPTION_COUPLED_NER |
| 59 | 6 | 0.235058 | 1 | REACTOME_TRANSCRIPTION_OF_THE_HIV_GENOME |
| 119 | 10 | 0.100769 | 1 | REACTOME_TRANSLATION |
| 128 | 15 | 0.417266 | 1 | REACTOME_TRANSMISSION_ACROSS_CHEMICAL_SYNAPSES |
| 51 | 5 | 0.296677 | 1 | REACTOME_TRANSPORT_OF_MATURE_MRNA_DERIVED_FROM_AN_INTRON_CONTAINING_TRANSCRIPT |
| 29 | 5 | 0.0838292 | 1 | REACTOME_TRANSPORT_OF_RIBONUCLEOPROTEINS_INTO_THE_HOST_NUCLEUS |
| 32 | 5 | 0.096889 | 1 | REACTOME_TRANSPORT_OF_THE_SLBP_INDEPENDENT_MATURE_MRNA |
| 47 | 5 | 0.179168 | 1 | REACTOME_VIF_MEDIATED_DEGRADATION_OF_APOBEC3G |
| 84 | 5 | 0.467805 | 1 | REACTOME_VIRAL_MRNA_TRANSLATION |
| 31 | 5 | 0.150928 | 1 | REACTOME_VPR_MEDIATED_NUCLEAR_IMPORT_OF_PICS |
| 24 | 3 | 0.362716 | 1 | SA_B_CELL_RECEPTOR_COMPLEXES |
| 46 | 6 | 0.323617 | 1 | SIG_BCR_SIGNALING_PATHWAY |
| 33 | 4 | 0.227918 | 1 | SIG_CD40PATHWAYMAP |
| 44 | 5 | 0.352336 | 1 | SIG_CHEMOTAXIS |
| 27 | 3 | 0.410406 | 1 | SIG_IL4RECEPTOR_IN_B_LYPHOCYTES |
| 49 | 3 | 0.717413 | 1 | SIG_INSULIN_RECEPTOR_PATHWAY_IN_CARDIAC_MYOCYTES |
| 33 | 4 | 0.467995 | 1 | SIG_PIP3_SIGNALING_IN_B_LYMPHOCYTES |
| 63 | 7 | 0.384986 | 1 | SIG_PIP3_SIGNALING_IN_CARDIAC_MYOCTES |
| 35 | 4 | 0.237988 | 1 | SIG_REGULATION_OF_THE_ACTIN_CYTOSKELETON_BY_RHO_GTPASES |
| 34 | 4 | 0.360776 | 1 | ST_ADRENERGIC |
| 39 | 4 | 0.551564 | 1 | ST_B_CELL_ANTIGEN_RECEPTOR |
| 42 | 3 | 0.676533 | 1 | ST_DIFFERENTIATION_PATHWAY_IN_PC12_CELLS |
| 29 | 5 | 0.119769 | 1 | ST_ERK1_ERK2_MAPK_PATHWAY |
| 59 | 4 | 0.632074 | 1 | ST_FAS_SIGNALING_PATHWAY |
| 34 | 5 | 0.380256 | 1 | ST_G_ALPHA_I_PATHWAY |
| 22 | 2 | 0.530115 | 1 | ST_GA12_PATHWAY |
| 35 | 2 | 0.766872 | 1 | ST_GA13_PATHWAY |
| 27 | 2 | 0.711373 | 1 | ST_GAQ_PATHWAY |
| 25 | 2 | 0.752172 | 1 | ST_GRANULE_CELL_SURVIVAL_PATHWAY |
| 78 | 10 | 0.197908 | 1 | ST_INTEGRIN_SIGNALING_PATHWAY |
| 38 | 4 | 0.408996 | 1 | ST_JNK_MAPK_PATHWAY |
| 35 | 2 | 0.740483 | 1 | ST_P38_MAPK_PATHWAY |
| 44 | 6 | 0.146599 | 1 | ST_T_CELL_SIGNAL_TRANSDUCTION |
| 28 | 2 | 0.560674 | 1 | ST_TUMOR_NECROSIS_FACTOR_PATHWAY |
| 31 | 3 | 0.416076 | 1 | ST_WNT_BETA_CATENIN_PATHWAY |
| 89 | 5 | 0.760002 | 1 | WNT_SIGNALING |
