# Supplementary material for: Whole-Genome Pathway Analysis on 132,497 Individuals Identifies Novel Gene-Sets Associated with Body Mass Index
Source: PLoS One. 2014 Jan 31;9(1):e78546. doi: 10.1371/journal.pone.0078546 (PMC3908858; doi:10.1371/journal.pone.0078546)
Supplement: Table S4 — INRICH Results for Discovery Set cutoff top 0.5%. (DOC) [file pone.0078546.s013.doc]

Table S4. Discovery INRICH results

INRICH Results for Discovery Set cutoff top 0.5%

| Target_Size | Int_No | Empirical_P | Corrected_P | Pathway |
| --- | --- | --- | --- | --- |
| 133 | 13 | 0.00426996 | 0.393214 | KEGG_UBIQUITIN_MEDIATED_PROTEOLYSIS |
| 29 | 5 | 0.00715993 | 0.556886 | REACTOME_REGULATION_OF_GLUCOKINASE_BY_GLUCOKINASE_REGULATORY_PROTEIN |
| 29 | 5 | 0.00877991 | 0.606786 | REACTOME_NEP_NS2_INTERACTS_WITH_THE_CELLULAR_EXPORT_MACHINERY |
| 29 | 5 | 0.00881991 | 0.610778 | REACTOME_TRANSPORT_OF_RIBONUCLEOPROTEINS_INTO_THE_HOST_NUCLEUS |
| 30 | 5 | 0.00923991 | 0.630739 | REACTOME_NUCLEAR_IMPORT_OF_REV_PROTEIN |
| 32 | 5 | 0.0104799 | 0.666667 | REACTOME_TRANSPORT_OF_THE_SLBP_INDEPENDENT_MATURE_MRNA |
| 31 | 5 | 0.0117799 | 0.718563 | REACTOME_REV_MEDIATED_NUCLEAR_EXPORT_OF_HIV1_RNA |
| 31 | 5 | 0.0123299 | 0.734531 | REACTOME_VPR_MEDIATED_NUCLEAR_IMPORT_OF_PICS |
| 62 | 7 | 0.0180098 | 0.832335 | REACTOME_STEROID_METABOLISM |
| 36 | 5 | 0.0194698 | 0.852295 | KEGG_SPHINGOLIPID_METABOLISM |
| 38 | 5 | 0.0195298 | 0.852295 | REACTOME_GLUCOSE_TRANSPORT |
| 116 | 7 | 0.0206398 | 0.870259 | REACTOME_RNA_POLYMERASE_I_III_AND_MITOCHONDRIAL_TRANSCRIPTION |
| 24 | 4 | 0.0260697 | 0.924152 | BIOCARTA_NTHI_PATHWAY |
| 33 | 4 | 0.0292197 | 0.946108 | BIOCARTA_MPR_PATHWAY |
| 120 | 9 | 0.0293697 | 0.946108 | KEGG_LYSOSOME |
| 49 | 5 | 0.0367796 | 0.97006 | REACTOME_SNRNP_ASSEMBLY |
| 51 | 5 | 0.0411296 | 0.982036 | REACTOME_TRANSPORT_OF_MATURE_MRNA_DERIVED_FROM_AN_INTRON_CONTAINING_TRANSCRIPT |
| 90 | 8 | 0.0424996 | 0.982036 | REACTOME_LATE_PHASE_OF_HIV_LIFE_CYCLE |
| 99 | 9 | 0.0431296 | 0.984032 | REACTOME_TRKA_SIGNALLING_FROM_THE_PLASMA_MEMBRANE |
| 48 | 4 | 0.0518995 | 0.994012 | REACTOME_FORMATION_OF_THE_TERNARY_COMPLEX_AND_SUBSEQUENTLY_THE_43S_COMPLEX |
| 90 | 9 | 0.0520495 | 0.994012 | KEGG_DILATED_CARDIOMYOPATHY |
| 49 | 6 | 0.0541695 | 0.996008 | REACTOME_NUCLEAR_RECEPTOR_TRANSCRIPTION_PATHWAY |
| 137 | 9 | 0.0546095 | 0.996008 | REACTOME_INFLUENZA_LIFE_CYCLE |
| 35 | 4 | 0.0619494 | 0.998004 | KEGG_PRION_DISEASES |
| 78 | 4 | 0.0663493 | 0.998004 | REACTOME_RNA_POLYMERASE_I_PROMOTER_CLEARANCE |
| 25 | 3 | 0.0730693 | 0.998004 | REACTOME_GS_ALPHA_MEDIATED_EVENTS_IN_GLUCAGON_SIGNALLING |
| 103 | 8 | 0.0774292 | 0.998004 | REACTOME_HIV_LIFE_CYCLE |
| 73 | 8 | 0.0812492 | 0.998004 | KEGG_ADHERENS_JUNCTION |
| 55 | 4 | 0.0857891 | 0.998004 | REACTOME_TRANSLATION_INITIATION_COMPLEX_FORMATION |
| 95 | 7 | 0.0905591 | 1 | REACTOME_METABOLISM_OF_RNA |
| 54 | 5 | 0.0928991 | 1 | BIOCARTA_PPARA_PATHWAY |
| 46 | 4 | 0.0938991 | 1 | REACTOME_STABILIZATION_OF_P53 |
| 37 | 4 | 0.097369 | 1 | BIOCARTA_MET_PATHWAY |
| 108 | 7 | 0.105439 | 1 | REACTOME_CELL_CYCLE_CHECKPOINTS |
| 86 | 6 | 0.118249 | 1 | BIOCARTA_MAPK_PATHWAY |
| 24 | 3 | 0.118939 | 1 | REACTOME_RNA_POLYMERASE_I_TRANSCRIPTION_INITIATION |
| 21 | 3 | 0.124539 | 1 | REACTOME_STEROID_HORMONES |
| 32 | 3 | 0.124559 | 1 | REACTOME_GLUCAGON_SIGNALING_IN_METABOLIC_REGULATION |
| 57 | 4 | 0.125689 | 1 | REACTOME_AUTODEGRADATION_OF_CDH1_BY_CDH1_APC |
| 32 | 3 | 0.127429 | 1 | BIOCARTA_RHO_PATHWAY |
| 82 | 6 | 0.127669 | 1 | REACTOME_GLUCOSE_AND_OTHER_SUGAR_SLC_TRANSPORTERS |
| 38 | 4 | 0.128149 | 1 | REACTOME_TRNA_AMINOACYLATION |
| 21 | 2 | 0.129939 | 1 | REACTOME_CHOLESTEROL_BIOSYNTHESIS |
| 72 | 7 | 0.135009 | 1 | KEGG_PHOSPHATIDYLINOSITOL_SIGNALING_SYSTEM |
| 68 | 5 | 0.138449 | 1 | KEGG_PPAR_SIGNALING_PATHWAY |
| 20 | 2 | 0.140299 | 1 | BIOCARTA_ACTINY_PATHWAY |
| 51 | 5 | 0.140689 | 1 | KEGG_INOSITOL_PHOSPHATE_METABOLISM |
| 167 | 11 | 0.144509 | 1 | KEGG_ENDOCYTOSIS |
| 41 | 4 | 0.146679 | 1 | KEGG_AMINOACYL_TRNA_BIOSYNTHESIS |
| 23 | 2 | 0.150198 | 1 | REACTOME_METAL_ION_SLC_TRANSPORTERS |
| 90 | 6 | 0.150558 | 1 | REACTOME_MITOTIC_PROMETAPHASE |
| 29 | 3 | 0.157288 | 1 | KEGG_THYROID_CANCER |
| 83 | 7 | 0.159928 | 1 | REACTOME_CELL_JUNCTION_ORGANIZATION |
| 56 | 4 | 0.160358 | 1 | KEGG_ACUTE_MYELOID_LEUKEMIA |
| 42 | 4 | 0.160858 | 1 | REACTOME_NCAM1_INTERACTIONS |
| 38 | 3 | 0.161318 | 1 | KEGG_TRYPTOPHAN_METABOLISM |
| 122 | 7 | 0.162288 | 1 | KEGG_CELL_CYCLE |
| 117 | 6 | 0.162608 | 1 | REACTOME_METABOLISM_OF_CARBOHYDRATES |
| 62 | 4 | 0.168638 | 1 | REACTOME_CDC20_PHOSPHO_APC_MEDIATED_DEGRADATION_OF_CYCLIN_A |
| 34 | 4 | 0.170538 | 1 | REACTOME_PLC_GAMMA1_SIGNALLING |
| 20 | 2 | 0.171588 | 1 | REACTOME_RNA_POLYMERASE_I_PROMOTER_ESCAPE |
| 34 | 3 | 0.176338 | 1 | REACTOME_SIGNALLING_TO_ERKS |
| 23 | 2 | 0.180218 | 1 | REACTOME_TOLL_LIKE_RECEPTOR_9_CASCADE |
| 134 | 8 | 0.183718 | 1 | REACTOME_PROCESSING_OF_CAPPED_INTRON_CONTAINING_PRE_MRNA |
| 25 | 2 | 0.189568 | 1 | KEGG_GLYCOSYLPHOSPHATIDYLINOSITOL_GPI_ANCHOR_BIOSYNTHESIS |
| 29 | 3 | 0.190798 | 1 | REACTOME_SPHINGOLIPID_METABOLISM |
| 26 | 2 | 0.191208 | 1 | REACTOME_SYNTHESIS_OF_GPI_ANCHORED_PROTEINS |
| 55 | 4 | 0.191968 | 1 | KEGG_BASAL_CELL_CARCINOMA |
| 22 | 2 | 0.193958 | 1 | BIOCARTA_INTRINSIC_PATHWAY |
| 28 | 2 | 0.195398 | 1 | REACTOME_GAP_JUNCTION_TRAFFICKING |
| 23 | 2 | 0.197598 | 1 | BIOCARTA_G2_PATHWAY |
| 103 | 6 | 0.197678 | 1 | REACTOME_INNATE_IMMUNITY_SIGNALING |
| 61 | 5 | 0.199098 | 1 | REACTOME_REGULATION_OF_INSULIN_SECRETION_BY_GLUCAGON_LIKE_PEPTIDE_1 |
| 20 | 2 | 0.211378 | 1 | REACTOME_DUAL_INCISION_REACTION_IN_GG_NER |
| 46 | 4 | 0.213088 | 1 | KEGG_TYPE_II_DIABETES_MELLITUS |
| 84 | 5 | 0.215638 | 1 | KEGG_PROGESTERONE_MEDIATED_OOCYTE_MATURATION |
| 192 | 8 | 0.216978 | 1 | REACTOME_TRANSCRIPTION |
| 33 | 2 | 0.219128 | 1 | REACTOME_RNA_POLYMERASE_I_CHAIN_ELONGATION |
| 44 | 3 | 0.221138 | 1 | KEGG_VASOPRESSIN_REGULATED_WATER_REABSORPTION |
| 120 | 7 | 0.224328 | 1 | REACTOME_HOST_INTERACTIONS_OF_HIV_FACTORS |
| 28 | 2 | 0.224898 | 1 | REACTOME_G_PROTEIN_ACTIVATION |
| 22 | 2 | 0.225248 | 1 | KEGG_BETA_ALANINE_METABOLISM |
| 175 | 12 | 0.227958 | 1 | KEGG_CALCIUM_SIGNALING_PATHWAY |
| 59 | 4 | 0.228908 | 1 | ST_FAS_SIGNALING_PATHWAY |
| 29 | 2 | 0.229438 | 1 | REACTOME_RNA_POLYMERASE_III_TRANSCRIPTION_INITIATION |
| 52 | 4 | 0.232298 | 1 | REACTOME_HORMONE_BIOSYNTHESIS |
| 70 | 4 | 0.235268 | 1 | REACTOME_REGULATION_OF_APC_ACTIVATORS_BETWEEN_G1_S_AND_EARLY_ANAPHASE |
| 74 | 7 | 0.237668 | 1 | KEGG_ARRHYTHMOGENIC_RIGHT_VENTRICULAR_CARDIOMYOPATHY_ARVC |
| 155 | 8 | 0.238128 | 1 | REACTOME_MITOTIC_M_M_G1_PHASES |
| 67 | 5 | 0.239628 | 1 | REACTOME_NCAM_SIGNALING_FOR_NEURITE_OUT_GROWTH |
| 183 | 10 | 0.244438 | 1 | REACTOME_HIV_INFECTION |
| 20 | 2 | 0.244528 | 1 | REACTOME_COMPLEMENT_CASCADE |
| 29 | 3 | 0.246688 | 1 | KEGG_O_GLYCAN_BIOSYNTHESIS |
| 53 | 3 | 0.247128 | 1 | KEGG_ARGININE_AND_PROLINE_METABOLISM |
| 21 | 2 | 0.248098 | 1 | REACTOME_DOUBLE_STRAND_BREAK_REPAIR |
| 29 | 2 | 0.252327 | 1 | BIOCARTA_INFLAM_PATHWAY |
| 173 | 8 | 0.252667 | 1 | REACTOME_G_ALPHA_I_SIGNALLING_EVENTS |
| 77 | 5 | 0.258217 | 1 | KEGG_FC_EPSILON_RI_SIGNALING_PATHWAY |
| 45 | 4 | 0.258287 | 1 | BIOCARTA_KERATINOCYTE_PATHWAY |
| 91 | 6 | 0.259467 | 1 | KEGG_FC_GAMMA_R_MEDIATED_PHAGOCYTOSIS |
| 23 | 2 | 0.267427 | 1 | REACTOME_SMOOTH_MUSCLE_CONTRACTION |
| 161 | 8 | 0.268517 | 1 | REACTOME_METABOLISM_OF_AMINO_ACIDS |
| 111 | 6 | 0.273297 | 1 | KEGG_OOCYTE_MEIOSIS |
| 59 | 5 | 0.275277 | 1 | REACTOME_CELL_CELL_ADHESION_SYSTEMS |
| 22 | 2 | 0.285987 | 1 | ST_GA12_PATHWAY |
| 79 | 4 | 0.287367 | 1 | REACTOME_G2_M_TRANSITION |
| 83 | 6 | 0.288887 | 1 | KEGG_HYPERTROPHIC_CARDIOMYOPATHY_HCM |
| 20 | 2 | 0.290877 | 1 | REACTOME_MITOCHONDRIAL_TRNA_AMINOACYLATION |
| 39 | 3 | 0.295167 | 1 | ST_B_CELL_ANTIGEN_RECEPTOR |
| 30 | 2 | 0.297297 | 1 | REACTOME_TIGHT_JUNCTION_INTERACTIONS |
| 28 | 2 | 0.301307 | 1 | ST_TUMOR_NECROSIS_FACTOR_PATHWAY |
| 25 | 2 | 0.301667 | 1 | BIOCARTA_STRESS_PATHWAY |
| 21 | 2 | 0.302057 | 1 | REACTOME_CYTOSOLIC_TRNA_AMINOACYLATION |
| 50 | 3 | 0.304697 | 1 | REACTOME_MUSCLE_CONTRACTION |
| 49 | 3 | 0.305137 | 1 | REACTOME_CYTOCHROME_P450_ARRANGED_BY_SUBSTRATE_TYPE |
| 62 | 4 | 0.306007 | 1 | REACTOME_SIGNALING_BY_PDGF |
| 58 | 3 | 0.307097 | 1 | KEGG_NOD_LIKE_RECEPTOR_SIGNALING_PATHWAY |
| 126 | 7 | 0.310067 | 1 | KEGG_NEUROTROPHIN_SIGNALING_PATHWAY |
| 40 | 2 | 0.310327 | 1 | REACTOME_POST_TRANSLATIONAL_PROTEIN_MODIFICATION |
| 70 | 4 | 0.314147 | 1 | REACTOME_METABLISM_OF_NUCLEOTIDES |
| 44 | 3 | 0.316427 | 1 | SIG_CHEMOTAXIS |
| 169 | 7 | 0.316807 | 1 | REACTOME_PEPTIDE_LIGAND_BINDING_RECEPTORS |
| 34 | 2 | 0.317877 | 1 | REACTOME_RNA_POLYMERASE_III_TRANSCRIPTION |
| 95 | 5 | 0.326207 | 1 | KEGG_PYRIMIDINE_METABOLISM |
| 22 | 2 | 0.326857 | 1 | BIOCARTA_HER2_PATHWAY |
| 155 | 9 | 0.327337 | 1 | KEGG_PURINE_METABOLISM |
| 66 | 4 | 0.328567 | 1 | KEGG_P53_SIGNALING_PATHWAY |
| 99 | 6 | 0.332287 | 1 | KEGG_GNRH_SIGNALING_PATHWAY |
| 52 | 4 | 0.332407 | 1 | KEGG_ENDOMETRIAL_CANCER |
| 30 | 2 | 0.337577 | 1 | REACTOME_INHIBITION_OF_INSULIN_SECRETION_BY_ADRENALINE_NORADRENALINE |
| 35 | 2 | 0.337587 | 1 | SIG_REGULATION_OF_THE_ACTIN_CYTOSKELETON_BY_RHO_GTPASES |
| 22 | 2 | 0.338877 | 1 | BIOCARTA_P53HYPOXIA_PATHWAY |
| 101 | 4 | 0.341787 | 1 | REACTOME_REGULATION_OF_GENE_EXPRESSION_IN_BETA_CELLS |
| 169 | 9 | 0.345897 | 1 | REACTOME_SLC_MEDIATED_TRANSMEMBRANE_TRANSPORT |
| 83 | 4 | 0.350836 | 1 | REACTOME_TOLL_RECEPTOR_CASCADES |
| 24 | 2 | 0.354446 | 1 | KEGG_DORSO_VENTRAL_AXIS_FORMATION |
| 58 | 3 | 0.354776 | 1 | REACTOME_CYCLIN_E_ASSOCIATED_EVENTS_DURING_G1_S_TRANSITION_ |
| 29 | 2 | 0.356356 | 1 | BIOCARTA_TNFR1_PATHWAY |
| 27 | 2 | 0.359606 | 1 | BIOCARTA_PYK2_PATHWAY |
| 85 | 4 | 0.360526 | 1 | KEGG_HEMATOPOIETIC_CELL_LINEAGE |
| 58 | 3 | 0.362306 | 1 | REACTOME_SIGNALING_BY_WNT |
| 26 | 2 | 0.364146 | 1 | KEGG_SELENOAMINO_ACID_METABOLISM |
| 28 | 2 | 0.365476 | 1 | KEGG_HOMOLOGOUS_RECOMBINATION |
| 105 | 4 | 0.365926 | 1 | REACTOME_GTP_HYDROLYSIS_AND_JOINING_OF_THE_60S_RIBOSOMAL_SUBUNIT |
| 70 | 4 | 0.366516 | 1 | KEGG_CARDIAC_MUSCLE_CONTRACTION |
| 25 | 2 | 0.367966 | 1 | REACTOME_CAM_PATHWAY |
| 35 | 2 | 0.369016 | 1 | BIOCARTA_TOLL_PATHWAY |
| 28 | 2 | 0.370236 | 1 | KEGG_ASTHMA |
| 72 | 4 | 0.377776 | 1 | KEGG_CHRONIC_MYELOID_LEUKEMIA |
| 33 | 2 | 0.378246 | 1 | REACTOME_GLOBAL_GENOMIC_NER |
| 62 | 4 | 0.379626 | 1 | KEGG_COLORECTAL_CANCER |
| 36 | 2 | 0.381006 | 1 | REACTOME_ACTIVATION_OF_ATR_IN_RESPONSE_TO_REPLICATION_STRESS |
| 32 | 2 | 0.382096 | 1 | BIOCARTA_IL1R_PATHWAY |
| 32 | 2 | 0.386426 | 1 | REACTOME_FORMATION_OF_FIBRIN_CLOT_CLOTTING_CASCADE |
| 27 | 2 | 0.388166 | 1 | REACTOME_METABOLISM_OF_BILE_ACIDS_AND_BILE_SALTS |
| 53 | 3 | 0.391356 | 1 | KEGG_VIBRIO_CHOLERAE_INFECTION |
| 78 | 5 | 0.395636 | 1 | ST_INTEGRIN_SIGNALING_PATHWAY |
| 33 | 2 | 0.404666 | 1 | SIG_CD40PATHWAYMAP |
| 122 | 6 | 0.404856 | 1 | REACTOME_G_ALPHA_S_SIGNALLING_EVENTS |
| 37 | 3 | 0.406186 | 1 | REACTOME_PLC_BETA_MEDIATED_EVENTS |
| 23 | 2 | 0.409116 | 1 | ST_MYOCYTE_AD_PATHWAY |
| 110 | 5 | 0.409406 | 1 | KEGG_PARKINSONS_DISEASE |
| 56 | 3 | 0.409696 | 1 | BIOCARTA_HIVNEF_PATHWAY |
| 124 | 4 | 0.412556 | 1 | KEGG_SYSTEMIC_LUPUS_ERYTHEMATOSUS |
| 84 | 3 | 0.415456 | 1 | REACTOME_PEPTIDE_CHAIN_ELONGATION |
| 113 | 7 | 0.416576 | 1 | KEGG_VASCULAR_SMOOTH_MUSCLE_CONTRACTION |
| 35 | 2 | 0.419776 | 1 | REACTOME_DOWN_STREAM_SIGNAL_TRANSDUCTION |
| 82 | 5 | 0.419786 | 1 | REACTOME_OPIOID_SIGNALLING |
| 27 | 2 | 0.420796 | 1 | ST_GAQ_PATHWAY |
| 84 | 3 | 0.421086 | 1 | REACTOME_VIRAL_MRNA_TRANSLATION |
| 26 | 2 | 0.421686 | 1 | BIOCARTA_EDG1_PATHWAY |
| 34 | 2 | 0.422136 | 1 | KEGG_CYSTEINE_AND_METHIONINE_METABOLISM |
| 86 | 3 | 0.425046 | 1 | KEGG_RIBOSOME |
| 35 | 2 | 0.429096 | 1 | ST_GA13_PATHWAY |
| 31 | 2 | 0.430826 | 1 | BIOCARTA_AT1R_PATHWAY |
| 67 | 3 | 0.435526 | 1 | REACTOME_PHASE_1_FUNCTIONALIZATION_OF_COMPOUNDS |
| 104 | 5 | 0.436136 | 1 | REACTOME_DNA_REPAIR |
| 43 | 2 | 0.442186 | 1 | KEGG_DRUG_METABOLISM_OTHER_ENZYMES |
| 119 | 4 | 0.443126 | 1 | REACTOME_TRANSLATION |
| 55 | 3 | 0.448146 | 1 | KEGG_RNA_DEGRADATION |
| 37 | 2 | 0.448546 | 1 | KEGG_GRAFT_VERSUS_HOST_DISEASE |
| 46 | 3 | 0.448546 | 1 | SIG_BCR_SIGNALING_PATHWAY |
| 35 | 2 | 0.451735 | 1 | ST_P38_MAPK_PATHWAY |
| 29 | 3 | 0.456635 | 1 | REACTOME_ADHERENS_JUNCTIONS_INTERACTIONS |
| 32 | 2 | 0.457945 | 1 | REACTOME_ACTIVATION_OF_KAINATE_RECEPTORS_UPON_GLUTAMATE_BINDING |
| 41 | 2 | 0.458295 | 1 | REACTOME_G2_M_CHECKPOINTS |
| 114 | 4 | 0.459425 | 1 | REACTOME_REGULATION_OF_BETA_CELL_DEVELOPMENT |
| 40 | 2 | 0.459605 | 1 | KEGG_PYRUVATE_METABOLISM |
| 28 | 2 | 0.459725 | 1 | REACTOME_ASSOCIATION_OF_TRIC_CCT_WITH_TARGET_PROTEINS_DURING_BIOSYNTHESIS |
| 131 | 8 | 0.465005 | 1 | KEGG_CELL_ADHESION_MOLECULES_CAMS |
| 29 | 2 | 0.473545 | 1 | REACTOME_AMINE_COMPOUND_SLC_TRANSPORTERS |
| 88 | 5 | 0.477785 | 1 | KEGG_GAP_JUNCTION |
| 35 | 2 | 0.481855 | 1 | BIOCARTA_FMLP_PATHWAY |
| 83 | 5 | 0.484205 | 1 | REACTOME_NEURORANSMITTER_RECEPTOR_BINDING_AND_DOWNSTREAM_TRANSMISSION_IN_THE_POSTSYNAPTIC_CELL |
| 44 | 2 | 0.484465 | 1 | KEGG_NUCLEOTIDE_EXCISION_REPAIR |
| 39 | 2 | 0.486795 | 1 | REACTOME_HIV1_TRANSCRIPTION_INITIATION |
| 43 | 2 | 0.486905 | 1 | REACTOME_P53_INDEPENDENT_DNA_DAMAGE_RESPONSE |
| 35 | 2 | 0.487605 | 1 | KEGG_ALLOGRAFT_REJECTION |
| 29 | 2 | 0.492695 | 1 | REACTOME_TRAFFICKING_OF_AMPA_RECEPTORS |
| 85 | 4 | 0.494595 | 1 | KEGG_TGF_BETA_SIGNALING_PATHWAY |
| 59 | 3 | 0.497755 | 1 | REACTOME_TRANSCRIPTION_OF_THE_HIV_GENOME |
| 41 | 2 | 0.505405 | 1 | REACTOME_MAP_KINASES_ACTIVATION_IN_TLR_CASCADE |
| 66 | 3 | 0.506975 | 1 | KEGG_LEISHMANIA_INFECTION |
| 32 | 2 | 0.509635 | 1 | REACTOME_FORMATION_OF_THE_EARLY_ELONGATION_COMPLEX |
| 94 | 3 | 0.510965 | 1 | REACTOME_FORMATION_OF_A_POOL_OF_FREE_40S_SUBUNITS |
| 154 | 7 | 0.511395 | 1 | KEGG_ALZHEIMERS_DISEASE |
| 66 | 3 | 0.513145 | 1 | KEGG_ADIPOCYTOKINE_SIGNALING_PATHWAY |
| 39 | 2 | 0.513815 | 1 | BIOCARTA_P38MAPK_PATHWAY |
| 43 | 2 | 0.517075 | 1 | KEGG_LYSINE_DEGRADATION |
| 38 | 2 | 0.523575 | 1 | BIOCARTA_INTEGRIN_PATHWAY |
| 44 | 2 | 0.529405 | 1 | KEGG_PROTEASOME |
| 48 | 2 | 0.533225 | 1 | REACTOME_AMINO_ACID_AND_OLIGOPEPTIDE_SLC_TRANSPORTERS |
| 34 | 2 | 0.535535 | 1 | ST_ADRENERGIC |
| 47 | 2 | 0.537905 | 1 | REACTOME_VIF_MEDIATED_DEGRADATION_OF_APOBEC3G |
| 47 | 2 | 0.541565 | 1 | REACTOME_REGULATION_OF_ORNITHINE_DECARBOXYLASE |
| 47 | 2 | 0.547015 | 1 | KEGG_STEROID_HORMONE_BIOSYNTHESIS |
| 28 | 2 | 0.547265 | 1 | REACTOME_MYOGENESSIS |
| 119 | 4 | 0.549065 | 1 | REACTOME_BIOLOGICAL_OXIDATIONS |
| 42 | 2 | 0.552794 | 1 | KEGG_BLADDER_CANCER |
| 42 | 2 | 0.553574 | 1 | ST_DIFFERENTIATION_PATHWAY_IN_PC12_CELLS |
| 48 | 2 | 0.553664 | 1 | REACTOME_SCF_BETA_TRCP_MEDIATED_DEGRADATION_OF_EMI1 |
| 49 | 2 | 0.553834 | 1 | REACTOME_CHAPERONIN_MEDIATED_PROTEIN_FOLDING |
| 73 | 3 | 0.556434 | 1 | KEGG_GLYCEROPHOSPHOLIPID_METABOLISM |
| 81 | 3 | 0.557914 | 1 | KEGG_ANTIGEN_PROCESSING_AND_PRESENTATION |
| 38 | 2 | 0.558674 | 1 | ST_JNK_MAPK_PATHWAY |
| 100 | 3 | 0.561904 | 1 | KEGG_TOLL_LIKE_RECEPTOR_SIGNALING_PATHWAY |
| 68 | 2 | 0.573264 | 1 | REACTOME_IMMUNOREGULATORY_INTERACTIONS_BETWEEN_A_LYMPHOID_AND_A_NON_LYMPHOID_CELL |
| 32 | 2 | 0.575684 | 1 | REACTOME_POST_NMDA_RECEPTOR_ACTIVATION_EVENTS |
| 53 | 3 | 0.576774 | 1 | KEGG_NON_SMALL_CELL_LUNG_CANCER |
| 128 | 7 | 0.582584 | 1 | REACTOME_TRANSMISSION_ACROSS_CHEMICAL_SYNAPSES |
| 50 | 2 | 0.584774 | 1 | REACTOME_TRAF6_MEDIATED_INDUCTION_OF_THE_ANTIVIRAL_CYTOKINE_IFN_ALPHA_BETA_CASCADE |
| 33 | 2 | 0.590704 | 1 | SIG_PIP3_SIGNALING_IN_B_LYMPHOCYTES |
| 52 | 2 | 0.594704 | 1 | REACTOME_SCF_SKP2_MEDIATED_DEGRADATION_OF_P27_P21 |
| 49 | 2 | 0.600884 | 1 | SIG_INSULIN_RECEPTOR_PATHWAY_IN_CARDIAC_MYOCYTES |
| 46 | 2 | 0.601684 | 1 | KEGG_GLYCEROLIPID_METABOLISM |
| 41 | 2 | 0.602254 | 1 | REACTOME_HIV1_TRANSCRIPTION_ELONGATION |
| 52 | 2 | 0.605404 | 1 | REACTOME_CDT1_ASSOCIATION_WITH_THE_CDC6_ORC_ORIGIN_COMPLEX |
| 100 | 3 | 0.606204 | 1 | REACTOME_INFLUENZA_VIRAL_RNA_TRANSCRIPTION_AND_REPLICATION |
| 44 | 2 | 0.606914 | 1 | KEGG_AMINO_SUGAR_AND_NUCLEOTIDE_SUGAR_METABOLISM |
| 68 | 3 | 0.610194 | 1 | KEGG_COMPLEMENT_AND_COAGULATION_CASCADES |
| 100 | 4 | 0.611984 | 1 | KEGG_MELANOGENESIS |
| 50 | 2 | 0.612604 | 1 | KEGG_AUTOIMMUNE_THYROID_DISEASE |
| 83 | 4 | 0.614244 | 1 | KEGG_SMALL_CELL_LUNG_CANCER |
| 70 | 3 | 0.617534 | 1 | KEGG_PANCREATIC_CANCER |
| 56 | 2 | 0.620164 | 1 | REACTOME_TOLL_LIKE_RECEPTOR_3_CASCADE |
| 34 | 2 | 0.621964 | 1 | ST_G_ALPHA_I_PATHWAY |
| 44 | 2 | 0.626044 | 1 | ST_T_CELL_SIGNAL_TRANSDUCTION |
| 46 | 2 | 0.642964 | 1 | REACTOME_METABOLISM_OF_MRNA |
| 41 | 2 | 0.645324 | 1 | KEGG_TYPE_I_DIABETES_MELLITUS |
| 49 | 2 | 0.652193 | 1 | REACTOME_NUCLEOTIDE_EXCISION_REPAIR |
| 129 | 4 | 0.656953 | 1 | REACTOME_INSULIN_SYNTHESIS_AND_SECRETION |
| 54 | 2 | 0.660523 | 1 | REACTOME_GOLGI_ASSOCIATED_VESICLE_BIOGENESIS |
| 56 | 2 | 0.660813 | 1 | KEGG_HEDGEHOG_SIGNALING_PATHWAY |
| 183 | 6 | 0.661673 | 1 | KEGG_CHEMOKINE_SIGNALING_PATHWAY |
| 198 | 7 | 0.661973 | 1 | REACTOME_REGULATION_OF_INSULIN_SECRETION |
| 36 | 2 | 0.671213 | 1 | REACTOME_ACTIVATION_OF_NMDA_RECEPTOR_UPON_GLUTAMATE_BINDING_AND_POSTSYNAPTIC_EVENTS |
| 68 | 3 | 0.678693 | 1 | KEGG_VIRAL_MYOCARDITIS |
| 61 | 2 | 0.679013 | 1 | REACTOME_M_G1_TRANSITION |
| 169 | 6 | 0.683953 | 1 | KEGG_HUNTINGTONS_DISEASE |
| 118 | 4 | 0.685953 | 1 | KEGG_SPLICEOSOME |
| 63 | 2 | 0.688763 | 1 | REACTOME_ORC1_REMOVAL_FROM_CHROMATIN |
| 67 | 2 | 0.689903 | 1 | REACTOME_CENTROSOME_MATURATION |
| 148 | 5 | 0.694183 | 1 | REACTOME_FORMATION_AND_MATURATION_OF_MRNA_TRANSCRIPT |
| 128 | 6 | 0.697263 | 1 | KEGG_TIGHT_JUNCTION |
| 74 | 2 | 0.706783 | 1 | REACTOME_IRS_RELATED_EVENTS |
| 61 | 2 | 0.713013 | 1 | REACTOME_CLATHRIN_DERIVED_VESICLE_BUDDING |
| 84 | 4 | 0.726493 | 1 | KEGG_ECM_RECEPTOR_INTERACTION |
| 100 | 3 | 0.727883 | 1 | REACTOME_G1_S_TRANSITION |
| 80 | 3 | 0.732763 | 1 | REACTOME_INTEGRIN_CELL_SURFACE_INTERACTIONS |
| 113 | 3 | 0.735983 | 1 | KEGG_OXIDATIVE_PHOSPHORYLATION |
| 59 | 2 | 0.737513 | 1 | REACTOME_REGULATION_OF_LIPID_METABOLISM_BY_PEROXISOME_PROLIFERATOR_ACTIVATED_RECEPTOR_ALPHA |
| 88 | 3 | 0.743463 | 1 | KEGG_PROSTATE_CANCER |
| 91 | 3 | 0.745743 | 1 | REACTOME_RNA_POLYMERASE_II_TRANSCRIPTION |
| 102 | 3 | 0.748923 | 1 | REACTOME_S_PHASE |
| 147 | 4 | 0.751512 | 1 | REACTOME_GLUCOSE_REGULATION_OF_INSULIN_SECRETION |
| 157 | 7 | 0.762012 | 1 | REACTOME_AXON_GUIDANCE |
| 130 | 4 | 0.762232 | 1 | REACTOME_ELONGATION_AND_PROCESSING_OF_CAPPED_TRANSCRIPTS |
| 53 | 2 | 0.765892 | 1 | KEGG_AMYOTROPHIC_LATERAL_SCLEROSIS_ALS |
| 128 | 6 | 0.770582 | 1 | KEGG_AXON_GUIDANCE |
| 36 | 2 | 0.770612 | 1 | BIOCARTA_AGR_PATHWAY |
| 195 | 8 | 0.775742 | 1 | KEGG_FOCAL_ADHESION |
| 75 | 2 | 0.786132 | 1 | REACTOME_DNA_REPLICATION_PRE_INITIATION |
| 70 | 2 | 0.787432 | 1 | KEGG_RENAL_CELL_CARCINOMA |
| 63 | 2 | 0.788902 | 1 | KEGG_GLIOMA |
| 69 | 3 | 0.801852 | 1 | KEGG_LONG_TERM_POTENTIATION |
| 184 | 6 | 0.805142 | 1 | REACTOME_FORMATION_OF_PLATELET_PLUG |
| 103 | 3 | 0.810962 | 1 | REACTOME_MRNA_SPLICING |
| 94 | 3 | 0.820652 | 1 | REACTOME_INORGANIC_CATION_ANION_SLC_TRANSPORTERS |
| 78 | 2 | 0.822752 | 1 | REACTOME_MEMBRANE_TRAFFICKING |
| 63 | 2 | 0.827532 | 1 | SIG_PIP3_SIGNALING_IN_CARDIAC_MYOCTES |
| 108 | 3 | 0.839682 | 1 | KEGG_T_CELL_RECEPTOR_SIGNALING_PATHWAY |
| 165 | 5 | 0.843152 | 1 | REACTOME_PLATELET_ACTIVATION |
| 85 | 2 | 0.843782 | 1 | REACTOME_PLATELET_DEGRANULATION |
| 74 | 2 | 0.845692 | 1 | KEGG_B_CELL_RECEPTOR_SIGNALING_PATHWAY |
| 88 | 2 | 0.857221 | 1 | REACTOME_SYNTHESIS_OF_DNA |
| 71 | 2 | 0.862761 | 1 | KEGG_MELANOMA |
| 112 | 3 | 0.898301 | 1 | KEGG_LEUKOCYTE_TRANSENDOTHELIAL_MIGRATION |
| 128 | 3 | 0.901261 | 1 | REACTOME_APOPTOSIS |
| 153 | 4 | 0.912291 | 1 | REACTOME_G_ALPHA_Q_SIGNALLING_EVENTS |
| 68 | 2 | 0.922981 | 1 | KEGG_LONG_TERM_DEPRESSION |
| 131 | 2 | 0.923931 | 1 | KEGG_NATURAL_KILLER_CELL_MEDIATED_CYTOTOXICITY |
| 114 | 3 | 0.944511 | 1 | REACTOME_RHO_GTPASE_CYCLE |
| 85 | 2 | 0.95829 | 1 | KEGG_ERBB_SIGNALING_PATHWAY |
| 149 | 3 | 0.9672 | 1 | KEGG_WNT_SIGNALING_PATHWAY |
