# Supplementary material for: Whole-Genome Pathway Analysis on 132,497 Individuals Identifies Novel Gene-Sets Associated with Body Mass Index
Source: PLoS One. 2014 Jan 31;9(1):e78546. doi: 10.1371/journal.pone.0078546 (PMC3908858; doi:10.1371/journal.pone.0078546)
Supplement: Table S5 — INRICH Results for Replication Set cutoff top 10%. (DOC) [file pone.0078546.s014.doc]

Table S5. Replication INRICH results

INRICH Results for Replication Set cutoff top 10%

| Target_Size | Int_No | Empirical_P | Corrected_P | Pathway |
| --- | --- | --- | --- | --- |
| 77 | 45 | 0.000579994 | * 0.025195 | KEGG_FC_EPSILON_RI_SIGNALING_PATHWAY |
| 100 | 46 | 0.00241998 | * 0.049982 | KEGG_TOLL_LIKE_RECEPTOR_SIGNALING_PATHWAY |
| 126 | 67 | 0.00639994 | 0.218356 | KEGG_NEUROTROPHIN_SIGNALING_PATHWAY |
| 38 | 26 | 0.00818992 | 0.276145 | ST_JNK_MAPK_PATHWAY |
| 79 | 42 | 0.00914991 | 0.295141 | REACTOME_G2_M_TRANSITION |
| 108 | 51 | 0.0107999 | 0.342931 | REACTOME_CELL_CYCLE_CHECKPOINTS |
| 44 | 23 | 0.0112599 | 0.361928 | KEGG_PROTEASOME |
| 26 | 16 | 0.0117199 | 0.372525 | BIOCARTA_RACCYCD_PATHWAY |
| 46 | 23 | 0.0159398 | 0.435313 | REACTOME_STABILIZATION_OF_P53 |
| 99 | 55 | 0.0197898 | 0.489702 | KEGG_GNRH_SIGNALING_PATHWAY |
| 103 | 49 | 0.0213498 | 0.528694 | REACTOME_INNATE_IMMUNITY_SIGNALING |
| 74 | 36 | 0.0247898 | 0.584683 | KEGG_VEGF_SIGNALING_PATHWAY |
| 47 | 22 | 0.0300697 | 0.640872 | REACTOME_VIF_MEDIATED_DEGRADATION_OF_APOBEC3G |
| 47 | 22 | 0.0309397 | 0.64927 | REACTOME_REGULATION_OF_ORNITHINE_DECARBOXYLASE |
| 21 | 13 | 0.0359796 | 0.694661 | REACTOME_ERK_MAPK_TARGETS |
| 43 | 20 | 0.0370096 | 0.70246 | REACTOME_P53_INDEPENDENT_DNA_DAMAGE_RESPONSE |
| 58 | 27 | 0.0374796 | 0.711458 | REACTOME_CYCLIN_E_ASSOCIATED_EVENTS_DURING_G1_S_TRANSITION_ |
| 44 | 25 | 0.0387196 | 0.723855 | KEGG_AMINO_SUGAR_AND_NUCLEOTIDE_SUGAR_METABOLISM |
| 155 | 70 | 0.0390996 | 0.724255 | REACTOME_MITOTIC_M_M_G1_PHASES |
| 83 | 40 | 0.0398096 | 0.728854 | REACTOME_TOLL_RECEPTOR_CASCADES |
| 39 | 24 | 0.0406596 | 0.734653 | ST_B_CELL_ANTIGEN_RECEPTOR |
| 108 | 54 | 0.0417796 | 0.74945 | KEGG_T_CELL_RECEPTOR_SIGNALING_PATHWAY |
| 100 | 44 | 0.0432996 | 0.759048 | REACTOME_G1_S_TRANSITION |
| 30 | 18 | 0.0509295 | 0.817437 | REACTOME_MAPK_TARGETS_NUCLEAR_EVENTS_MEDIATED_BY_MAP_KINASES |
| 41 | 23 | 0.0524595 | 0.823835 | REACTOME_MAP_KINASES_ACTIVATION_IN_TLR_CASCADE |
| 63 | 28 | 0.0527795 | 0.824235 | REACTOME_ORC1_REMOVAL_FROM_CHROMATIN |
| 67 | 34 | 0.0571794 | 0.834233 | REACTOME_CENTROSOME_MATURATION |
| 53 | 31 | 0.0593294 | 0.846031 | KEGG_NON_SMALL_CELL_LUNG_CANCER |
| 58 | 27 | 0.0622994 | 0.859428 | REACTOME_SIGNALING_BY_WNT |
| 154 | 73 | 0.0626994 | 0.861628 | KEGG_ALZHEIMERS_DISEASE |
| 52 | 23 | 0.0631094 | 0.870026 | REACTOME_CDT1_ASSOCIATION_WITH_THE_CDC6_ORC_ORIGIN_COMPLEX |
| 48 | 22 | 0.0635194 | 0.870426 | REACTOME_SCF_BETA_TRCP_MEDIATED_DEGRADATION_OF_EMI1 |
| 50 | 26 | 0.0640994 | 0.872226 | REACTOME_TRAF6_MEDIATED_INDUCTION_OF_THE_ANTIVIRAL_CYTOKINE_IFN_ALPHA_BETA_CASCADE |
| 24 | 14 | 0.0694393 | 0.888022 | REACTOME_NUCLEAR_EVENTS_KINASE_AND_TRANSCRIPTION_FACTOR_ACTIVATION |
| 54 | 27 | 0.0746393 | 0.910618 | BIOCARTA_PPARA_PATHWAY |
| 62 | 27 | 0.0760292 | 0.911818 | REACTOME_CDC20_PHOSPHO_APC_MEDIATED_DEGRADATION_OF_CYCLIN_A |
| 23 | 13 | 0.0798892 | 0.915817 | BIOCARTA_RAS_PATHWAY |
| 148 | 58 | 0.0804192 | 0.916417 | REACTOME_FORMATION_AND_MATURATION_OF_MRNA_TRANSCRIPT |
| 41 | 21 | 0.0847592 | 0.924415 | REACTOME_G2_M_CHECKPOINTS |
| 90 | 44 | 0.0877491 | 0.931614 | REACTOME_MITOTIC_PROMETAPHASE |
| 61 | 26 | 0.097539 | 0.94761 | REACTOME_M_G1_TRANSITION |
| 35 | 18 | 0.101869 | 0.954009 | REACTOME_DOWN_STREAM_SIGNAL_TRANSDUCTION |
| 70 | 37 | 0.109569 | 0.959608 | KEGG_PANCREATIC_CANCER |
| 56 | 27 | 0.112979 | 0.964807 | REACTOME_TOLL_LIKE_RECEPTOR_3_CASCADE |
| 88 | 44 | 0.120429 | 0.970006 | KEGG_PROSTATE_CANCER |
| 42 | 22 | 0.123869 | 0.975405 | BIOCARTA_CHREBP2_PATHWAY |
| 52 | 22 | 0.141689 | 0.982603 | REACTOME_SCF_SKP2_MEDIATED_DEGRADATION_OF_P27_P21 |
| 183 | 75 | 0.146229 | 0.984603 | REACTOME_HIV_INFECTION |
| 75 | 30 | 0.167198 | 0.991602 | REACTOME_DNA_REPLICATION_PRE_INITIATION |
| 70 | 29 | 0.169078 | 0.992002 | REACTOME_REGULATION_OF_APC_ACTIVATORS_BETWEEN_G1_S_AND_EARLY_ANAPHASE |
| 59 | 29 | 0.173098 | 0.993001 | REACTOME_LOSS_OF_NLP_FROM_MITOTIC_CENTROSOMES |
| 90 | 39 | 0.182828 | 0.994601 | REACTOME_LATE_PHASE_OF_HIV_LIFE_CYCLE |
| 63 | 32 | 0.183468 | 0.994801 | SIG_PIP3_SIGNALING_IN_CARDIAC_MYOCTES |
| 59 | 27 | 0.186888 | 0.995001 | ST_FAS_SIGNALING_PATHWAY |
| 27 | 15 | 0.204178 | 0.996401 | BIOCARTA_PYK2_PATHWAY |
| 37 | 17 | 0.211208 | 0.996801 | KEGG_SNARE_INTERACTIONS_IN_VESICULAR_TRANSPORT |
| 36 | 17 | 0.222068 | 0.997401 | REACTOME_ACTIVATION_OF_ATR_IN_RESPONSE_TO_REPLICATION_STRESS |
| 22 | 12 | 0.260237 | 0.9986 | BIOCARTA_EIF4_PATHWAY |
| 31 | 17 | 0.284877 | 0.9996 | BIOCARTA_AT1R_PATHWAY |
| 35 | 17 | 0.296147 | 0.9998 | REACTOME_GENERIC_TRANSCRIPTION_PATHWAY |
| 74 | 31 | 0.299127 | 0.9998 | REACTOME_IRS_RELATED_EVENTS |
| 40 | 16 | 0.309307 | 0.9998 | REACTOME_MRNA_SPLICING_MINOR_PATHWAY |
| 66 | 32 | 0.325837 | 1 | KEGG_EPITHELIAL_CELL_SIGNALING_IN_HELICOBACTER_PYLORI_INFECTION |
| 120 | 49 | 0.333537 | 1 | REACTOME_HOST_INTERACTIONS_OF_HIV_FACTORS |
| 78 | 34 | 0.377466 | 1 | REACTOME_MEMBRANE_TRAFFICKING |
| 39 | 19 | 0.380246 | 1 | BIOCARTA_P38MAPK_PATHWAY |
| 86 | 37 | 0.395416 | 1 | BIOCARTA_MAPK_PATHWAY |
| 84 | 36 | 0.399706 | 1 | KEGG_PROGESTERONE_MEDIATED_OOCYTE_MATURATION |
| 99 | 47 | 0.436576 | 1 | REACTOME_TRKA_SIGNALLING_FROM_THE_PLASMA_MEMBRANE |
| 58 | 22 | 0.446826 | 1 | KEGG_NOD_LIKE_RECEPTOR_SIGNALING_PATHWAY |
| 120 | 48 | 0.458705 | 1 | KEGG_LYSOSOME |
| 72 | 39 | 0.459025 | 1 | KEGG_PHOSPHATIDYLINOSITOL_SIGNALING_SYSTEM |
| 44 | 20 | 0.486565 | 1 | ST_T_CELL_SIGNAL_TRANSDUCTION |
| 26 | 12 | 0.503875 | 1 | KEGG_GALACTOSE_METABOLISM |
| 134 | 48 | 0.528785 | 1 | REACTOME_PROCESSING_OF_CAPPED_INTRON_CONTAINING_PRE_MRNA |
| 33 | 13 | 0.569154 | 1 | BIOCARTA_MPR_PATHWAY |
| 51 | 25 | 0.639854 | 1 | KEGG_INOSITOL_PHOSPHATE_METABOLISM |
| 35 | 12 | 0.661093 | 1 | SIG_REGULATION_OF_THE_ACTIN_CYTOSKELETON_BY_RHO_GTPASES |
| 58 | 21 | 0.685033 | 1 | REACTOME_TCR_SIGNALING |
| 32 | 12 | 0.744733 | 1 | REACTOME_TRANSPORT_OF_THE_SLBP_INDEPENDENT_MATURE_MRNA |
| 29 | 11 | 0.772952 | 1 | REACTOME_REGULATION_OF_GLUCOKINASE_BY_GLUCOKINASE_REGULATORY_PROTEIN |
| 32 | 9 | 0.773692 | 1 | REACTOME_GENERATION_OF_SECOND_MESSENGER_MOLECULES |
| 31 | 11 | 0.844352 | 1 | REACTOME_REV_MEDIATED_NUCLEAR_EXPORT_OF_HIV1_RNA |
| 29 | 10 | 0.878941 | 1 | REACTOME_NEP_NS2_INTERACTS_WITH_THE_CELLULAR_EXPORT_MACHINERY |
| 30 | 9 | 0.95441 | 1 | REACTOME_NUCLEAR_IMPORT_OF_REV_PROTEIN |
