# Supplementary material for: Whole-Genome Pathway Analysis on 132,497 Individuals Identifies Novel Gene-Sets Associated with Body Mass Index
Source: PLoS One. 2014 Jan 31;9(1):e78546. doi: 10.1371/journal.pone.0078546 (PMC3908858; doi:10.1371/journal.pone.0078546)
Supplement: Table S6 — INRICH Results for Replication Set cutoff top 5%. (DOC) [file pone.0078546.s015.doc]

Table S6. Replication INRICH results

INRICH Results for Replication Set cutoff top 5%

| Target_Size | Int_No | Empirical_P | Corrected_P | Pathway |
| --- | --- | --- | --- | --- |
| 47 | 19 | 0.00249998 | 0.0383883 | REACTOME_REGULATION_OF_ORNITHINE_DECARBOXYLASE |
| 29 | 16 | 0.00260997 | 0.0413877 | ST_ERK1_ERK2_MAPK_PATHWAY |
| 46 | 19 | 0.00278997 | 0.0481864 | REACTOME_STABILIZATION_OF_P53 |
| 43 | 17 | 0.00459995 | 0.09978 | REACTOME_P53_INDEPENDENT_DNA_DAMAGE_RESPONSE |
| 57 | 20 | 0.0119299 | 0.25115 | REACTOME_AUTODEGRADATION_OF_CDH1_BY_CDH1_APC |
| 155 | 52 | 0.0136599 | 0.270946 | REACTOME_MITOTIC_M_M_G1_PHASES |
| 58 | 21 | 0.0143299 | 0.285943 | REACTOME_CYCLIN_E_ASSOCIATED_EVENTS_DURING_G1_S_TRANSITION_ |
| 21 | 11 | 0.0154298 | 0.306939 | REACTOME_ERK_MAPK_TARGETS |
| 44 | 17 | 0.0173398 | 0.332134 | KEGG_PROTEASOME |
| 108 | 36 | 0.0187398 | 0.358928 | REACTOME_CELL_CYCLE_CHECKPOINTS |
| 79 | 30 | 0.0189698 | 0.361528 | REACTOME_G2_M_TRANSITION |
| 61 | 21 | 0.0190998 | 0.367926 | REACTOME_M_G1_TRANSITION |
| 24 | 12 | 0.0205998 | 0.383523 | REACTOME_NUCLEAR_EVENTS_KINASE_AND_TRANSCRIPTION_FACTOR_ACTIVATION |
| 58 | 21 | 0.0232898 | 0.426515 | REACTOME_SIGNALING_BY_WNT |
| 62 | 21 | 0.0260297 | 0.44911 | REACTOME_CDC20_PHOSPHO_APC_MEDIATED_DEGRADATION_OF_CYCLIN_A |
| 154 | 56 | 0.0385996 | 0.581084 | KEGG_ALZHEIMERS_DISEASE |
| 52 | 17 | 0.0699593 | 0.773045 | REACTOME_SCF_SKP2_MEDIATED_DEGRADATION_OF_P27_P21 |
| 103 | 34 | 0.0912191 | 0.853429 | REACTOME_INNATE_IMMUNITY_SIGNALING |
| 30 | 13 | 0.0941391 | 0.859628 | REACTOME_MAPK_TARGETS_NUCLEAR_EVENTS_MEDIATED_BY_MAP_KINASES |
| 75 | 22 | 0.101239 | 0.878824 | REACTOME_DNA_REPLICATION_PRE_INITIATION |
| 161 | 49 | 0.117369 | 0.893221 | REACTOME_METABOLISM_OF_AMINO_ACIDS |
| 22 | 11 | 0.124689 | 0.912817 | BIOCARTA_CCR3_PATHWAY |
| 41 | 16 | 0.126119 | 0.914017 | REACTOME_MAP_KINASES_ACTIVATION_IN_TLR_CASCADE |
| 120 | 37 | 0.130179 | 0.930014 | REACTOME_HOST_INTERACTIONS_OF_HIV_FACTORS |
| 120 | 38 | 0.137009 | 0.946811 | KEGG_LYSOSOME |
| 67 | 23 | 0.141729 | 0.95121 | REACTOME_CENTROSOME_MATURATION |
| 56 | 15 | 0.202698 | 0.979004 | KEGG_ARACHIDONIC_ACID_METABOLISM |
| 49 | 18 | 0.211808 | 0.985003 | SIG_INSULIN_RECEPTOR_PATHWAY_IN_CARDIAC_MYOCYTES |
| 50 | 17 | 0.213668 | 0.986003 | REACTOME_TRAF6_MEDIATED_INDUCTION_OF_THE_ANTIVIRAL_CYTOKINE_IFN_ALPHA_BETA_CASCADE |
| 54 | 18 | 0.217908 | 0.988002 | BIOCARTA_PPARA_PATHWAY |
| 126 | 42 | 0.224248 | 0.989402 | KEGG_NEUROTROPHIN_SIGNALING_PATHWAY |
| 56 | 18 | 0.242498 | 0.992402 | REACTOME_TOLL_LIKE_RECEPTOR_3_CASCADE |
| 59 | 18 | 0.305987 | 0.999 | ST_FAS_SIGNALING_PATHWAY |
| 53 | 20 | 0.307697 | 0.999 | KEGG_NON_SMALL_CELL_LUNG_CANCER |
| 70 | 23 | 0.414656 | 1 | KEGG_PANCREATIC_CANCER |
| 55 | 17 | 0.419396 | 1 | KEGG_RNA_DEGRADATION |
| 59 | 18 | 0.450425 | 1 | REACTOME_LOSS_OF_NLP_FROM_MITOTIC_CENTROSOMES |
| 86 | 25 | 0.483665 | 1 | BIOCARTA_MAPK_PATHWAY |
| 72 | 22 | 0.490335 | 1 | KEGG_CHRONIC_MYELOID_LEUKEMIA |
| 56 | 16 | 0.492365 | 1 | KEGG_ACUTE_MYELOID_LEUKEMIA |
| 51 | 19 | 0.494245 | 1 | KEGG_INOSITOL_PHOSPHATE_METABOLISM |
| 42 | 13 | 0.498945 | 1 | KEGG_BLADDER_CANCER |
| 155 | 50 | 0.523165 | 1 | KEGG_PURINE_METABOLISM |
| 84 | 23 | 0.637404 | 1 | KEGG_PROGESTERONE_MEDIATED_OOCYTE_MATURATION |
| 88 | 25 | 0.688633 | 1 | KEGG_PROSTATE_CANCER |
| 35 | 10 | 0.700313 | 1 | REACTOME_GENERIC_TRANSCRIPTION_PATHWAY |
| 22 | 6 | 0.710113 | 1 | ST_GA12_PATHWAY |
| 48 | 14 | 0.711433 | 1 | REACTOME_SIGNALING_BY_EGFR |
| 38 | 10 | 0.859921 | 1 | BIOCARTA_INTEGRIN_PATHWAY |
| 81 | 9 | 0.864151 | 1 | KEGG_ANTIGEN_PROCESSING_AND_PRESENTATION |
| 63 | 17 | 0.875081 | 1 | SIG_PIP3_SIGNALING_IN_CARDIAC_MYOCTES |
