# Supplementary material for: Whole-Genome Pathway Analysis on 132,497 Individuals Identifies Novel Gene-Sets Associated with Body Mass Index
Source: PLoS One. 2014 Jan 31;9(1):e78546. doi: 10.1371/journal.pone.0078546 (PMC3908858; doi:10.1371/journal.pone.0078546)
Supplement: Table S7 — INRICH Results for Replication Set cutoff top 1%. (DOC) [file pone.0078546.s016.doc]

Table S7 Replication INRICH results

INRICH Results for Replication Set cutoff top 1%

| Target_Size | Int_No | Empirical_P | Corrected_P | Pathway |
| --- | --- | --- | --- | --- |
| 120 | 18 | 0.00510995 | 0.0437832 | KEGG_LYSOSOME |
| 58 | 9 | 0.0130399 | 0.185363 | REACTOME_SIGNALING_BY_WNT |
| 116 | 12 | 0.0246198 | 0.34833 | REACTOME_RNA_POLYMERASE_I_III_AND_MITOCHONDRIAL_TRANSCRIPTION |
| 21 | 5 | 0.0348797 | 0.462108 | REACTOME_ERK_MAPK_TARGETS |
| 77 | 13 | 0.0439896 | 0.545891 | KEGG_FC_EPSILON_RI_SIGNALING_PATHWAY |
| 24 | 5 | 0.0659493 | 0.689062 | REACTOME_NUCLEAR_EVENTS_KINASE_AND_TRANSCRIPTION_FACTOR_ACTIVATION |
| 46 | 6 | 0.0693093 | 0.715457 | REACTOME_STABILIZATION_OF_P53 |
| 27 | 5 | 0.0838992 | 0.779244 | BIOCARTA_GH_PATHWAY |
| 23 | 4 | 0.128619 | 0.89782 | BIOCARTA_TPO_PATHWAY |
| 86 | 11 | 0.131279 | 0.902819 | BIOCARTA_MAPK_PATHWAY |
| 95 | 11 | 0.143849 | 0.917017 | REACTOME_METABOLISM_OF_RNA |
| 90 | 10 | 0.150908 | 0.936613 | REACTOME_LATE_PHASE_OF_HIV_LIFE_CYCLE |
| 22 | 4 | 0.175268 | 0.965407 | BIOCARTA_EIF4_PATHWAY |
| 104 | 11 | 0.192528 | 0.971806 | REACTOME_DNA_REPAIR |
| 41 | 6 | 0.216808 | 0.976805 | KEGG_AMINOACYL_TRNA_BIOSYNTHESIS |
| 52 | 8 | 0.223718 | 0.982404 | KEGG_ENDOMETRIAL_CANCER |
| 26 | 5 | 0.232068 | 0.988402 | BIOCARTA_EDG1_PATHWAY |
| 22 | 3 | 0.240078 | 0.990002 | REACTOME_MTOR_SIGNALLING |
| 56 | 6 | 0.257657 | 0.994601 | KEGG_ACUTE_MYELOID_LEUKEMIA |
| 35 | 5 | 0.305717 | 0.997201 | KEGG_PRION_DISEASES |
| 155 | 13 | 0.324127 | 0.9978 | REACTOME_MITOTIC_M_M_G1_PHASES |
| 38 | 5 | 0.333327 | 0.9984 | REACTOME_TRNA_AMINOACYLATION |
| 108 | 9 | 0.377556 | 0.9996 | REACTOME_CELL_CYCLE_CHECKPOINTS |
| 23 | 2 | 0.501825 | 1 | BIOCARTA_G2_PATHWAY |
| 99 | 11 | 0.556394 | 1 | REACTOME_TRKA_SIGNALLING_FROM_THE_PLASMA_MEMBRANE |
| 73 | 11 | 0.571684 | 1 | KEGG_ADHERENS_JUNCTION |
| 62 | 6 | 0.617554 | 1 | REACTOME_STEROID_METABOLISM |
| 41 | 3 | 0.639194 | 1 | REACTOME_G2_M_CHECKPOINTS |
| 55 | 3 | 0.653773 | 1 | REACTOME_TRANSLATION_INITIATION_COMPLEX_FORMATION |
| 133 | 9 | 0.757452 | 1 | KEGG_UBIQUITIN_MEDIATED_PROTEOLYSIS |
| 36 | 2 | 0.797752 | 1 | REACTOME_ACTIVATION_OF_ATR_IN_RESPONSE_TO_REPLICATION_STRESS |
| 31 | 2 | 0.825782 | 1 | REACTOME_REV_MEDIATED_NUCLEAR_EXPORT_OF_HIV1_RNA |
| 173 | 9 | 0.9582 | 1 | REACTOME_G_ALPHA_I_SIGNALLING_EVENTS |
| 51 | 3 | 0.96945 | 1 | KEGG_INOSITOL_PHOSPHATE_METABOLISM |
