# Supplementary material for: Whole-Genome Pathway Analysis on 132,497 Individuals Identifies Novel Gene-Sets Associated with Body Mass Index
Source: PLoS One. 2014 Jan 31;9(1):e78546. doi: 10.1371/journal.pone.0078546 (PMC3908858; doi:10.1371/journal.pone.0078546)
Supplement: Table S8 — INRICH Results for Replication Set cutoff top 0.5%. (DOC) [file pone.0078546.s017.doc]

Table S8 Replication INRICH results

INRICH Results for Replication Set cutoff top 0.5%

| Target_Size | Int_No | Empirical_P | Corrected_P | Pathway |
| --- | --- | --- | --- | --- |
| 120 | 12 | 0.00764992 | 0.0169926 | KEGG_LYSOSOME |
| 36 | 6 | 0.0160498 | 0.0863827 | KEGG_SPHINGOLIPID_METABOLISM |
| 116 | 8 | 0.0354596 | 0.19856 | REACTOME_RNA_POLYMERASE_I_III_AND_MITOCHONDRIAL_TRANSCRIPTION |
| 62 | 5 | 0.312777 | 0.857828 | REACTOME_STEROID_METABOLISM |
| 90 | 5 | 0.407716 | 0.953609 | REACTOME_LATE_PHASE_OF_HIV_LIFE_CYCLE |
| 51 | 3 | 0.458945 | 0.973005 | REACTOME_TRANSPORT_OF_MATURE_MRNA_DERIVED_FROM_AN_INTRON_CONTAINING_TRANSCRIPT |
| 133 | 6 | 0.572464 | 0.990602 | KEGG_UBIQUITIN_MEDIATED_PROTEOLYSIS |
| 99 | 5 | 0.761662 | 0.9998 | REACTOME_TRKA_SIGNALLING_FROM_THE_PLASMA_MEMBRANE |
