# Supplementary material for: Whole-Genome Pathway Analysis on 132,497 Individuals Identifies Novel Gene-Sets Associated with Body Mass Index
Source: PLoS One. 2014 Jan 31;9(1):e78546. doi: 10.1371/journal.pone.0078546 (PMC3908858; doi:10.1371/journal.pone.0078546)
Supplement: Table S10 — Excess of heritability beyond what is expected based on the proportion of the genome represented for nominally significant pathways at each threshold of significance examined. (DOC) [file pone.0078546.s019.doc]

Supplementary Table 10

Excess of heritability beyond what is expected based on the proportion of the genome represented for nominally significant pathways at each threshold of significance examined

| **Sample** | **All Pathways** | **Top 10% Enriched** | **Top 5% Enriched** | **Top 1% Enriched** | **Top 0.5% Enriched** |
| --- | --- | --- | --- | --- | --- |
| % Heritability | 19.76% | 5.65% | 7.73% | 1.64% | 0.00% |
| % Genome | 13.06% | 3.45% | 3.29% | 2.88% | 1.32% |
| Standard error | 9.13% | 5.01% | 5.11% | 4.26% | 2.78% |
| P-value | 0.46 | 0.66 | 0.39 | 0.77 | 0.63 |
