# Supplementary material for: Whole-Genome Pathway Analysis on 132,497 Individuals Identifies Novel Gene-Sets Associated with Body Mass Index
Source: PLoS One. 2014 Jan 31;9(1):e78546. doi: 10.1371/journal.pone.0078546 (PMC3908858; doi:10.1371/journal.pone.0078546)
Supplement: Table S11 — Replication set population statistics. (DOC) [file pone.0078546.s020.doc]

Table S11. Replication set population statistics

| **Sample** | MESA | GENEVA T2 Diabetes | CARDIA | **TOTAL** |
| --- | --- | --- | --- | --- |
| **Male/Female** | 1074/917 | 2334/3111 | 563/633 | 3971/4661 |
| **Mean Age (yrs)** | 63.1 | 57.24 | 27.57 |  |
| **N_Subjects** | 1991 | 5445 | 1196 | 8632 |
| **Mean BMI (kg/m^2^)** | 27.79 | 26.97 | 23.66 |  |
